# Pygmy blue whale feeding drives greater nocturnal than diurnal song production

**DOI:** 10.1098/rsos.260954

**Published:** 2026-10-28

**Authors:** Andrew M. Davenport, Michele Thums, K. Curt S. Jenner, Micheline-Nicole M. Jenner, Luciana C. Ferreira, Christine Erbe, Ryan Leadbetter, Robert D. McCauley

**Affiliations:** ^1^ Centre for Marine Science and Technology, Curtin University, Bentley, Western Australia, Australia; ^2^ Centre for Whale Research (WA) Inc., Fremantle, Western Australia, Australia; ^3^ Australian Institute of Marine Science, Indian Ocean Marine Research Centre, University of Western Australia, Crawley Western Australia, Australia

**Keywords:** pygmy blue whale, *Balaenoptera musculus brevicauda*, foraging, migration, behaviour, song, eastern Indian Ocean, biotelemetry, passive acoustic monitoring, diel pattern

## Abstract

Effective management of threatened and migratory species requires monitoring of critical behaviours, such as foraging. Passive acoustic monitoring can provide cost-effective, long-term observations on the relative abundance of vocal animals across large spatial scales. However, linking specific sounds to behavioural contexts can be challenging, especially for cryptic and aquatic species. This study combined passive acoustic and biologging data to explore whether observed periods of greater nocturnal than diurnal song production were linked to feeding by eastern Indian Ocean pygmy blue whales, *Balaenoptera musculus brevicauda.* The proportion of time available for singing was modelled as a function of geographical region, diel period and the presence or absence of feeding dives with generalized linear mixed models, based on 130.1 days of diving data. Top-ranked models indicated less time was available for singing during diurnal feeding periods compared to nocturnal feeding periods and non-feeding periods during the day and night. These findings suggest that diurnal feeding by pygmy blue whales reduces the time available for song production. Therefore, greater nocturnal than diurnal song production may provide an acoustic indicator of population-level feeding activity for pygmy blue whales between the Perth Canyon and offshore of the northwest shelf of Western Australia.

## 1. Introduction

Blue whales (*Balaenoptera musculus*) are a listed threatened and migratory species in Australia's Environment Protection and Biodiversity Conservation Act 1999 (EPBC Act). Under the EPBC Act, the Blue Whale Conservation Management Plan considers both subspecies that occur in Australian waters to be endangered, the Antarctic blue *Balaenoptera musculus intermedia* and pygmy blue whale, *Balaenoptera musculus brevicauda* [1]. The most abundant blue whale population in waters to the south and west of the Australian continent is the eastern Indian Ocean pygmy blue whale (EIOPBW), with estimates of 500–2000 individuals in the early 2000s [2,3]. The population remains endangered owing to calculations of a slow recovery rate (4.3% per annum based on passive acoustic monitoring) from historical whaling that took an estimated 2593 EIOPBWs [4,5]. The EIOPBW migrates from subantarctic waters south of Australia and in the southern Indian Ocean, through the eastern Indian Ocean past the west coast of Australia, to tropical waters of Southeast Asia [6,7]. Along their migration, EIOPBWs encounter various offshore economic sectors (oil and gas extraction, major shipping routes and future renewable energy) in the Bass Strait, Great Australian Bight, Geographe Bay and the northwest continental shelf of Western Australia [8,9]. The Australian EPBC Act requires the management of potential threats through the Blue Whale Conservation Management Plan [1]. One of the key actions listed is ‘Anthropogenic noise in biologically important areas will be managed such that any blue whale continues to utilise the area without injury, and is not displaced from a foraging area’ [1, p. 37]. There remains a limited understanding about critical foraging behaviours for most of the areas where the EIOPBW migration overlaps with marine economic sectors and how anthropogenic noise may impact foraging.

EIOPBWs use various productive areas throughout their distribution, rather than relying on a single seasonal food resource [10]. From November to May, EIOPBWs forage in the Great Southern Australian Coastal Upwelling System and throughout the subtropical convergence zone [11–15]. In February–July, feeding has been recorded at various locations along and offshore of the west and northwest continental shelves of Western Australia, such as in the Perth Canyon and near Ningaloo [10,16]. Suitable foraging habitat for EIOPBWs has also been predicted in the offshore regions north of Ningaloo [17,18]. From June to September, there have been indicators of foraging within and around the northern terminus of the EIOPBW migration (primarily in the Timor Trough, Savu Sea and Banda Sea) [10,18,19], with visual confirmation of an EIOPBW feeding near the surface in the Wetar Strait [20]. There are limited data to determine whether foraging behaviour occurs during the southbound migration in September–November before EIOPBWs return to southern feeding areas [7,14,17]. Communication has been suggested to play a key role in navigation of such vast areas to find food and conspecifics, through acoustic signatures linked to distinct behavioural motivations [21–23].

Blue whales produce vocalizations that are typically characterized as non-song sounds or as units comprising stereotypical song [24–26]. Non-song sounds, such as frequency-modulated down-sweeps, or D-calls, have been associated with feeding blue whales [21,22]. Down-sweeps are not readily distinguishable to species or population levels as they display a continuum of characteristics within and across taxonomic groups, so assigning calls is especially difficult with more than three sympatric species or if humpback whales (*Megaptera novaeangliae*) are present [27,28]. Separating down-sweeps between specific taxonomic groups (e.g. sei *Balaenoptera borealis*, fin *Balaenoptera physalus* and blue whales) is possible given appropriate training data from acoustic biologging tags or strict selection criteria, often in combination with machine-learning classifiers [29–34]. Meanwhile, song characteristics are distinguishable for blue whale subspecies and sub-populations, aiding efforts to map population distributions [35,36]. The EIOPBW song comprises three units arranged in various sequences distinct from other blue whale populations and sometimes even distinguishable between individuals singing at the same time [37–39]. Song was proposed to function as a male reproductive display to attract female mates [25], which enables monitoring of breeding effort across seasons and years [40]. Song has been received by passive acoustic monitoring stations almost continuously during singing seasons from ranges of up to 200 km in open ocean waters [41], enabling strategic monitoring of large spatial extents [42–45]. Some authors have identified a reduction in song during the day compared to the night and hypothesized that foraging effort may explain the pattern [46,47].

Feeding behaviours and sound production by blue whales tend to exhibit diel patterns [48]. Blue whales (*B. m. musculus*) in the northeast Pacific Ocean have been recorded intensively feeding during the day off the California coast [49,50]. A cessation of feeding at night was thought to be driven by a reduction in euphausiid prey density as the prey undergo a diel vertical migration, dispersing in shallower depths at night to forage on phytoplankton [46,47]. This nocturnal dispersion may have sufficiently reduced the prey density so that the cost of lunge feeding by the whales exceeds the energy they would gain [51–54]. During the night, these whales either rested near the surface [21,50] or swam in large, shallow loops to return to the feeding patch by morning [55]. Based on tagging records, Oestreich *et al.* [56] observed that whales sang more at night compared to the day. This day–night singing pattern resulted from a predominance of feeding dives during the day, while song was only produced on non-feeding dives that were more prevalent at night [56]. Two whales demonstrated an acoustically detectable transition from foraging to migration in their song production, tending to sing more at night when they were on the foraging ground and more during the day after embarking on migration [56]. However, greater singing during the day compared to the night did not manifest at the soundscape-scale during collective migration; rather the day-to-night song ratio of the population was roughly even [56,57]. Diel patterns have been observed in EIOPBW song at their foraging areas in the Perth Canyon [58] and the subtropical convergence zone [13], as well as the southwest continental slope off Cape Leeuwin [41]. A passive acoustic indicator of behaviour based on day–night singing ratios validated for EIOPBW foraging areas would improve our ability to monitor the feeding effort of this population.

To support the application of the day–night singing ratio as an indicator of feeding behaviour for the EIOPBW population, this study explored whether periods of greater EIOPBW song at night compared to the day could be linked to diurnal feeding. The hypothesis that feeding reduces the time available for singing compared to non-feeding behaviours was tested in a mixed-effects modelling framework on a diving biotelemetry dataset. Day versus night periods were included as a factor in the model to reveal diel patterns in the time available for singing as a function of diving behaviour. To identify whether patterns in the time available for singing were geographically consistent, five regions were delineated, between the southwest of Western Australia and the Banda Sea of Southeast Asia. To demonstrate the occurrence of EIOPBW day–night singing ratios in the foraging aggregation at the Perth Canyon and across other foraging areas along their northward migration to Southeast Asia, the day–night singing ratios in passive acoustic monitoring datasets collected from these regions were explored.

## 2. Methods

### 2.1. Biotelemetry dataset

This study used existing datasets from 12 pygmy blue whales instrumented with telemetry tags between 2014 and 2024 in Western Australia, recording diving and movement behaviours (table 1). Three types of tags were used (all by Wildlife Computers, Redmond, Washington, USA): one Whale Lander tag, nine miniPAT (pop-up archival transmitting tags) and nine LIMPET tags (Low Impact Minimally Percutaneous Electronic Transmitters, type SPLASH10-F-333). These tags provided location, depth and other data in various configurations and sampling rates dependent on the tag type, programming and if the tag was physically recovered to access the archive.

**Table 1.** Metadata for the 12 whales tagged. (Note: whale ID is the tag deployment date in YYMMDD format, with letter suffixes when multiple whales were tagged on the same day. Sample interval is the time interval in seconds between depth samples. Length of whales (where available) was in metres. The total deployment duration (Dur_T_) in days and the summed duration of recovered depth data via satellite transmission or physical recovery (Dur_R_). Dives are the count of individual dives in the tag record after data processing. Surf is the percentage of time (excluding incomplete dives) that was spent at the surface.)

whale ID	tag (SN)	location tagged	final location	sample interval (s)	sex	length (m)	Dur_T_ (d)	Dur_R_ (d)	dives	surf (%)
140430^a^	Lander (95805)	Perth Canyon	Abrolhos Canyons	0.25	U		7.6	7.6	1621	10.7
210427-A	miniPAT (214432)	Perth Canyon	Bremer Canyon	1	U		30.8	30.8	11418	20.5
210427-B	miniPAT (214430)	Perth Canyon	Exmouth Plateau	75	U		30.5	16.8	3245	16.8
220424	miniPAT (233088) LIMPET (233087)	Perth Canyon	Perth Canyon	1	F	19.9	10.6	10.6	2158	21.9
220430	miniPAT (214431) LIMPET (233084)	Perth Canyon	Perth Canyon	1	M		4.7	4.7	1083	18.2
220501	miniPAT (233091) LIMPET (233086)	Perth Canyon	Steep Point	75	F	19.8	27.6	11.0	1246	7.0
220507	miniPAT (233095) LIMPET (233079)	Perth Canyon	Steep Point	75	M		26.2	14.3	1603	18.7
220508	miniPAT (233102) LIMPET (233082)	Perth Canyon	Banda Sea	75	F	21.2	40.5	21.6	1493	31.4
220509	miniPAT (233101) LIMPET (233083)	Perth Canyon	Perth Canyon	1	F	18.4	3.8	3.8	1231	32.4
230523	miniPAT (233096) LIMPET (233080)	Ningaloo	Exmouth Plateau	75	F	21.7	3.9	3.9	714	5.9
240528-A	LIMPET (243740)	Ningaloo	Exmouth Plateau	75	U		13.6	4.1	397	2.4
240528-B	LIMPET (243738)	Ningaloo	Exmouth Plateau	75	M		4.9	0.9	126	3.1

^a^ ID 140430 was the singing whale in Davenport *et al.* [59].

The Whale Lander tag had a FastLoc GPS Receiver (Wildtrack Telemetry Systems Ltd., Leeds, UK), 16 Hz triaxial accelerometer, 4 Hz pressure sensor (± 1 m), 4 Hz wet–dry sensor, 4 Hz light sensor, 2 Hz triaxial magnetometer and a 2 Hz fast-response thermistor. The miniPAT tags had a 1 Hz pressure sensor (± 0.5 m), 1 Hz light sensor, 1 Hz temperature sensor (± 0.05°C) and an 8 Hz triaxial accelerometer. The LIMPET tags had pressure (± 0.5 m) and temperature sensors (± 0.05°C) sampled once every 10 s and a FastLoc GPS Receiver.

MiniPAT acceleration data were processed onboard to archive the summary metric mobility, which were only accessible if the tag was recovered (*n* = 4) [60]. When the miniPATs detached from the whale and were floating, they transmitted data packets of 45 samples per hour (75 s interval time-series, which is the maximum allowable for transmission) of depth and the activity-time-series (*n* = 5) [60]. For miniPAT deployments in 2021, light data were processed to provide geolocation estimates at sunrise and sunset. LIMPET tags were deployed simultaneously with miniPATs in 2022 and 2023 to provide a higher quality of location data, being programmed to transmit FastLoc GPS location data every hour. In 2024, only LIMPETs were deployed, programmed to transmit a 75 s time-series of depth for one fortnight after deployment, in addition to FastLoc GPS location data every 4 h. For additional details on the tag deployments, sex determination from biopsy samples and size estimation from aerial imagery, see Owen *et al.* [61], Davenport *et al.* [59], Thums *et al.* [10,17] and Russell *et al.* [62].

### 2.2. Dive classification

For each whale, the time-series of tag data were delineated into dive periods and classified into behavioural types [10,59]. Thums *et al.* [10] employed the R [63] package *diveMove* [64] to delineate dives with a 1.5 m surface threshold on the miniPAT datasets from 2021 to 2023, then applied a supervised random forest model [65] to classify dives based on the agreed-upon manual classifications from two analysts, with the modelling undertaken on 1 and 75 s sampled data separately. The random forest model for the 75 s time-series data did not outperform the manual classifications, so manual classifications agreed upon by the analysts were used for this study. This *diveMove* and manual classification approach was applied to the 2024 LIMPET 75 s depth time-series datasets. Davenport *et al.* [59] created a customized data processing pipeline for the Whale Lander tag data (whale 140430), classifying behaviour using an automated decision tree based on dive metrics. To create a standardized schema from these three distinct sources of diving behaviour data, we adopted class descriptions matching the 1 s miniPAT schema [10] and grouped the synonymous classes from the Whale Lander and 75 s miniPAT datasets (table 2).

**Table 2.** The standardized dive class schema and definitions, along with the classes from previous work by Thums *et al.* [10] for 1 and 75 s time-series data and from Davenport *et al.* [59] for the higher resolution Lander tag.

class	definition	1 s class	75 s class	Lander class
interblow	shallow (< 4 m) and short (< 60 s)	interblow	N/A	intra-surfacing
rest	shallow (< 12 m) and very low mobility (< 40) or fluking rate (<0.02 Hz)	resting	N/A	resting
travel	square or round shaped with depth <4 0 m	travelling	travelling; too short	slow travel; fast travel; racing
explore	V-shaped dive to depths greater than surrounding dives	exploratory	exploratory	exploratory
forage	square or round shaped with depth > 40 m	foraging	foraging	shallow searching; deep searching
feed	square-shaped with depth > 40 m and undulations in depth usually corresponding with spikes in mobility, activity-time-series or lunge feeding events in the Lander data. Repetitive (three or more consecutive) V-shaped dives to 40–100 m for 150–300 s were considered to be bouts of surface feeding	feeding	feeding	deep searching (feeding)
porpoise	shallow (< 12 m) and short (< 60 s) dives with very high mobility (> 400), activity-time-series (> 40) or fluking rate and amplitude (0.19 Hz and 3.8°)	porpoising	surface active	porpoising

To derive more accurate time budgets of behaviour, data were curated further to identify combination dives (when *diveMove* defined a single dive when there were likely to be two dives often of different class owing to the coarse 75 s sample interval) and square root dives (two-part dives that were a combination of an exploratory dive and a travel dive). Combination and square root dives were split into discrete parts that resemble distinct dive behaviours, such as travelling and exploring for square root dives. This process involved manually reviewing every dive in the 75 s datasets and every exploratory and foraging dive in the 1 s datasets. For individuals with 75 s datasets, 3–32% of dives were identified as having the first and/or last data points of the dive not at the surface, which were manually corrected. Dives classified as incomplete in Thums *et al.* [10] (if there was a missing start or end phase of the dive owing to data transmission gaps) were reclassified to a behaviour if there were enough samples (> 4 samples, equivalent to 300 s) to be confident in the dive behaviour assignment. See the supplementary material for more detail and examples of the curation of diving data. For the 75 s datasets, the classes *other*, *disagree* and the remaining *incomplete* dives were removed from all further analysis.

### 2.3. Data preparation

To explore diel patterns in behaviour, the diving dataset was summarized into day and night periods based on the local sunset and sunrise times (0° solar elevation) for daily estimates of whale locations. Location estimate processing followed the methods in Thums *et al.* [10,17]. Briefly, a correlated random walk model was applied to all location data to account for location error and standardize sample intervals in the R package *foieGras* [66]. Datasets with a similar average number of daily location estimates were modelled together. Location tracks were truncated to the date range when diving data were available.

The time-series of diving data was summarized into diel periods representing day (from sunrise to sunset) and night (from sunset to sunrise). Sunrise and sunset times were calculated for each day from the mean whale location between 06.00 and 18.00 local time (UTC + 8 h). Dives were assigned to the diel period in which they began. The total duration of diving data in each diel period included time at the surface between dives. Diel periods with less than 1 h total duration of diving data were removed from the statistical analysis (*n* = 9), to avoid periods overly biased by single dives. The remaining dataset had 406 diel periods.

Five geographical regions were defined to represent distinct ecological and feeding areas, with the intention of distinguishing the diel data into areas where observed behaviours may differ (figure 1). Diel periods were assigned a region according to the mean location during the period. The five regions were the Southwest (> 33° S), the Perth Canyon (33–30° S), the Midwest (30–25° S), the Northwest (25–19° S) and the North (< 19° S).

**Figure 1.**
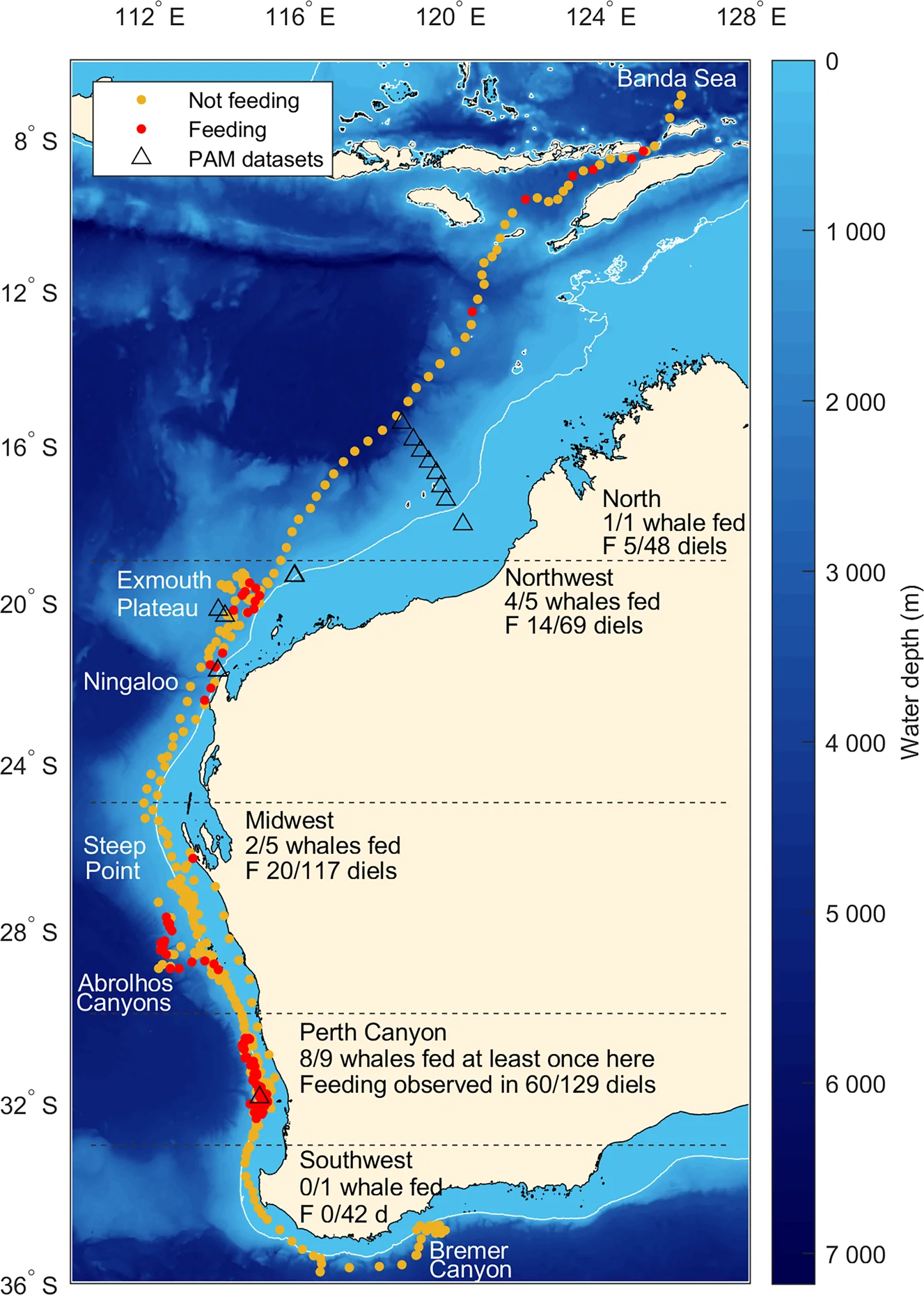
Map of midday and midnight locations representing diel periods where feeding dives did (red dots) and did not (orange dots) occur. Horizontal dashed black lines indicate the borders between identified regions, which are labelled in the Australia mainland area. Also noted on the map in text are the sample statistics for the number of whales feeding out of the total number of whales and the number of diel periods (diels) that feeding was observed in out of the total number of periods. The solid black and white lines indicate the 0 and 200 m depth contours.

### 2.4. Statistical analysis

A generalized linear mixed model (GLMM) framework was used to determine whether periods of nocturnal prevalence in EIOPBW song could be the result of a diurnal feeding preference and whether there was any influence of geographical region. To do this, a proxy for the proportion of time available for singing (TAS) was defined as the response variable. The variable TAS was the proportion of time in each diel period spent in *travel* or *rest* dives, which are synonymous with stereotypical singing dive profiles in blue [22,59], fin [67] and humpback whales [68], probably because they were performed at depths where song production was physiologically efficient [69]. It was assumed that time spent at the surface (< 1.5 m) was for the sole purpose of breathing and not spent sleeping, resting, logging or travelling and therefore was not considered to be time that was available to sing.

TAS was modelled as beta distributed with a logit link function (a beta GLMM), which is appropriate for proportional response variables between 0 and 1, excluding boundary values (data points equal to 0 or 1). To prevent numerical errors for three periods that had a zero TAS value, 0.001 was added. This addition was justifiable as it was improbable that the TAS was ever exactly zero for an EIOPBW. The statistical analysis was conducted with the R package *glmmTMB* [70], as it could fit the beta distribution in a mixed-effects modelling framework, which includes support for fixed and random effects.

The beta model was parameterized in terms of the mean and precision (dispersion) parameters; each was conditional on an independent covariate expression. Three covariates were considered for their fixed effects on the mean and precision of the beta model: diel period (diel) as a factor (day or night), presence or absence of a feeding dive in the period (feed) as a logical expression and the region within which the period was located (region) as a factor. As the individual whale effect was not the focus of the model and individuals were assumed to be a random sample of the population, whale ID was considered as a random effect on the intercept and on each fixed effect [71].

Model selection to determine the covariate expressions for the mean and precision parameters that explained the greatest variation in the data was based on Akaike information criterion (AIC) values, following a stepwise approach where all options of single term additions were compared per step [72]. This approach was applied sequentially to:

(i) select the fixed-effects expression for the mean parameter (with a constant precision parameter and without random effects),
(ii) select the fixed-effects expression for the precision parameter (with the mean expression from (i) and without random effects), and
(iii) select the random-effects expression for both mean and precision models (with the optimal mean and precision expressions from (i) and (ii)).

To validate the final model fit, the distribution of Pearson residuals was visualized using box plots across levels of each covariate (fixed and random effects), as well as scatter plots of the residuals against the index of observation. Violin plots from the R package *ggplot2* [73] were reviewed for the fit of predicted versus raw data. Spatial autocorrelation was assessed using residuals plotted against location, and temporal autocorrelation was assessed for each whale with the autocorrelation function *acf* from the R package *stats* [63].

Additional modelling was conducted to explore the importance of an interaction term between the covariates ‘region’ and ‘feed’ in two subsets of the data that excluded specific regions. The absence of feeding in the Southwest region prevented the interaction term between region and feed from being included in the full dataset model (M1), while the North region only contained data from one whale. The first subset included all regions except the Southwest region (M2). The second subset included the Perth Canyon, Midwest and Northwest regions (M3). The starting expressions for models M2 and M3 were taken as the final selected expression for M1. A forward-stepwise model selection approach was then followed to find the fixed-effects of the mean and precision expressions that resulted in the lowest AIC value.

### 2.5. Day–night singing ratio in passive acoustic monitoring data

Passive acoustic monitoring datasets were investigated for daily periodicities in EIOPBW singing to demonstrate that day–night singing ratios occur in passive acoustic monitoring data collected from foraging areas. There were 10 datasets from the Perth Canyon spanning a cumulative 1224.2 days of deployment (as opposed to recording days), 11 datasets from the Northwest (1770.2 days) and 15 from the North region (1895.3 days) (supplementary material, table S1). Datasets had various recording duty-cycle regimes; see detailed deployment and data processing methodologies in the supplementary material and in McCauley *et al.* [4]. The second unit (U2) of the EIOPBW song was detected in each dataset using an automated algorithm targeting the third harmonic [4]. Manual validation was conducted on each dataset.

The singing rate was defined as the number of U2 calls per hour (call h^−1^), calculated as the number of calls divided by the fraction of the hour that was recorded. The number of calls and the number of hours of recording were extracted for each diurnal (sunrise to sunset) and nocturnal period (sunset to sunrise). For each dataset, a daily, a diurnal and a nocturnal series of singing rate were calculated. The day–night singing ratio was calculated as the nocturnal rate divided by the diurnal rate, only when the daily singing rate exceeded a threshold of 5 call h^−1^ (to constrain the analysis to only when singers were abundant). To de-noise the dataset for the purpose of visualization only, the singing rate and day–night singing ratios were also calculated with a 7-day running average (sampled daily).

To characterize the singing behaviour across datasets, summary metrics were calculated from the daily singing rate and day–night singing ratios. These included the number of days that the singing rate exceeded the 5 call h^−1^ threshold (singing days) and the number of days that the day–night singing ratio exceeded 1 (representing nocturnal singing days as a ratio > 1 happens when the nocturnal call rate exceeds the diurnal rate). Bouts of consecutive nocturnal singing days were identified to explore the persistence of potential foraging, which were summarized for each dataset as the total number of bouts and the statistics of bout durations (25th percentile, median, 75th percentile and maximum). The proportions of singing days that had greater nocturnal activity and the proportions of nocturnal singing days that were part of bouts were compared across combinations of regions and migration directions. Both the northbound and southbound migrations were captured in the acoustic datasets that collectively spanned all days of the year for the North and Northwest regions. Only the northbound migration was represented in Perth Canyon acoustic datasets.

## 3. Results

### 3.1. Model of time available for singing

The model for the full dataset (M1) with the lowest AIC included a region effect and an interaction effect between diel period and the presence of feeding (i.e. *diel:feed*) (supplementary material, tables S2 and S3). M1 comprised a mean expression *TAS ∼ region + diel + feed + diel:feed + (diel|ID)* and a precision expression *∼ region + diel + feed + diel:feed + (feed|ID)*. The term *(diel|ID)* in the mean expression indicated that the diel fixed effect was conditional on the random effect of whale ID.

The fit of M1 shows that the proportion of time available for singing when feeding during the night was similar to non-feeding periods during the day and night regardless of region (excluding the Southwest as no feeding behaviour was recorded in this region) (figure 2*a*). A low TAS was predicted for days with feeding-present, with less than 0.422 at a 95% confidence interval (CI) across all foraging regions. By contrast, all non-feeding periods were predicted to be greater than 0.526 (95% CI) excluding the Southwest region, which had a lower predicted TAS (>0.414, 95% CI) based on data from one individual.

**Figure 2.**
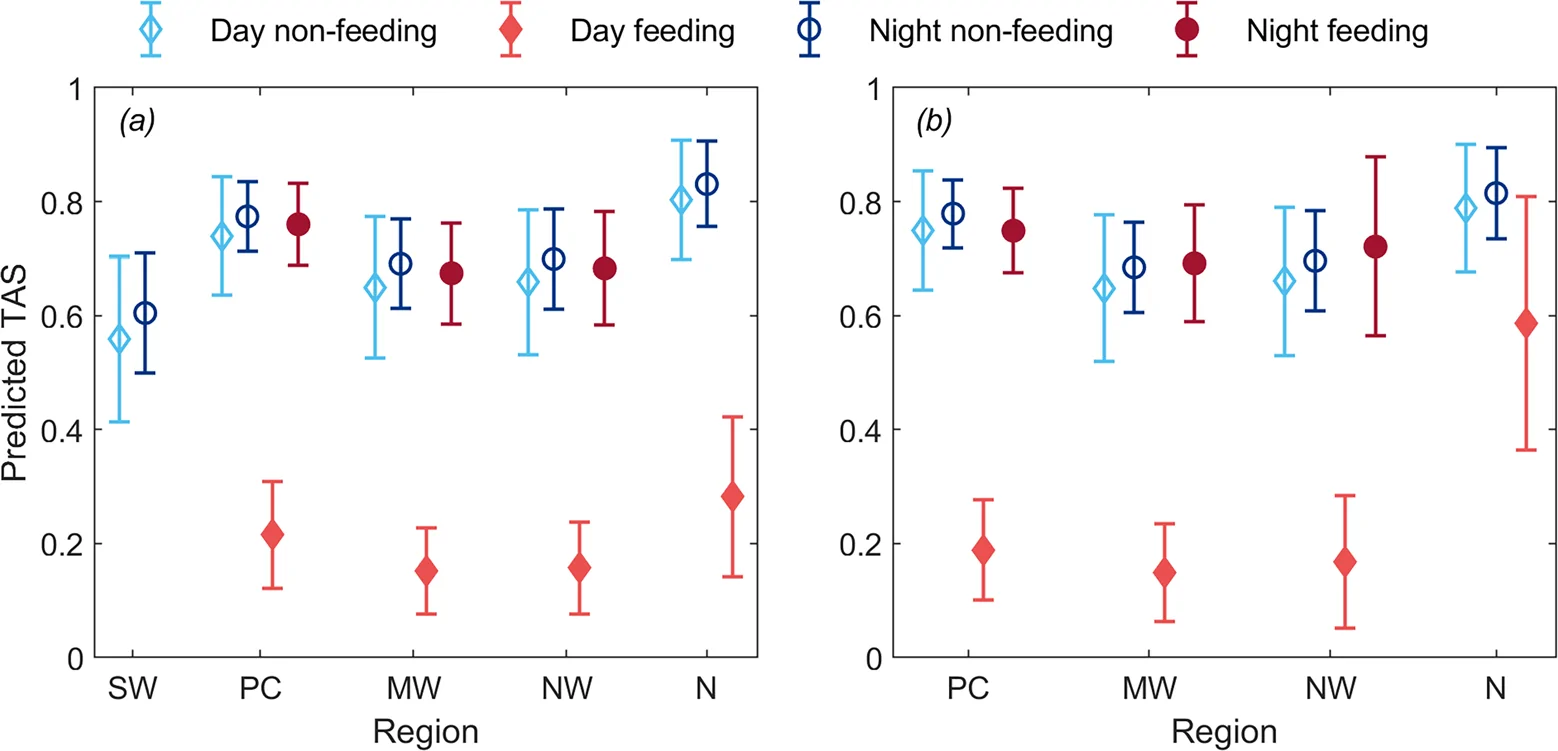
Relationship between the predicted proportion of time available for singing (TAS) and the independent variables (region, diel and feed) in the best M1 model, including all data (*a*) and for the subset M2 model excluding the Southwest region (*b*). Error bars are the 95% confidence interval (calculated as 1.96 times the standard error). Symbology distinguishes presence and absence of feeding dives and the diel period. Regions are Southwest (SW), Perth Canyon (PC), Midwest (MW), Northwest (NW) and North (N).

Model validation of M1 showed a good fit with negligible residual patterns (see the supplementary material for the full modelling script, including markup). Violin plots of predicted values from M1 versus the raw distributions supported the generally good fit of the model, except for feeding-present days in the North region (supplementary material, figure S1). The removal of the Southwest region (M2) from the dataset enabled the inclusion of an interaction effect between region and feeding in the mean expression *TAS ∼ diel + feed + region + diel:feed + region:feed + (diel|ID*), improving the model fit for this subset (supplementary material, tables S4 and S5, figure S2). The predictions of M2 compared to M1 changed noticeably for daylight feeding in the North region but not for other covariate combinations (figure 2*b*), confirming the poor fit of this factor combination in M1. Data exploration revealed that three of the five feeding-present days in the North region had feeding time proportions of less than 0.2 and an overall lower distribution of feeding time per feeding-present day than the other regions, which had similar distributions to one another (supplementary material, figure S3). The feeding presence and region interaction term was not included in the best model for the subset with only the Perth Canyon, Midwest and Northwest regions (M3), which had the same specification as for the full dataset (supplementary material, tables S6 and S7, figure S4).

### 3.2. Hourly patterns in feeding and depth

Histograms of the time of day that feeding dives were observed in each region show that most feeding dives occurred during the daylight hours in all regions, explaining the predicted daily pattern in TAS (figure 3). Some feeding dives occurred during the twilight hours in all regions and during the middle of the night in the Northwest region. The data-rich Perth Canyon region showed a strong pattern with a slow build-up after dawn, reaching a plateau at midday, then an abrupt cessation around dusk. The other regions had fewer feeding periods reducing the confidence in any hourly feeding patterns observed.

**Figure 3.**
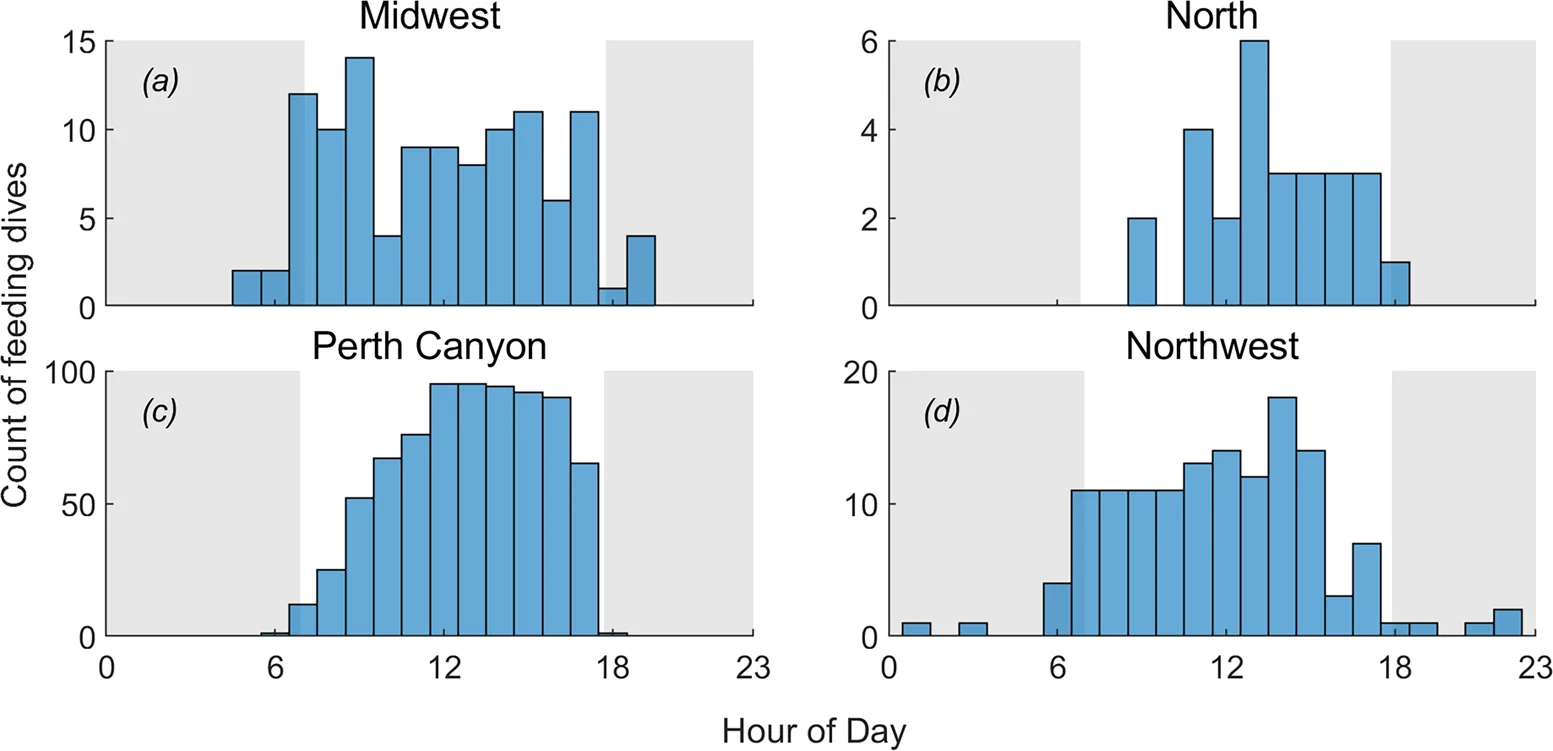
The frequency of feeding dives in each hour of the day across the four regions (Midwest (*a*), North (*b*), Perth Canyon (*c*) and Northwest (*d*)) where feeding was observed, with the average night periods shaded light grey.

Feeding behaviour strongly influenced the time spent at depths where vocalizations are known to be produced (< 40 m) [22,59], as indicated by the time spent in depth bins by region, behaviour and time of day (figure 4). There was a distinct diel periodicity in time at depth on feeding-present days for regions Perth Canyon, Midwest and North. For the Northwest, time spent between 10 and 100 m increased in the afternoon on feeding days. For Perth Canyon, time at depths between 100 and 500 m comprised approximately 60% of time in daylight hours, while at night the time in the top 40 m of the water column increased to more than 90% of time. A similar pattern occurred for the Midwest and North regions, however with greater time spent shallower at night. When not feeding, the whales spent about 90% of time shallower than 40 m regardless of light regime. Time shallower than 10 m was greater in the Northwest and North regions (approx. 50 and approx. 75%, respectively) than in the Southwest, Perth Canyon and Midwest regions (approx. 30, approx. 30 and approx. 20%, respectively).

**Figure 4.**
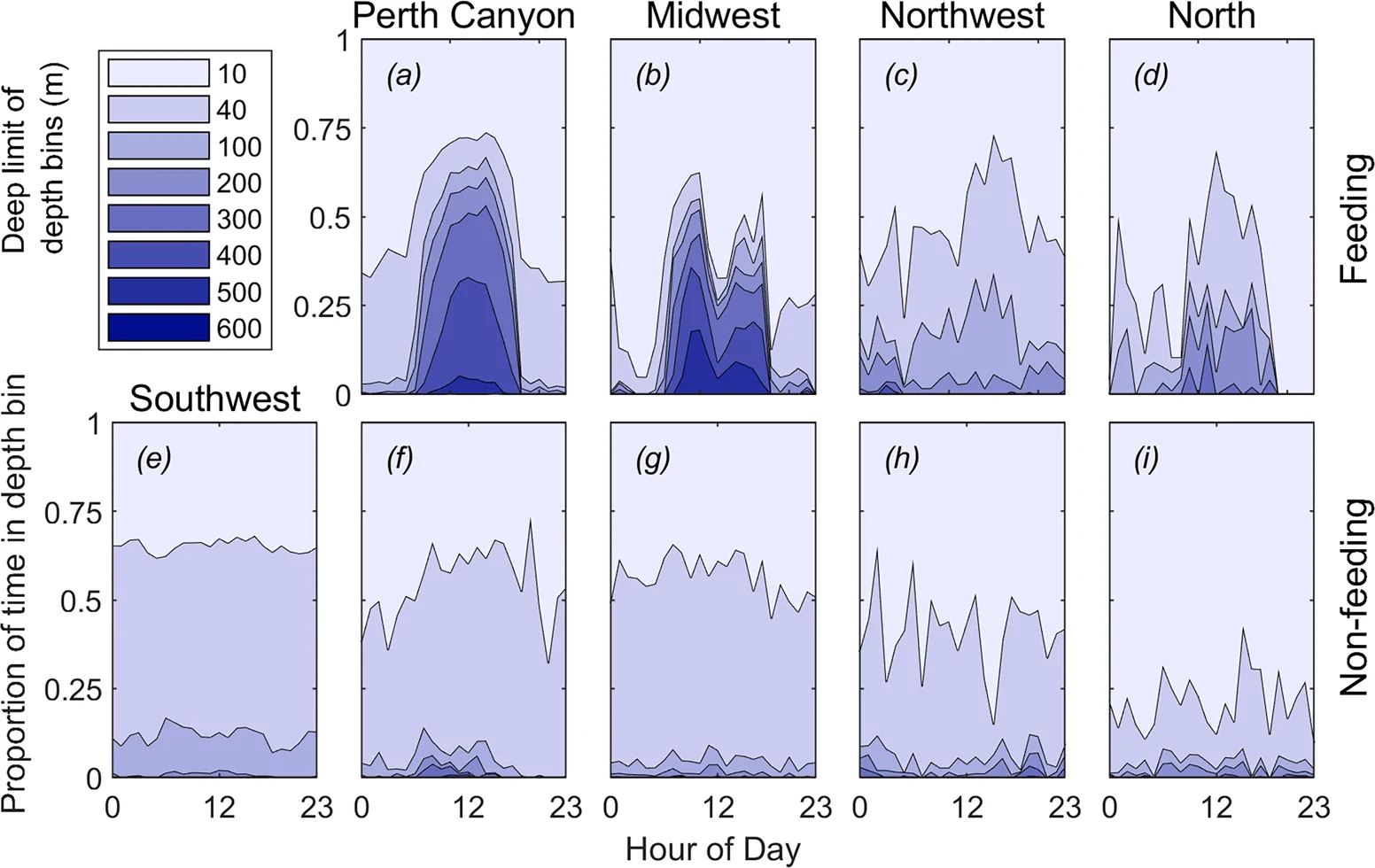
Proportion of time in depth bins (colour scale label indicates deeper edge of bin) by hour of day for 24 h periods when feeding was observed (*a*–*d*) and not observed (*e*–*i*). Columns indicate regions. Day units were identified from sunrise to sunrise the following day.

### 3.3. Non-feeding behaviours influencing time available for singing

For non-feeding periods, violin plots of raw data versus predicted values from M1 revealed bimodal or skewed distributions with long lower tails in the raw data, particularly in regions Midwest, Northwest and North (figure 5). The raw distribution of TAS for non-feeding days in the Northwest region was bimodal. Predictions for whales 220508 and 210427-B were in the lower mode of this distribution and had the lowest predicted TAS for all non-feeding periods. To explore the effect of these whales on the raw distribution, data from these individuals were excluded from the violins of raw TAS in the second row of figure 5, which mostly removed the lower modes or tails. Whales 220508 and 210427-B had lower distributions of raw TAS than the other whales in the Midwest and Northwest (supplementary material, figure S4). Whale 210427-A in the Southwest region also demonstrated a low predicted TAS and raw TAS distribution.

**Figure 5.**
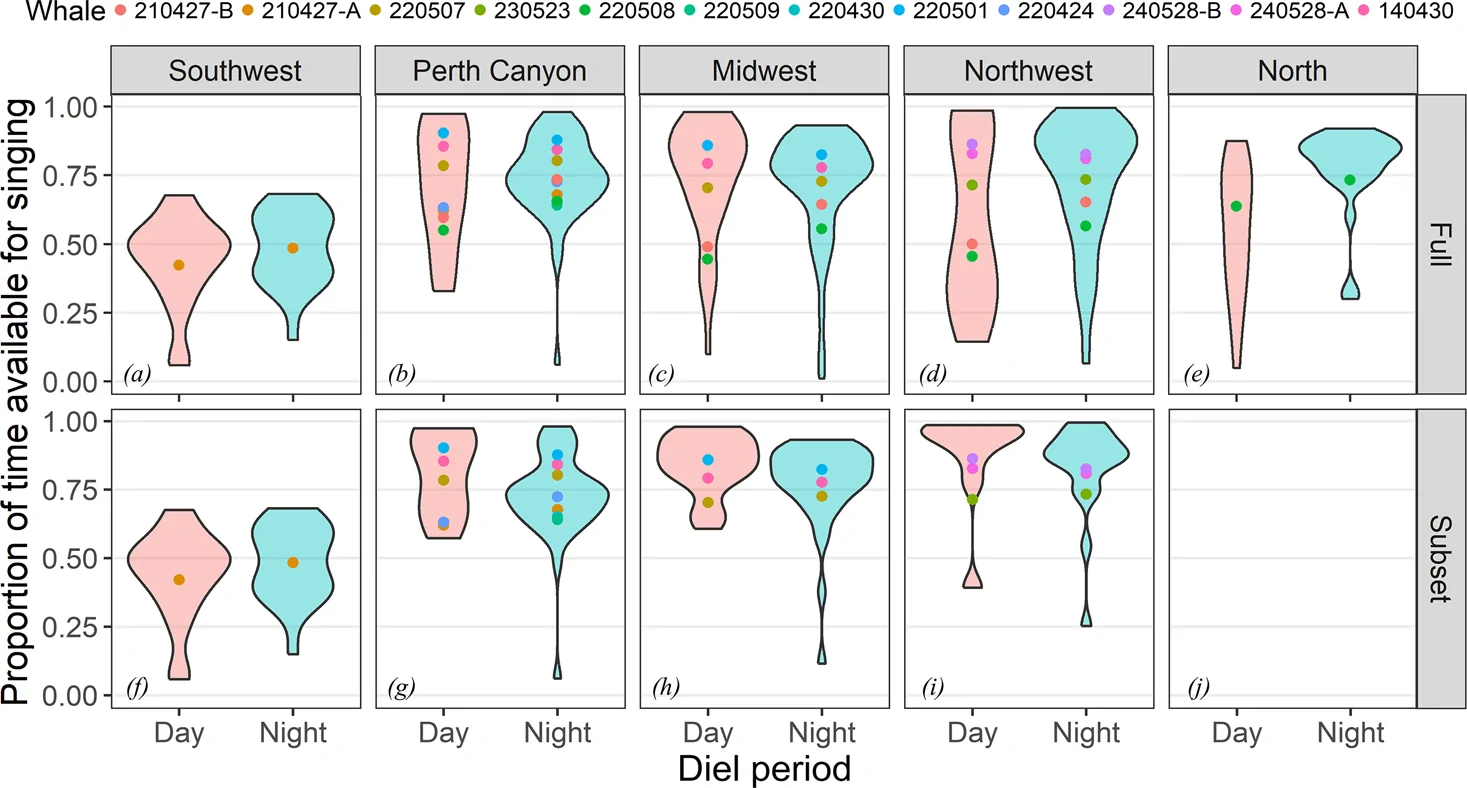
Predicted TAS values (points) from M1 and the raw distribution (violin) for only non-feeding periods by region and diel period, for the full dataset (*a*–*e*) and after excluding 220508 and 210427-B (*f*–*j*). Predicted points for each whale ID are coloured by the ID.

To identify the non-feeding behaviours that whales 210427-A, 210427-B and 220508 were undertaking to reduce TAS, time-series of surface and dive-type time proportions were plotted for these subsets (figure 6). In the absence of feeding, TAS less than 0.6 was either a result of extended time spent at the surface (proportions of up to 0.9 for 220508) or an increase in the time spent on exploratory and/or foraging dives (for 210427-A and 210427-B). There were eight non-feeding diel periods with TAS less than 0.6 for all other whales and regions, also owing to surface, exploratory and foraging time (supplementary material, figure S5).

**Figure 6.**
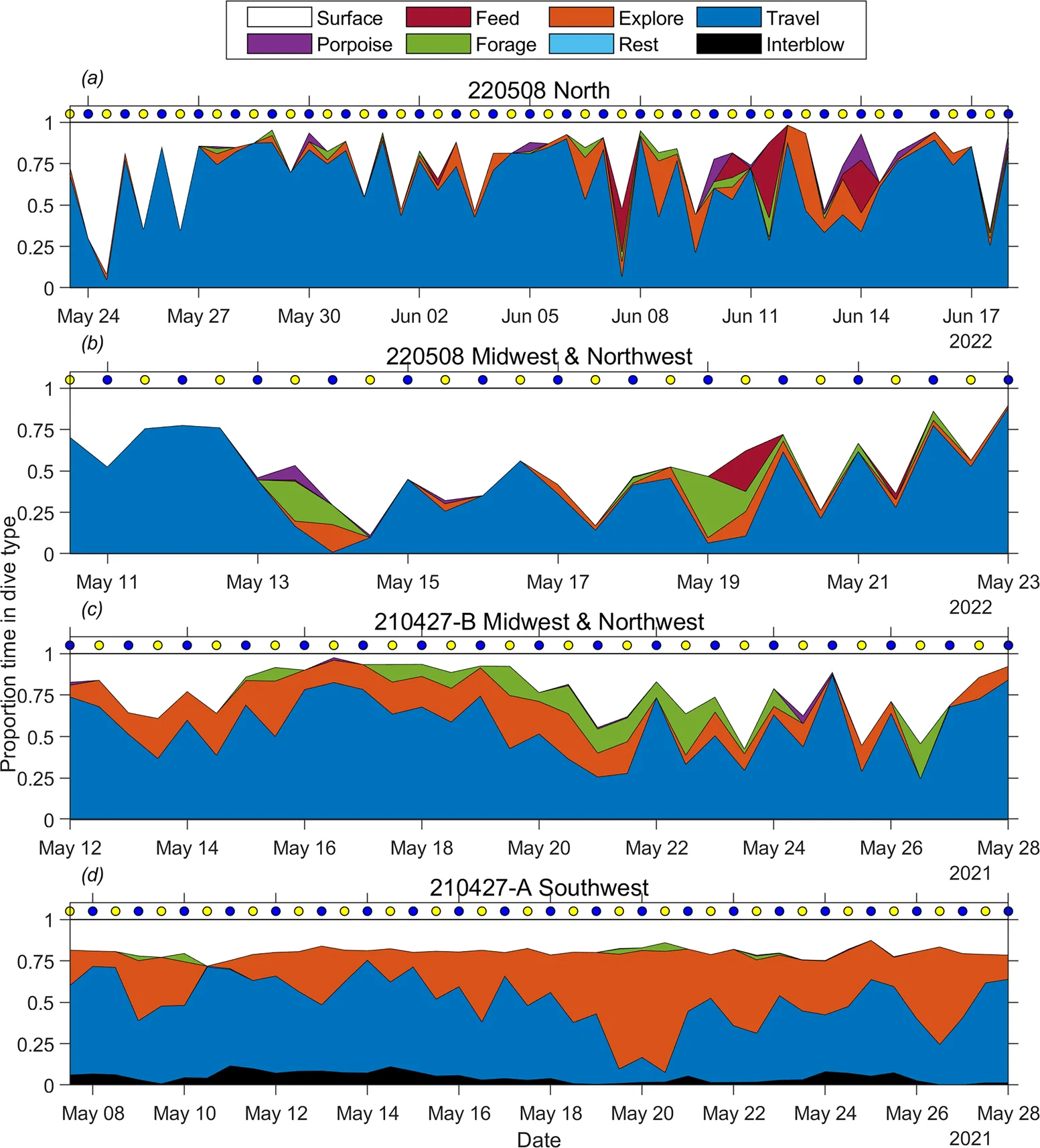
Time-series of dive behaviour as the proportion of time in each dive type and at the surface for three individual whales in four of the regions: whale 220508 in the North region (a), whale 220508 in the Midwest and North regions (b), whale 210427-B in the Midwest and North regions (c), and whale 210427-A in the Southwest region (d). TAS is only represented by travel type dives (blue), because rest type dives (cyan) were not observed in these subsets. The yellow and blue points indicate day and night periods. For whale 220508 in the North region, there was one period without a day/night indicator because there was no dive data for this diel period.

There was evidence of more surface time during the day than night in the Midwest, Northwest and North regions for 220508 and 210427-B (figure 6; supplementary material, figure S6). For 210427-B in the Midwest and Northwest regions, more time was also spent on foraging and exploratory dives during the day, but this was not the case for 220508 in these regions or for 210427-A in the Southwest. However, whale 220508 spent more time on exploratory dives in the North region during the day, but these were often adjacent to or within feeding periods.

### 3.4. Day–night singing ratio in passive acoustic monitoring data

Patterns of the day–night singing ratio varied across regions and migration directions. Perth Canyon had a very clear day–night trend of greater nocturnal singing activity, especially at the start of the season in March and April, which tapered to a gap in only the afternoon towards May (figure 7). By contrast, patterns in the Northwest region were not as strong and more temporally variable such as in dataset 12, which shows a strong nocturnal singing pattern from June to July (figure 7), but not during the peak singing in May (figure 8). Similarly, there was a clear offset between the peak of the singing rate (April and May) and the peak day–night singing ratio (February or March) for the Perth Canyon datasets (figure 8).

**Figure 7.**
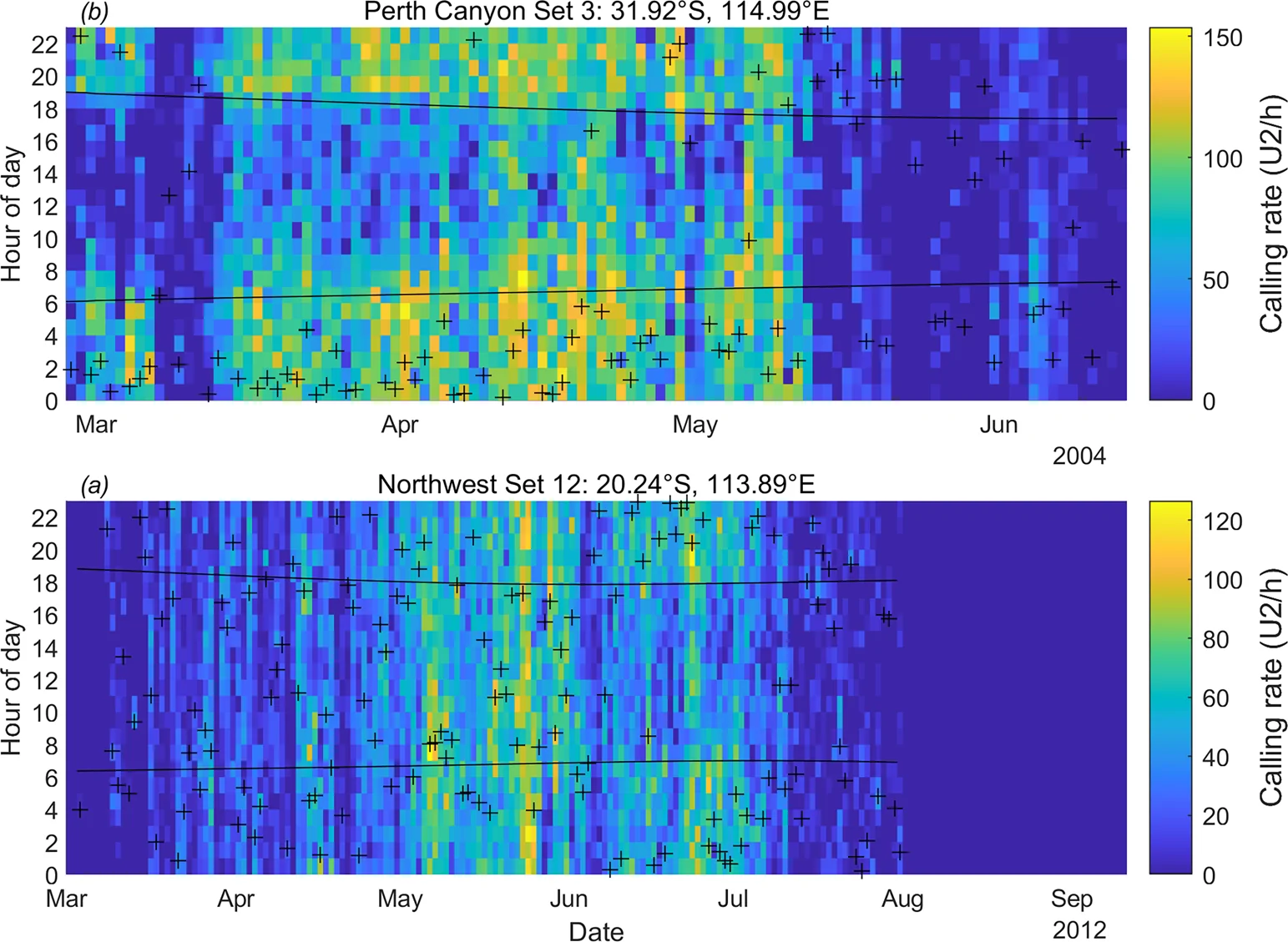
Periodicity of pygmy blue whale singing rate (unit 2 song calls per hour) by hour of day and date for two locations during the northbound foraging season: Perth Canyon in 2004 (*a*) and Northwest in 2012 (*b*). Black + symbols are the circular mean calling hour for each 24 h period, and the horizontal black lines indicate the time of sunset and sunrise for each day. Surface plots for each dataset analysed can be found in the supplementary material. Note that the colour scales are different between the axes to best distinguish within dataset periodicities rather than compare intensity between the sites.

**Figure 8.**
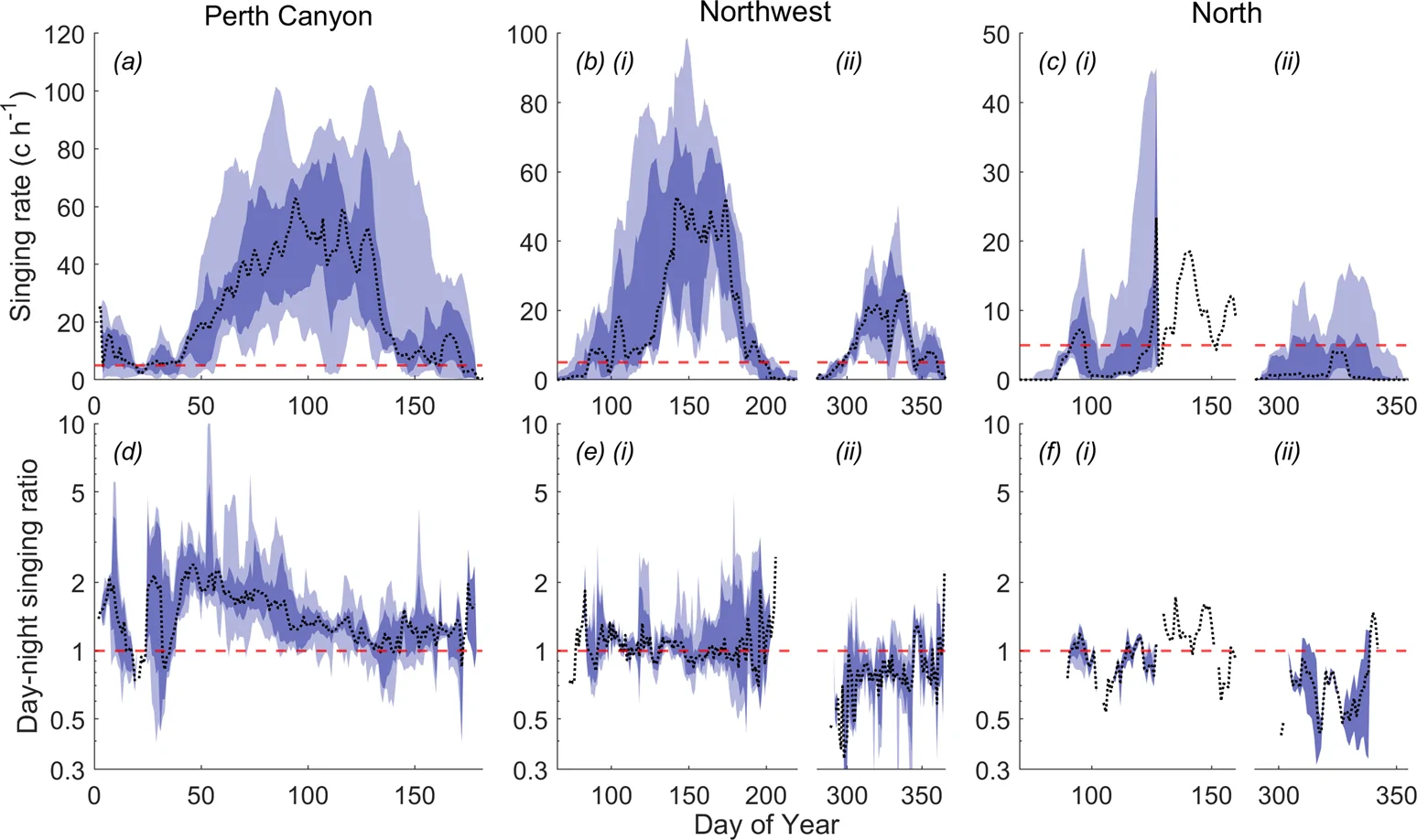
Seven-day moving average time-series of singing rate (*a–c*) and day–night singing ratio (*d–f*), with the median of datasets as the dotted line, dark shaded area represents the 25th to 75th percentiles, and light shaded area between the minimum and maximum. Columns separate datasets by regions, with breaks in the series during periods where singing rate is consistently below the threshold separating axes into the northbound (*i*) and southbound migrations (*ii*). Horizontal dashed red lines in the top plots are the singing rate threshold of 5 call h^−1^ for the day–night ratio to be calculated and in the bottom plots the threshold to identify nocturnal singing days.

The Perth Canyon had consistently stronger nocturnal singing activity during the northbound migration (ratio exceeded 1 on 75% of singing days) than in the Northwest (54%) and North regions (50%) (supplementary material, table S8). The southbound migration was only sampled from the Northwest and North regions, with smaller percentages of singing days having greater nocturnal activity (30 and 40%) as the median ratio was predominantly around 0.8 (figure 8). The Perth Canyon also had more nocturnal singing bouts than in the Northwest (excluding dataset 12) and North regions during the northbound migration (range 3–19 per dataset versus 3–9 and 1–3, respectively), with bouts lasting for longer durations (maximum ranging from 6 to 23 versus 2–9 and 2–6, respectively) and cumulatively accounting for a larger proportion of the nocturnal singing days (89 versus 43 and 47%, respectively) (supplementary material, table S8). While the characteristics of these bouts were consistent across datasets for the Perth Canyon, dataset 12 from the Northwest had more numerous (17) and longer bouts (maximum 17 days) that comprised a larger proportion of nocturnally active singing days (81%) than most datasets from this region.

## 4. Discussion

By pairing passive acoustic monitoring data with a mixed-effects modelling analysis of diving behaviour, we have demonstrated that greater EIOPBW singing at night versus day was related to daylight feeding behaviour. The proportion of TAS was low during diurnal feeding periods. In contrast, TAS was high during day and night non-feeding periods, as well as feeding-present nights. So, only daylight feeding would affect the diel periodicity of TAS. As all night-time behaviours had high TAS, a pattern of greater singing at night than day could be expected when feeding occurred. This pattern was consistent across the foraging regions of Perth Canyon, Midwest and Northwest. Therefore, the observable EIOPBW nocturnal singing prevalences in passive acoustic monitoring data collected in the Perth Canyon and Northwest regions were probably the result of foraging EIOPBWs. There was a reduced effect of feeding during the day on TAS in the North region where sample size was low and whales have been suggested to be focused on migration with intermittent opportunistic foraging [10]. Periods with a neutral day–night singing ratio may indicate migratory or non-feeding behaviour, which is supported by previous findings that a migrating EIOPBW sang marginally less at night than during the day [59]. In the northeast Pacific Ocean, migrating blue whales also sang less at night than during the day, while feeding whales sang more during the night [22,56]. The day–night patterns in feeding and singing appear to be transferable across the blue whale subspecies in these two ocean basins. Therefore, the day–night singing ratio could be applied as an indicator of EIOPBW feeding and migration just as it has been for the northeast Pacific Ocean blue whales [57,74]. Having a passively observable indicator of EIOPBW behaviour is crucial for understanding the areas important to this endangered species in the vast oceanic areas these whales inhabit [1]. This is especially the case given the large amount of passive acoustic monitoring data in these areas and the relative ease of collecting such datasets compared to biologging approaches.

Indicators of behaviour exist in audible communication not only for blue whales but also for vociferous animals across a wide range of taxa [75–77]. Linking bioacoustic signals to behavioural contexts can be challenging for species that are difficult to observe in marine, aquatic and terrestrial environments [75,78–80]. Pairing biologging and passive acoustic monitoring methods, as we have done here, enables bioacoustic methods to address a wide spectrum of research questions and management objectives [81–83]. Beyond these anthropic applications, conspecific listeners may also benefit from behavioural indicators in vocalizations [84,85]. Information about resources, threats and the environment transmitted through vocal communication are likely to assist the decision-making process of individuals [75,86]. This is particularly relevant to the movement and foraging strategies for species living in ecosystems where long-range communication is possible and resources are ephemerally available in dispersed, high-density patches [87,88], such as for cetaceans in pelagic ocean environments. For example, the blue whale day–night singing pattern may attract distant individuals to a foraging area or assist nearby individuals to decide if they should remain in the area [89,90].

### 4.1. Spatial and temporal considerations in the application of the day–night singing ratio as a behavioural indicator

Despite finding strong evidence to support the use of the day–night singing ratio as an indicator of behaviour, this was not the case in every region where feeding was observed. Daylight feeding in the North was not predicted to reduce TAS as it did for the Perth Canyon, Midwest and Northwest. This may be because EIOPBWs forage opportunistically in the North compared to intensively in the Perth Canyon and Midwest regions [10]. Blue whales in the northeast Pacific also demonstrated location-specific foraging plasticity [91–93], with more numerous and longer feeding bouts in near shore aggregations compared to fewer and more dispersed bouts in oceanic habitats [94]. Sex and individual-specific foraging strategies have also been detected in the northeast Pacific [94]. As the North region had just five feeding periods from one individual, the model predictions for this region were not likely to be representative of the whole population but rather of the individual's foraging strategy that was more opportunistic than elsewhere.

Satellite tracking data provide evidence that EIOPBWs adopt an opportunistic foraging strategy in the Northwest region compared to area-restricted foraging in the Perth Canyon and Midwest regions, based on lower residency times and faster travel rates [10]. These differences were hypothesized to reflect regional variation in prey availability, with prey being more ephemeral in the Northwest and more predictable in the Midwest and Perth Canyon [10]. Despite these differences in horizontal movement patterns, predicted TAS and feeding time per feeding-present day (and therefore foraging intensity) calculated for 12 h periods were similar among these three regions. Hourly feeding rates for blue and fin whales in the northeast Pacific have also been predicted to increase with the quality of prey patches, regardless of whether patches were aggregated or scattered in space or time [94–96]. These whales employed different strategies in response to the predictability of prey, with area-restricted foraging at near shore topographically forced upwelling centres and dispersed foraging in larger offshore areas with patchy productivity [94]. This is also likely to be the case for EIOPBW, where foraging strategies among regions may reflect differences in prey availability rather than the quality of prey resources encountered, as they forage with similar intensity when prey is available.

The temporal characteristics of observed singing periodicities in acoustic datasets reflected the different foraging strategies of EIOPBW in the Northwest compared to the Perth Canyon region. The Perth Canyon had nocturnal singing prevalences occurring in bouts of 1–4 weeks that spanned up to four months during the seasonal feeding aggregation [16], with peaks in February and March that coincided with increased subsurface productivity [97]. Bouts of greater nocturnal singing were less common in the Northwest acoustic datasets, with only one bout that had a total duration of approximately two months during winter. However, this Northwest bout was inconsistent in the day–night singing ratio, comprising many short nocturnal singing bouts each lasting a few days but no longer than one week (excluding one 17-day bout). These shorter bouts are probably a reflection of the opportunistic feeding strategy for EIOPBW during winter in the Northwest region [10]. Winter in the Northwest region is when feeding was observed in diving data [10], when productivity has been predicted to increase [98,99] and when the habitat was modelled to be most suitable [17]. Monthly singing intensity and day–night singing ratios also displayed site-specific and interannual trends in the northeast Pacific [100]. Such spatial and temporal patterns in singing behaviour may be a result of the diversity of dynamic prey fields across foraging areas [10,101–103], individual- or demographic-level foraging strategies [94], reproductive effort [40] and interannual environmental variation [104,105].

Variable temporal characteristics in day–night singing ratios resulting from different foraging strategies highlight that this behavioural indicator may be most effective when applied on a case-by-case basis. Specifically, the temporal scales used in the calculation of the day–night singing ratio should be optimized for site-specific foraging strategies. For example, Monterey Bay blue whale song periodicities transition from high nocturnal singing prevalence to a neutral pattern after the first few months of intense foraging, reflecting the population behavioural mode transition from foraging to migration [56,57]. This trend was present in the Perth Canyon passive acoustic monitoring datasets, while the peak day–night singing ratio was either before or after the overall singing peak in the Northwest region. Oestreich *et al.* [56] designed their analyses to detect a transition between behavioural modes by calculating the 15-day running mean of the day–night singing ratio and assessing the change in the mean over time. By contrast, many studies explore diel blue whale call periodicities over seasonal or yearly time periods to reveal long-term trends [41,46,106–108]. Combining trends from multiple months or years may inhibit the detection of patterns that may have occurred over shorter time periods owing to ephemeral, intermittent or opportunistic foraging.

While we have established that more singing during the night compared to the day could be an indicator of foraging, we have not explored the day–night singing ratio threshold at which foraging behaviour could be best distinguished from migration. The ratio is likely to vary with the number of singers (owing to density effects [38]), the proportion of singers foraging and migrating [100], the type of song being produced (which may vary with behaviour and location [39,59]) and even the size of the averaging window. These variables should be considered when determining the threshold to intersect day–night singing ratios for when most of the population is foraging versus migrating, rather than taking a value of 1 as we have done here. A neutral day–night singing ratio may be indicative that the population is migrating or transitioning between behaviour modes [57], or perhaps only that there was a mixture of migrating and foraging animals [100]. The latter is likely given that migrating individuals sang more during the day [56,59], and we observed this pattern particularly for the southbound migration at the soundscape level, where biologging data are too sparse to determine whether feeding occurs [17].

### 4.2. Drivers of daylight feeding preference

The cessation of feeding with the onset of night in all four foraging regions (with some exceptions) may indicate that there were common behavioural, physiological or ecological mechanisms driving this feeding periodicity. The deep feeding dives in the Midwest (400–600 m) and North regions (100–300 m) [10] suggest that the EIOPBWs may have been targeting vertically migrating euphausiid species that disperse to shallow waters at night, much like at the Perth Canyon [16]. Feeding was shallower (< 100 m) in the Northwest region on an unidentified prey, which may not indicate nocturnal dispersion. However, the most abundant krill species (*Pseudeuphausia latifrons* and *Stylocheiron carinatum*) at Ningaloo in the Northwest region were distributed in the top 100 m of the water column and are known to have short-distance vertical migrations [101,109,110]. Euphausiid physiology may contribute to daylight feeding preferences, as *Meganyctiphanes norvegica* in Oslofjord in Norway swam twice as fast at night compared to the day, once the water was oxygenated [111]. A prey that is more difficult to capture at night may disincentivize nocturnal feeding, especially if hunting techniques rely on visual cues.

Rolling behaviours at depth and aerial imagery of surface lunge-feeding indicate that vision may play a significant role in prey capture by blue whales [112,113]. However, daylight-exclusive feeding is not the rule for blue whales. There is a precedent for nocturnal blue whale feeding in the Gulf of St Lawrence [114], where shallower dives at night were more energy efficient owing to the reduced transit time to the surface [51]. A continuous feeding bout of 34.9 h has been reported from a blue whale in the northeast Pacific Ocean [94], as has night-time feeding with high interannual variability in lunge rates [56]. Audible sounds produced by euphausiids may assist blue whales in finding their prey at night [115] or at mesophotic depths. Rolling behaviours at depth may facilitate the auditory localization of prey, analogous to rotation during foraging echolocation by dolphins [116]. While not the dominant pattern, feeding and foraging dives at night have been observed for EIOPBW in the Northwest region [10], which may be a foraging strategy not well represented in our dataset owing to the small sample size. Physiological limitations might also influence foraging decisions. Whales may need periodic rest to metabolically focus on digestion, especially if the forestomach is full [117,118]. While there was great variation in feeding bout duration of blue whales in the northeast Pacific and eastern Indian Ocean, the medians were just 3.3 [94] and 4 h [10], respectively. The preference for diurnal feeding we have observed coincides with ecological and physiological incentives; however, there are evidently greater complexity and individual-level patterns that we do not yet understand.

### 4.3. Diel patterns in non-feeding behaviours

The application of the day–night singing ratio as an indicator of feeding behaviour assumes that non-feeding behaviours will result in more diurnal than nocturnal singing, as observed during migration [56,59]. However, in this study, some non-feeding behaviours reduced TAS during the day more than at night, including exploratory dives, foraging dives (in non-feeding periods) and extended time spent near the surface (< 1.5 m). Whether these behaviours were exclusive of singing probably depends on their function (i.e. why did the whale perform these behaviours for long periods of time), which will affect the interpretation of any TAS diel patterns arising from such non-feeding behaviours. Foraging dives were classified as such because they have similar depths to feeding dives but lack observations of prey-capture lunges, so probably represent an unsuccessful search for food [10]. Here, we observed diel periods with large proportions of foraging dives (often in concert with exploratory dives) in the absence of confirmed feeding, suggesting there may be other functions to these deep square-shaped dives. Some authors have hypothesized that deep V-shaped (i.e. exploratory) dives by blue whales and other cetaceans assist the localization of food resources [61,119–121]. Such food-searching behaviour has compatible function with feeding type dives and justifies their exclusion from the TAS. In diving vertebrates, V-shaped dives have also been proposed to increase swimming efficiency by reducing surface drag, to avoid predators and for collecting sensory input from the acoustic or physical environment to aid navigation [61,122–124]. The extensive time spent in exploratory or foraging dive types by EIOPBWs in the absence of feeding may have been to serve one of these alternate functions. Until greater insight about alternative functions of these exploratory and foraging dive types can be revealed, the assumption that these dives were related to food acquisition remains conservative for the analyses in this study.

Migrating whales spending extended time near the surface have not been widely recognized in the literature. Lewis *et al.* [22] reported greater surface time (shallower than 5 m) during daylight hours at the end of the feeding season compared to other months. Surface behaviour was correlated with down-sweep non-song vocalizations, especially for one individual [22], demonstrating that such surface behaviour was not prohibitive of sound production. This near-surface time may reflect a travelling depth preference. Travelling depths of EIOPBWs were 12.8 ± 5.3 m [10], coinciding with travelling depths reported elsewhere [21] and the optimal travelling depths to reduce surface drag as suggested by Owen *et al.* [61]. There may be some currently unknown benefit for travelling shallow particularly during the day. For example, thermoregulation has been a suggested function for surface swimming whale sharks [125], which might offset the energetic costs associated with surface drag. If the extended surface bouts represent travelling behaviour, excluding them from the TAS proxy may have underestimated the TAS in these cases. This may be particularly the case for the more northerly regions where greater time was spent shallower in non-feeding periods.

### 4.4. Model limitations

A notable limitation of the dataset was that the Perth Canyon and Southwest regions had a large proportion of data at 1 s sampling resolution, while Midwest, Northwest and North regions relied on 75 s sampling resolution data. At 75 s time-series resolution, we have less confidence in the identification of feeding dives compared to 1 s sample-rate data, especially for the shallow feeding in the Northwest region [10]. Even at 1 s sample rate, we are classifying three-dimensional movements based on only two dimensions, so there is uncertainty in all behavioural classifications (such as between foraging and exploratory dives). This uncertainty was accounted for by using the presence or absence of feeding dives as a factor in the model, so that individual classifications carried less weight. The most probable error to influence the model was the assignment of a feeding-present period owing to a false positive feeding dive, when all other dives in the period were not feeding. We reduced the probability of such an error by only classifying dives as feeding if there was agreement between two expert analysts.

Only male blue whales are known to sing [21,22,25], yet our dataset included diving behaviour from female whales (*n* = 5) and whales of unknown sex (*n* = 4). To address this limitation, we interpreted our results with the assumption that the whale's sex did not impact their motivation to prioritize feeding versus non-feeding dive behaviours, and all whales would have equal motivation to migrate. This may not be the case however, as male whales are likely to have more motivation to perform travel type dives than females, so that they can sing. As female whales also have a motivation to perform travel type dives to migrate and the sex ratio of EIOPBWs throughout their distribution is close to even [126], there is not enough evidence to reject our assumption of sex-independent motivation to conduct travel dives. A violin plot of TAS by sex, diel period and presence or absence of feeding revealed no obvious structure in the data owing to sex except in non-feeding contexts during the day (supplementary material, figure S7). This was likely because the whale that spent the most extended surface time was a female, so this whale may not have had any motivation to conduct travelling dives at depths optimal for singing. Meanwhile a singing whale will be more likely to travel at an optimal sound production depth to most effectively broadcast their song [127,128], which was suggested to be between 10 and 40 m based on empirical measurements [22,59].

## 5. Conclusion

This work has shown that greater EIOPBW singing during the night compared to the day is likely to be an indicator of population-level feeding in the Perth Canyon, Midwest and Northwest regions. This day–night singing ratio when feeding contrasts with the expected pattern when in migratory or non-feeding behaviours, which would be less or equal singing at night compared to the day. This study is in lieu of expensive site-specific field expeditions that could validate these conclusions with paired acoustic and movement biologging data, which could quantify individual-level singing rates as a function of behaviour, location, diel period and sex. This research enables future work to assess trends in day–night singing ratios in passive acoustic monitoring datasets in the Perth Canyon, Midwest and Northwest regions and explore the interannual variation in foraging effort as a function of oceanographic or environmental drivers. Care needs to be taken if applying a static threshold to infer foraging based on the day–night singing ratio, as we have done here. To inform thresholds at which the day–night singing ratio can best distinguish migration from foraging behaviour, future work should determine singing rates under a variety of scenarios across a robust sample size capable of making population-level inference. For other feeding areas, such as in the seas of Southeast Asia, additional biologging data are required to validate day–night singing ratios as an indicator of behaviour. More work is required to understand the prevalence of sex-specific diving behaviours and diel patterns in non-feeding behaviours.

## Supplementary Material



## Data Availability

The datasets and code supporting this article have been uploaded as part of the supplementary material [129].
