# Supplementary material for: Pygmy blue whale feeding drives greater nocturnal than diurnal song production
Source: R Soc Open Sci. 2026 Oct 28;13(10):260954. doi: 10.1098/rsos.260954 (PMC13714375; doi:10.1098/rsos.260954)
Supplement: Supplementary file 1 [file rsos.260954.zip › rsos.260954/rsos260954_si_001.pdf]

Pygmy blue whale feeding drives greater nocturnal than diurnal song production

Andrew M. Davenport, Michele Thums, K. Curt S. Jenner, Micheline-Nicole M. Jenner, Luciana C. Ferreira, Christine Erbe, Ryan Leadbetter, Robert D. McCauley

Royal Society Open Science

DOI: 10.1098/rsos.260954

Supplementary Materials: dive processing

Contents

Square root dives 2

Combination dives 3

Deep feeding with Activity time series 5

Deep feeding without Activity time series 6

Surface feeding 7

Moving the boundaries of dives to the surface 9

References 10

Square root dives

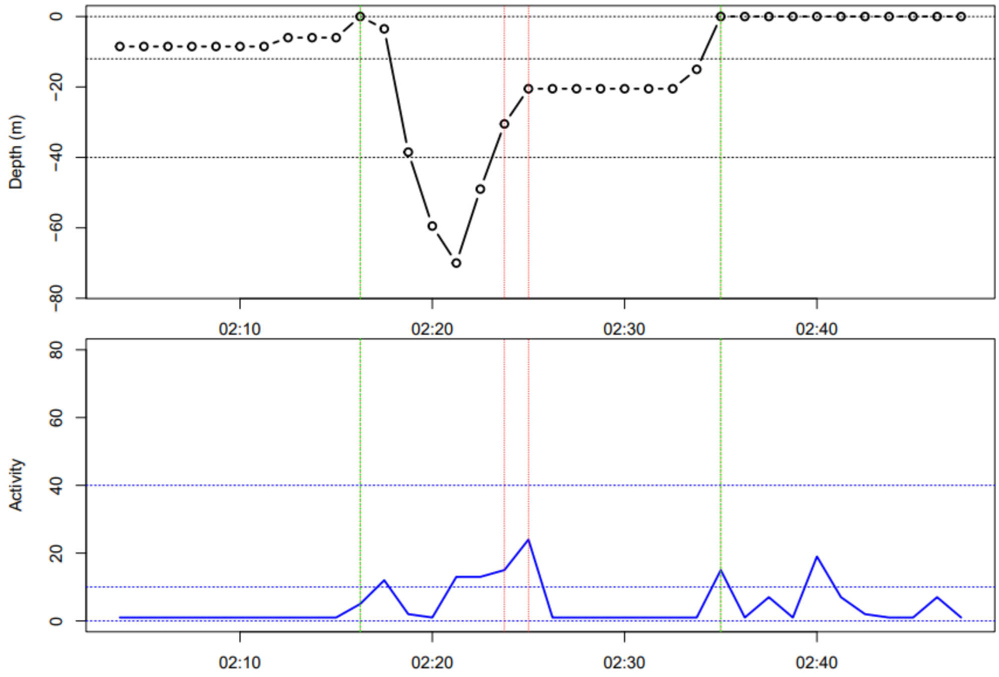

Figure 1. A square root dive from whale 220508 in the Northwest Region, notice the two parts, first the exploratory section (diving quickly to –70 m around 02:20 and ascending again) followed by a travelling section (at –25 m from 02:25 to 02:35). Green vertical lines are the original dive boundaries, and the red lines indicate the end and start points manually delineated for the two dive parts. These are on separate points so that the calculation of dive duration is not overestimated. Black circles indicate the depth samples, one every 75 s. The time axis is in hh:mm universal time continuum.

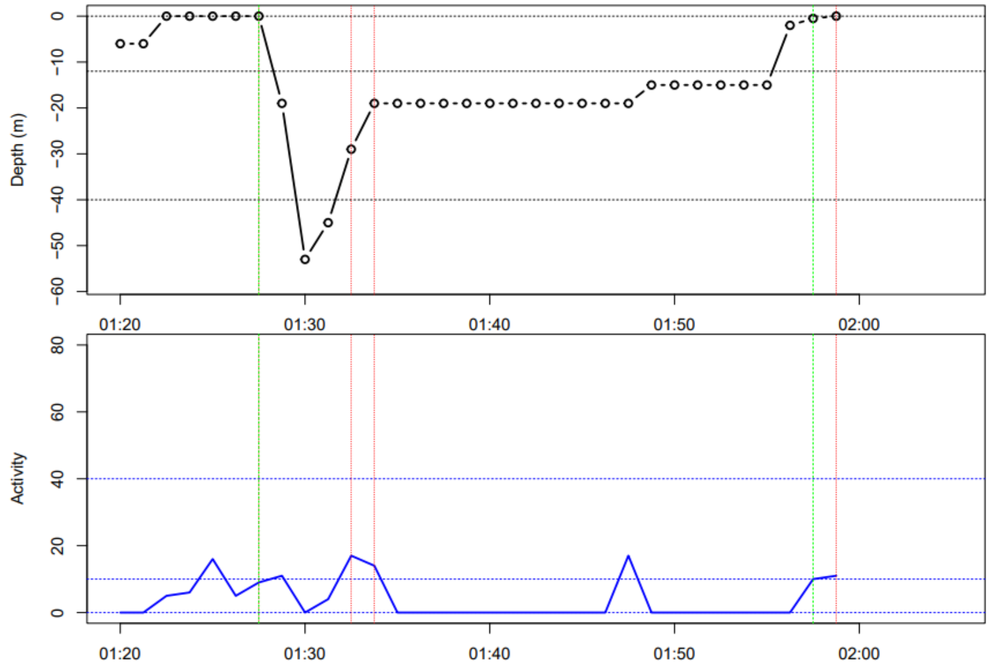

Figure 2. A square root dive from whale 220508 in the Northwest Region, notice the two parts, first the exploratory section (diving quickly to –55 m around 01:30 and ascending again) followed by a travelling section (at –20 m from 01:34 to 01:58). Green vertical lines are the dive boundaries set by the R [1] package divemove [2] in Thums et al. [3] (see methods section of the parent manuscript)*, and the red lines indicate the manually adjusted boundaries of the two parts of the dive. The red line farthest to the right is where the end point of the dive was adjusted to be at depth = 0 m.*

Combination dives

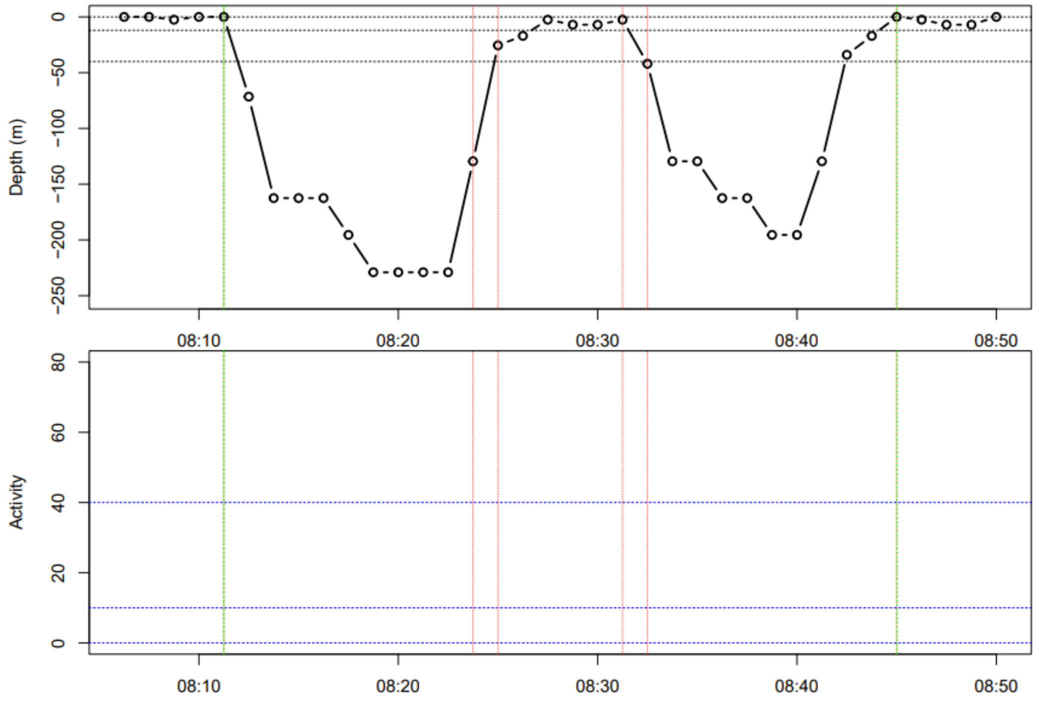

Figure 3. A dive from whale 220508 classified as "other" in Thums et al. [3]. Here, we refer to such a dive as a "combination dive" as it has multiple parts representing distinct behaviours. This dive was split into three dive parts: foraging (at –160 to –240 m depth from 8:12 to 8:22), travelling (at –20 m depth from 8:25 to 8:31), and foraging (down to –200 m depth from 8:33 to 8:40). Green vertical lines are the original dive boundaries, and the red lines indicate the new boundaries.

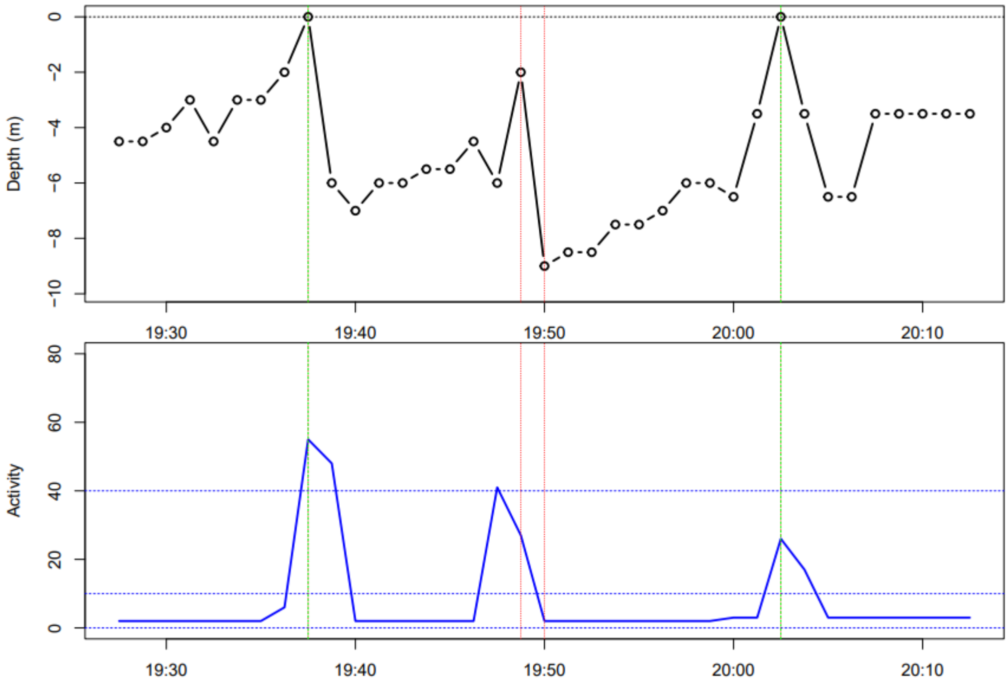

Figure 4. A dive from whale 220508 considered here to be a "combination dive" and classified as "other" in Thums et al. [3]. This dive was split into two travelling dive parts as it is likely there was a surfacing separating them, as evidenced by the spike in depth and Activity (at about 19:49) as the tag breaks the surface. Green vertical lines are the original dive boundaries, and the red lines indicate the new boundaries.

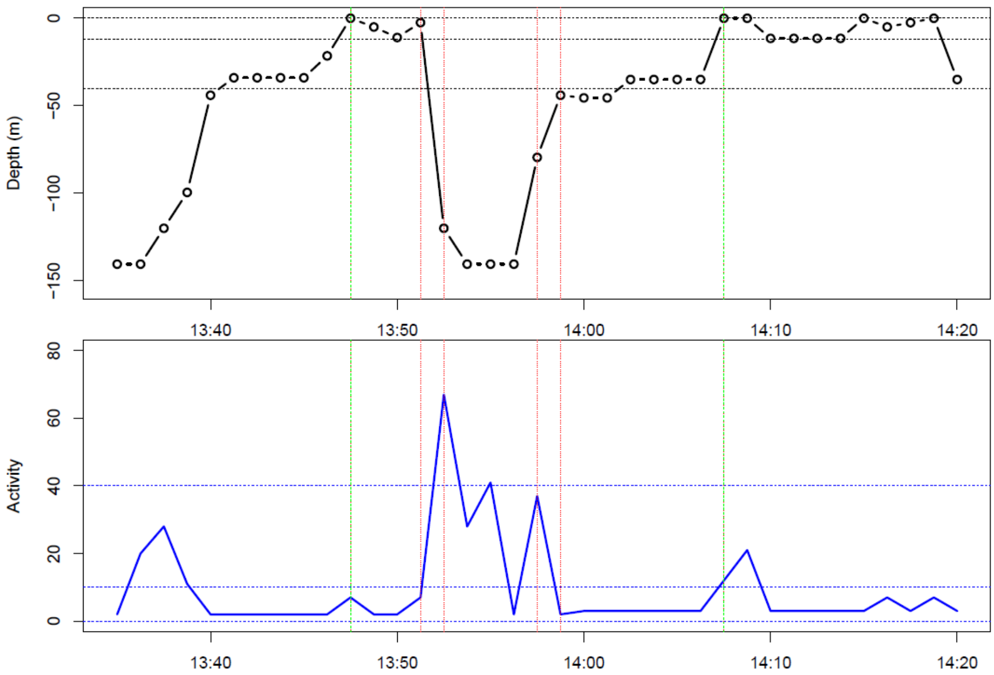

Figure 5. A dive from whale 230523 classified as "feeding" in Thums et al. [3], which was split into three parts: travelling (at –20 m depth centred around 13:50), feeding (down to –150 m depth centred around 13:55), and travelling again (at –40 m depth from 13:59 to 14:08). The feeding classification was given because of the spikiness in Activity, which is assumed to be due to feeding lunges and processing times even in the absence of depth wiggles. Green vertical lines are the original dive boundaries, and the red lines indicate the new boundaries.

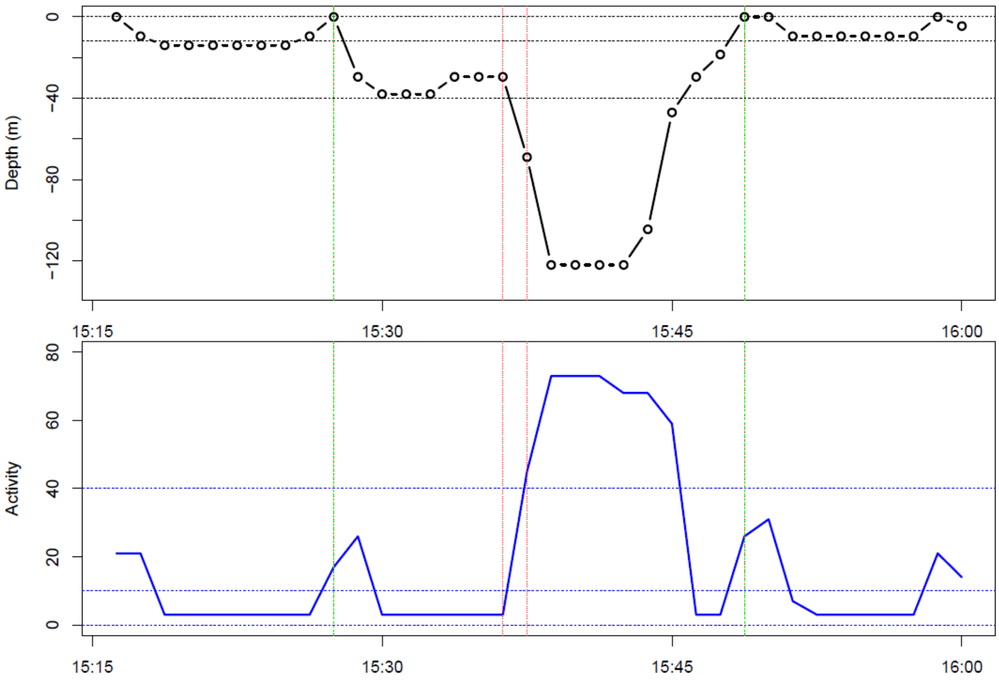

Figure 6. A dive from whale 230523 classified as "foraging" in Thums et al. [3], which was split into two parts, the first being travelling (at –40 m depth from 15:29 to 15:37) and the second being foraging (down to –120 m depth from 15:38 to 15:44). Notice that the Activity is not spikey, hence why it was classified as foraging not feeding (because an Activity value near 75 indicates there was not a break in energetic movement that could be associated with food processing). Green vertical lines are the original dive boundaries, and the red lines indicate the new boundaries.

Deep feeding with Activity time series

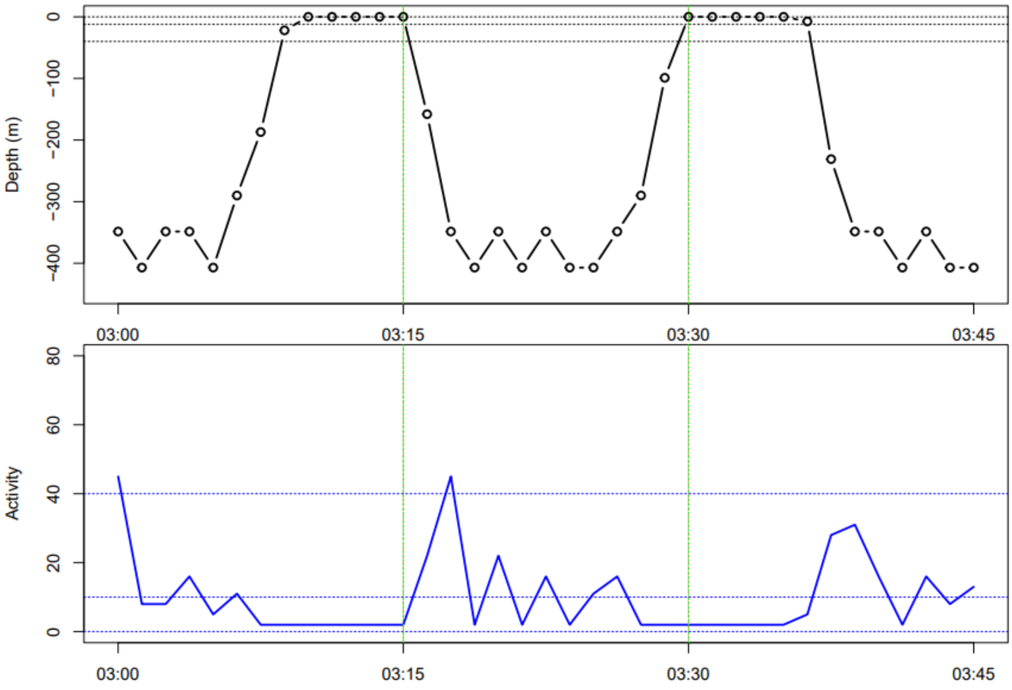

Figure 7. A feeding dive from whale 220507. One can clearly see the spikes in depth correspond to the spikes in the Activity time series, and there are clear pauses in Activity that align with the times that the whale is depressing the ventral pleats and processing food.

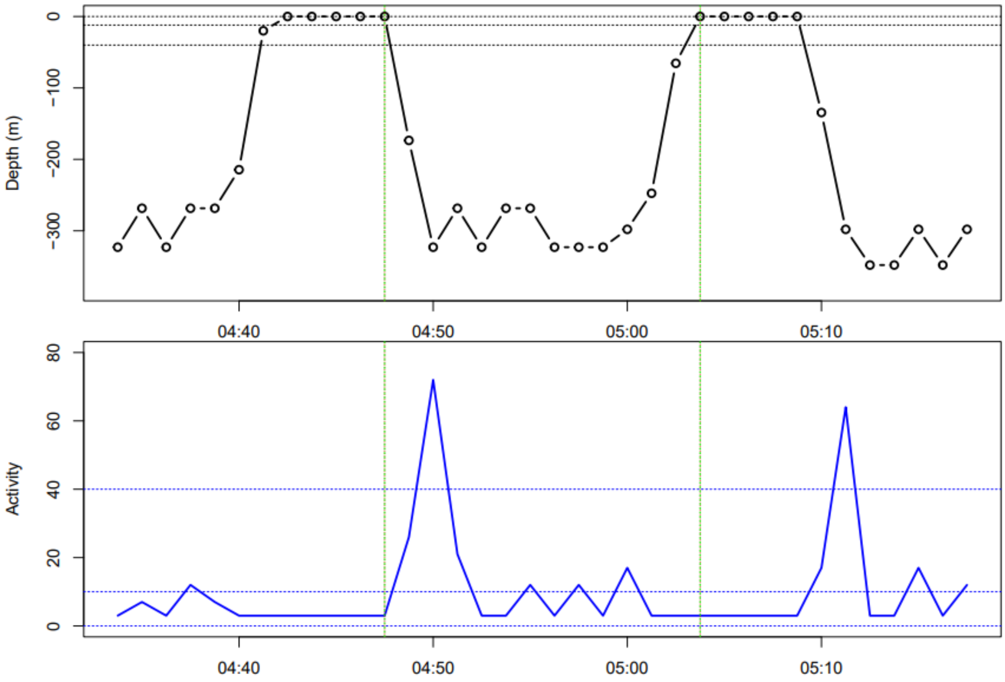

Figure 8. A feeding dive from whale 220507. In contrast to the above figure, here the feeding yo-yos and spikes/troughs in Activity at the beginning of the dive are out of phase with the tag transmitted sampling rate. Towards the end of the dive, one can see the Activity spikes remain, but the depth yo-yos are no longer present, likely because they became in phase with the sample rate (i.e., one whole lunge yoyo cycle occurred in 75 s, so the depth at the start and end of that cycle was the same). However, we can still capture the lunges on the Activity time-series, which summarises the high-Activity events over the past 75 s as opposed to being a snapshot of the instantaneous acceleration.

Deep feeding without Activity time series

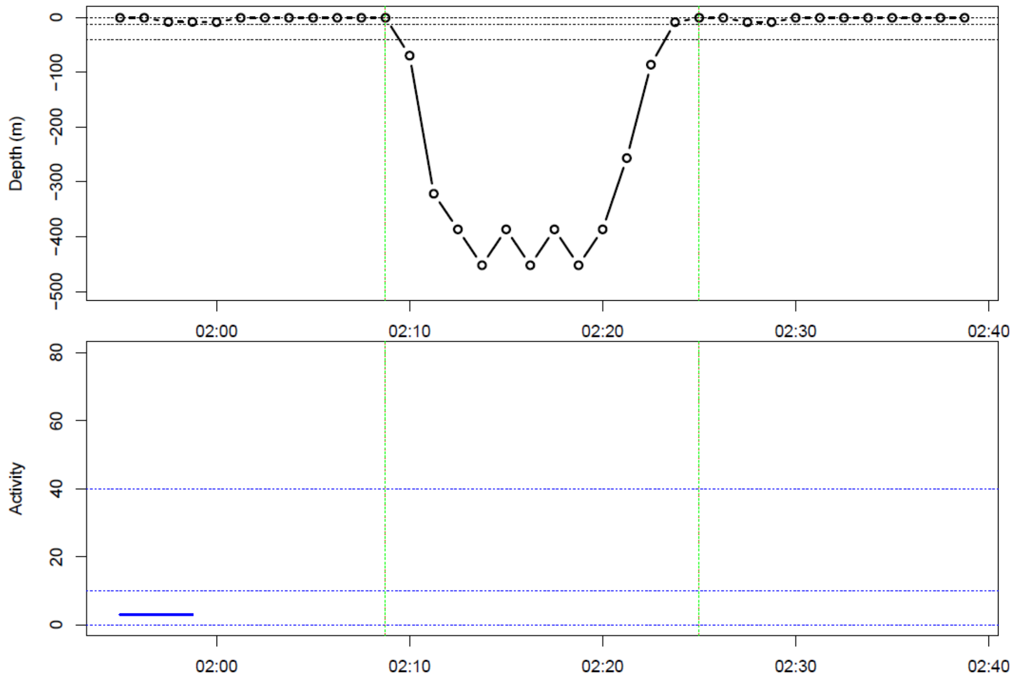

Figure 9. A feeding dive demonstrated by whale 220507 amongst the Abrolhos Canyons. The lack of the Activity time-series data inhibits 100% confidence in assigning this as a feeding dive but given the depth and the yo-yo depth pattern on the bottom supports assigning it as a likely feeding dive.

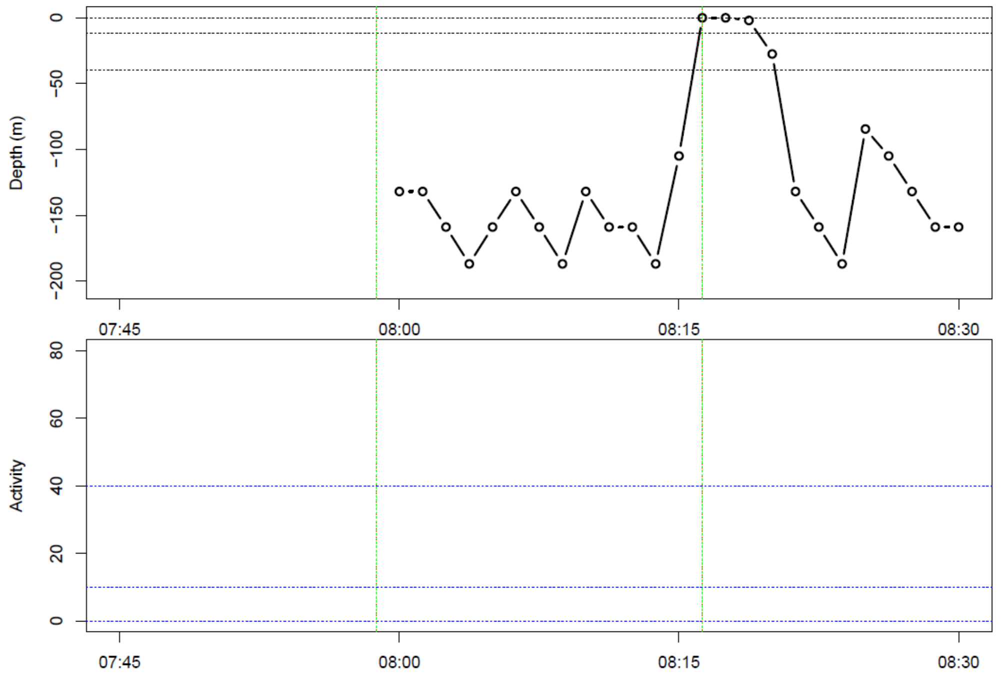

Figure 10. An incomplete feeding dive by whale 220508 in the Savu Sea.

Surface feeding

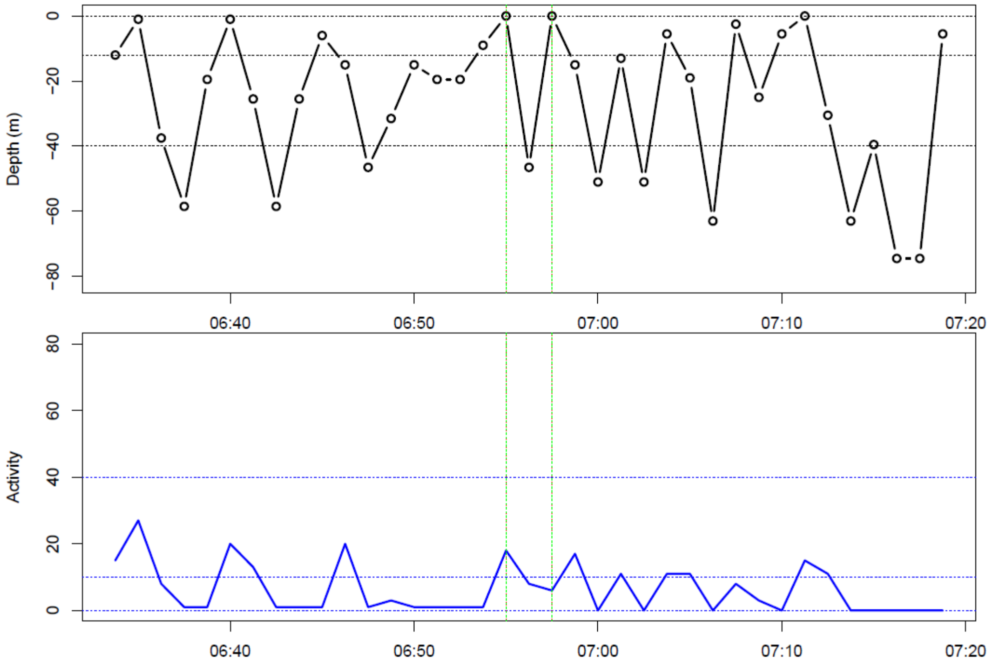

Figure 11. A bout of possible surface feeding displayed by whale 230523 in the Northwest region. Notice that the Activity time-series is often low, and spikes at or just after the peaks in depth/ surfacings. These rapid excursions to depths ~–50 m and back to the surface (like a yo-yo) are similar in magnitude and duration to feeding depth wiggles on deep feeding dives.

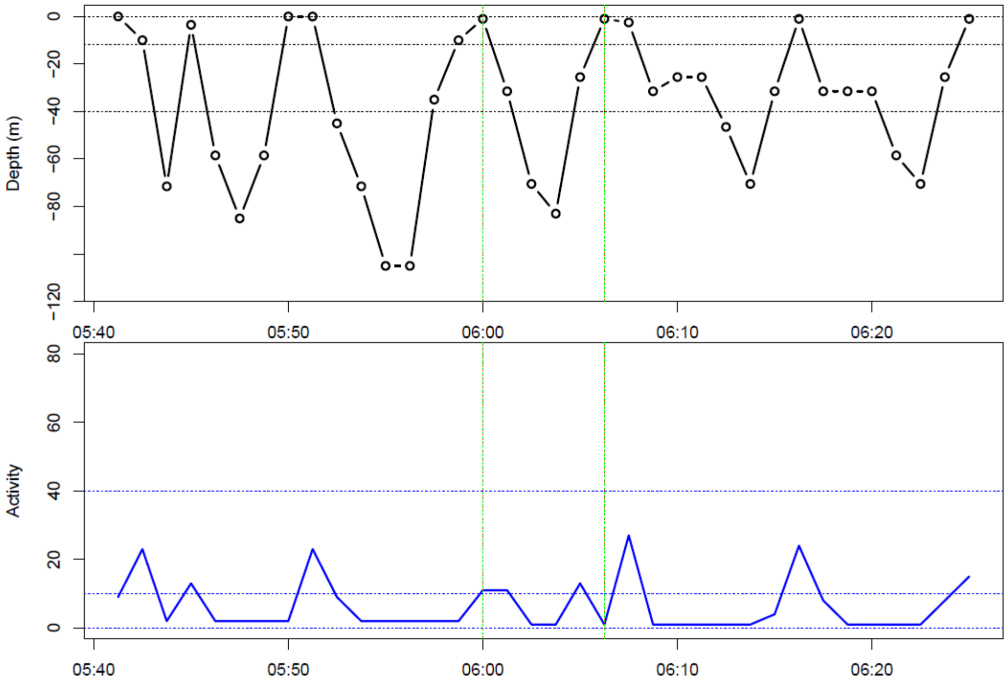

Figure 12. Another bout of possible surface feeding dives by whale 230523 in the Northwest region, set immediately prior to the previous figure. Note that the cadence of the yo-yo profiles is lower.

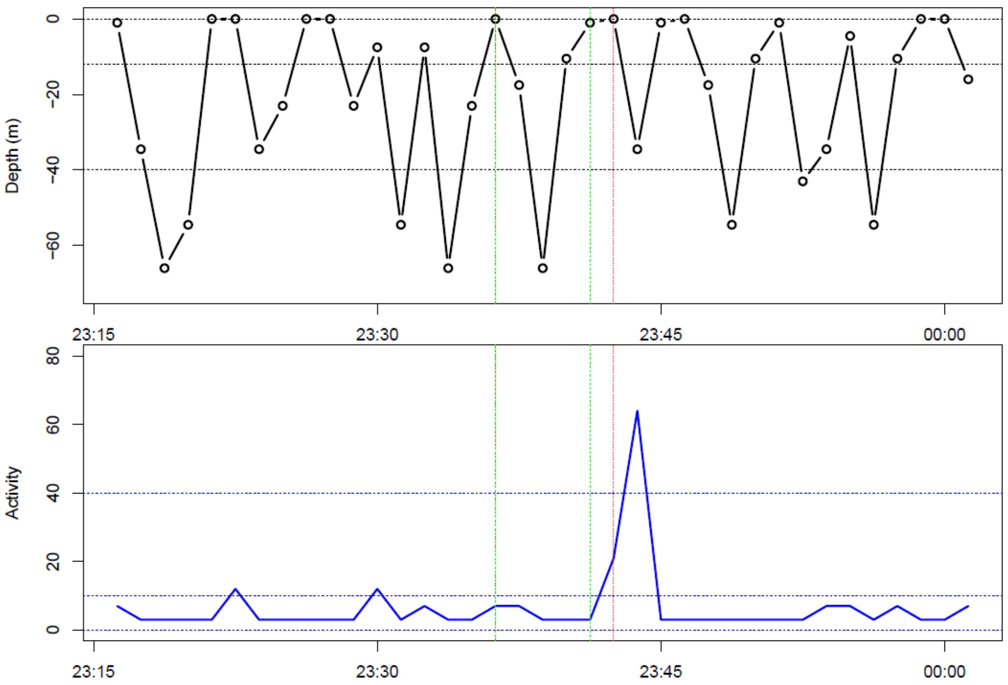

Figure 13. Another temporally independent bout of surface feeding by whale 230523 in the Northwest region (4 days later), with similar cadence and depth as the first example.

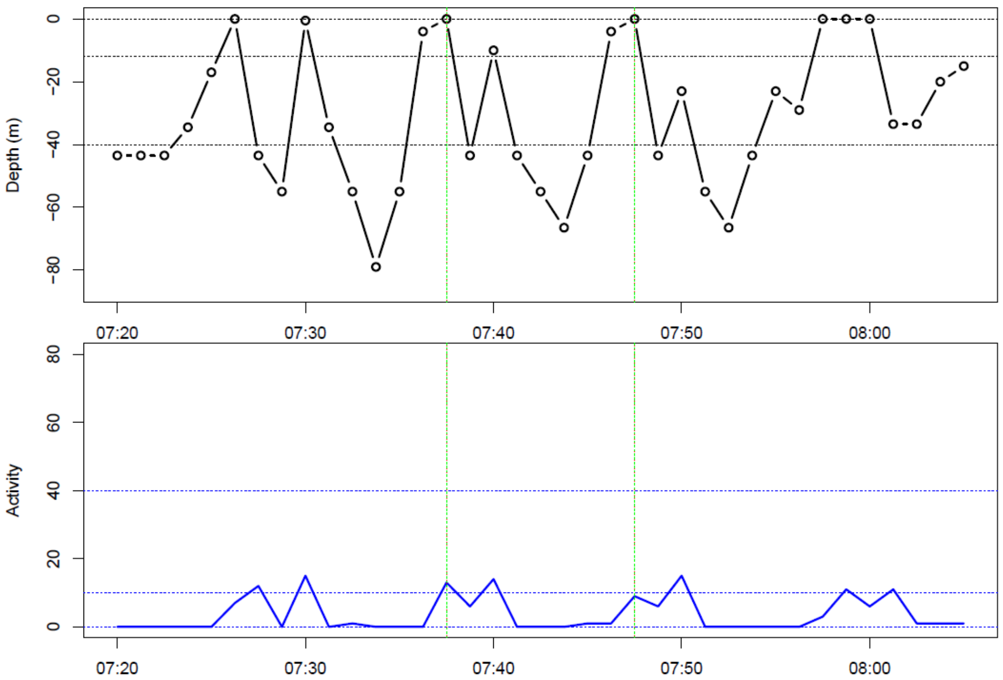

Figure 14. A bout of possible surface feeding from whale 220508 in the Northwest region.

Moving the boundaries of dives to the surface

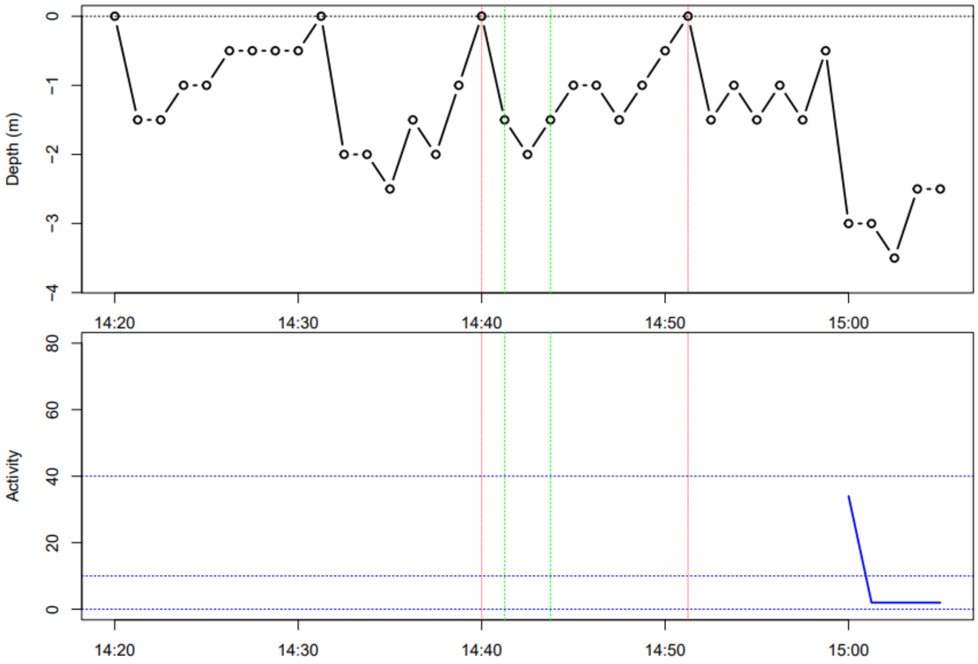

Figure 15. A dive classified as “too short” in Thums et al. [3], because there was only one data point in the dive (constrained by the green vertical lines). By first moving the start and end points to the nearest surfacing (red vertical lines), this dive had more information to be considered and was classified as “travel”. The borders of the dive to the surface also resulted in merging two “too short” dives, the second being after the depth peak.

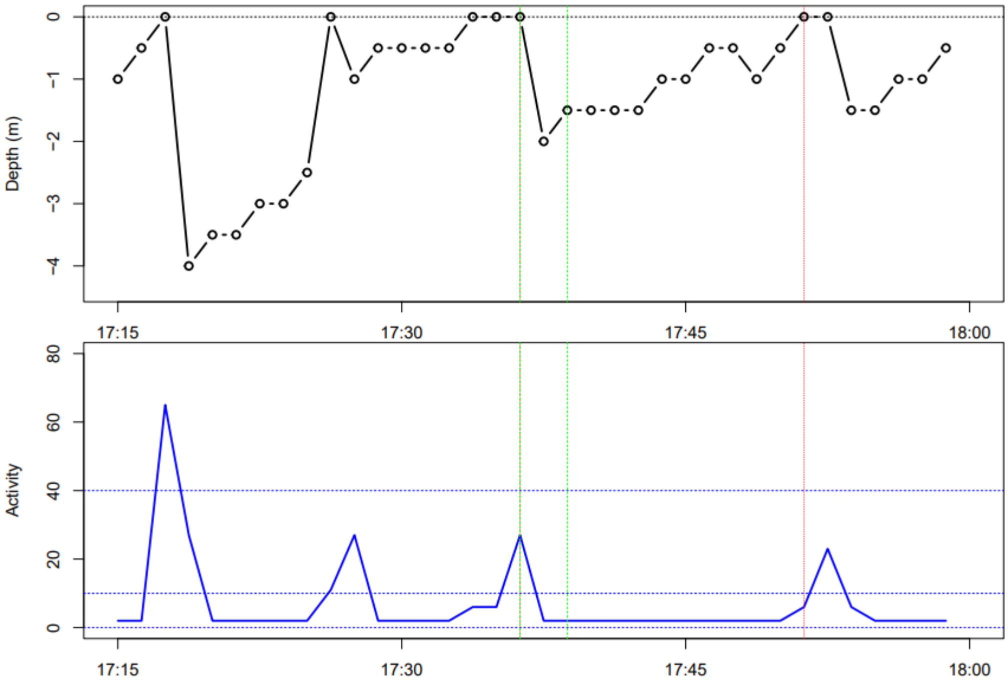

Figure 16. Similarly to the dive changes in Figure 17, the right border of this dive was moved to the nearest surfacing to include an extra 10 minutes of dive time. This change is supported by the Activity time-series, which only shows a peak (an indicator of the tag breaking the surface) when the depth record reaches zero, and not at depth = –0.5 m. It is possible that this whale was resting with part of its back above water, but the part with the tag attached was still submerged. We do not have whale orientation here, so it may be that the whale was rolling around at the surface and the depth changes are due to rotation rather than a change of position in the water column.

References

1. R Core Team. 2022 *R: A language and environment for statistical computing*. Vienna, Australia: R Foundation for Statistical Computing.

2. Luque SP, Guinet C. 2007 A maximum likelihood approach for identifying dive bouts improves accuracy, precision and objectivity. *Behaviour* **144**, 1315–1332. (doi:10.1163/156853907782418213)

3. Thums M, Ferreira LC, Davenport A, Jenner M, Moller L, Russell G, McCauley RD, Jenner C. 2025 Tracking pygmy blue whale diving behaviour and validation of foraging defined from horizontal movement data. *Glob. Ecol. Conserv.* **57**, e03362. (doi:10.1016/j.gecco.2024.e03362)
