# Supplementary material for: Pygmy blue whale feeding drives greater nocturnal than diurnal song production
Source: R Soc Open Sci. 2026 Oct 28;13(10):260954. doi: 10.1098/rsos.260954 (PMC13714375; doi:10.1098/rsos.260954)
Supplement: Supplementary file 1 [file rsos.260954.zip › rsos.260954/rsos260954_si_002.pdf]

Pygmy blue whale feeding drives greater nocturnal than diurnal song production

Andrew M. Davenport, Michele Thums, K. Curt S. Jenner, Micheline-Nicole M. Jenner, Luciana C. Ferreira, Christine Erbe, Ryan Leadbetter, Robert D. McCauley

Royal Society Open Science

DOI: 10.1098/rsos.260954

Supplementary methods and results

Methods

Passive acoustic monitoring (PAM) datasets were investigated for daily periodicities in EIO pygmy blue whale singing, to justify that the hypothesised foraging-induced day-night singing ratios empirically exist in PAM data. Datasets were used from the North (15), Northwest (11) and Perth Canyon (10) regions (Table 1). The second unit (U2) of the EIO pygmy blue whale song was detected in each dataset using an automated algorithm targeting the third harmonic (McCauley et al. 2018). Manual validation was conducted on each dataset. Datasets were merged and separated into seasons/ migrations to improve comparability. Perth Canyon datasets were separated into the feeding season (January–July) and the off season (August–December), keeping years separate, but merging data that fell into the same season of the same year. The Northwest and North recordings were separated into north (March–August) and south migrations (September–February), so that a continuous deployment resulted in having different set numbers for the different migrations.

Table S1. Metadata of all the passive acoustic monitoring stations and their visually observed pattern. MigD is the migration direction where N is north and S is south. Water depth is calculated from the ETOPO 60 arc-second global bathymetry and not from the in-situ observations.

Set #	Region	MigD	Latitude	Longitude	Start	End	Duration (d)	Sample length (s)	Interval (min)	Water depth (m)
**1**	Perth Canyon	N	-31.92	114.99	2000-Mar-08	2000-Apr-10	33.0	90	10	480
**2**	Perth Canyon	N	-31.92	114.99	2003-Feb-18	2003-Jun-07	109.1	205	15	480
**3**	Perth Canyon	N	-31.92	114.99	2004-Feb-27	2004-Jun-14	108.6	205	15	480
**4**	Perth Canyon	N	-31.92	114.99	2004-Dec-30	2005-Jul-08	190.3	205	15	480
**5**	Perth Canyon	N	-31.92	114.99	2007-Jan-01	2007-Apr-25	113.4	205	15	480
**6**	Perth Canyon	N	-31.92	114.99	2008-Feb-26	2008-Apr-21	54.7	205	15	480
**7**	Perth Canyon	N	-31.92	114.99	2009-Feb-25	2009-Jul-01	126.1	512	15	480
**8**	Perth Canyon	N	-31.92	114.99	2010-Jan-01	2010-Jul-01	181.0	460	15	480
**9**	Perth Canyon	N	-31.92	114.99	2011-Jan-01	2011-May-08	127.2	410	15	480
**10**	Perth Canyon	N	-31.92	114.99	2014-Jan-01	2014-Jul-01	181.0	307	15	480
**11**	Northwest	S	-20.24	113.89	2011-Oct-07	2012-Mar-01	145.9	512	15	1070
**12**	Northwest	N	-20.24	113.89	2012-Mar-01	2012-Sep-11	194.3	256	15	1070
**13**	Northwest	N	-20.39	114.07	2012-Mar-01	2012-Sep-11	194.2	256	15	1161
**14**	Northwest	S	-19.39	115.91	2012-Nov-20	2013-Mar-01	100.6	307	15	226
**15**	Northwest	N	-19.39	115.91	2013-Mar-01	2013-Sep-01	184.0	307	15	226
**16**	Northwest	S	-19.38	115.93	2014-Aug-11	2015-Mar-01	201.5	307	15	223
**17**	Northwest	N	-19.38	115.93	2015-Mar-01	2015-Jun-13	104.3	307	15	223
**18**	Northwest	N	-21.76	113.88	2018-May-16	2018-Sep-01	107.6	307	15	252
**19**	Northwest	S	-21.76	113.88	2018-Sep-01	2019-Mar-01	181.0	307	15	252
**20**	Northwest	N	-21.75	113.88	2019-Mar-01	2019-Sep-01	184.0	307	15	255
**21**	Northwest	S	-21.75	113.88	2019-Sep-01	2020-Feb-21	173.0	307	15	255
**22**	North	S	-18.08	120.4	2017-Sep-12	2018-Mar-01	170.0	3275	60	102
**23**	North	S	-17.45	119.95	2017-Sep-11	2018-Mar-01	170.6	3418	60	302
**24**	North	N	-17.45	119.95	2018-Mar-01	2018-May-09	69.3	3418	60	302
**25**	North	S	-17.11	119.82	2017-Sep-11	2018-Mar-01	170.8	3596	60	398
**26**	North	N	-17.11	119.82	2018-Mar-01	2018-Jun-14	105.1	3596	60	398

**27**	North	S	-16.77	119.69	2017-Sep-11	2018-Mar-01	170.9	3600	60	509
**28**	North	N	-16.77	119.69	2018-Mar-01	2018-May-10	69.9	3600	60	509
**29**	North	S	-16.48	119.49	2017-Sep-10	2018-Mar-01	171.5	2986	60	856
**30**	North	N	-16.48	119.49	2018-Mar-01	2018-May-10	70.0	2986	60	856
**31**	North	S	-16.2	119.28	2017-Sep-10	2018-Mar-01	171.6	3195	60	1538
**32**	North	N	-16.2	119.28	2018-Mar-01	2018-May-10	70.1	3195	60	1538
**33**	North	S	-15.91	119.08	2017-Sep-10	2018-Mar-01	171.8	3336	60	2335
**34**	North	N	-15.91	119.08	2018-Mar-01	2018-May-10	70.2	3336	60	2335
**35**	North	S	-15.48	118.77	2017-Sep-09	2018-Mar-01	172.7	3385	60	4165
**36**	North	N	-15.48	118.77	2018-Mar-01	2018-May-11	70.8	3385	60	4165

Results

Table S2. Model selection process for the full dataset. D = diel period, R = region, F = feeding, W = whale. Fixed refers to the fixed covariate function. Random refers to the random covariate function, where the random effect W is applied to the intercept (1|W), or a fixed covariate (e.g., R|W). μ refers to the mean model and Φ to the precision model. is the expansion of individual and interaction terms for the relevant factors.*

ID	df	AIC	μ fixed	μ random	Φ fixed	Φ random
M0	12	-238.6893	D*R+F			
M0a	Did not converge		D*R*F			
M0b	8	-236.9641	D+R+F			
M0c	Did not converge		D+R*F			
M0d	9	-292.8151	D*F+R			
M0e	11	-133.5675	D*R			
M0f	5	-255.9793	D*F			
M0g	Did not converge		R*F			
M0h	7	-102.0497	D+R			
M0i	4	-207.8235	D+F			
M0j	7	-217.5737	R+F			
M0k	3	-77.4198	D			
M0l	6	-30.00473	R			
M0m	3	-188.0445	F			
M0n	13	-291.1701	D*F+R*D			
M0d selected						
M0d	9	-292.8151	D*F+R			
M1a	10	-291.5988	D*F+R		F	
M1b	10	-301.6509	D*F+R		D	
M1c	13	-308.2772	D*F+R		R	
M1c selected						
M2a	14	-306.8623	D*F+R		R+F	
M2b	14	-318.6276	D*F+R		D+R	
M2b selected						
M3a	15	-323.1883	D*F+R		D+R+F	
M3b	18	-313.184	D*F+R		D*R	
M3a selected						
M4a	Did not converge		D*F+R		D+R*F	
M4b	19	-319.9106	D*F+R		D*R+F	
M4c	16	-330.6736	D*F+R		D*F+R	
M4c selected						
M5a	20	-326.1898	D*F+R		D*F+R*D	
M4c selected						
M4c	16	-330.6736	D*F+R		D*F+R	
M5b	17	-409.7161	D*F+R	1|W	D*F+R	
M5c	17	-342.6585	D*F+R		D*F+R	1|W
M5b selected						
M6a	18	-415.8312	D*F+R	1|W	D*F+R	1|W
M6b	False convergence		D*F+R	R|W	D*F+R	
M6c	19	-411.5561	D*F+R	D|W	D*F+R	
M6d	19	-414.332	D*F+R	F|W	D*F+R	
M6a selected						
M7a	20	-423.3715	D*F+R	D|W	D*F+R	1|W
M7b	20	-420.0301	D*F+R	F|W	D*F+R	1|W
M7c	Did not converge		D*F+R	R|W	D*F+R	1|W
M7d	Singular convergence		D*F+R	1|W	D*F+R	D|W
M7e	Singular convergence		D*F+R	1|W	D*F+R	F|W
M7f	False convergence		D*F+R	1|W	D*F+R	R|W
M7a selected						
M8a	22	-423.7207	D*F+R	D|W	D*F+R	D|W
M8b	22	-428.5196	D*F+R	D|W	D*F+R	F|W
M8c	Singular convergence		D*F+R	D|W	D*F+R	R|W
M8b selected as best overall model						

Table S3. Statistical output from the final model for the full dataset (M8b), in the top left cell has the model specifications (TAS = time available for singing, D = diel period, F = presence/absence of feeding, R = region, W = whale). The central cell

has the parameters for the fixed effects part of the mean model, the random effects are left centre. The right cell shows the parameters for the fixed effects of the precision model, the random effects are left bottom. Significant p-values are in bold.

**Final model - Family: beta (logit)**			
*Mean expression: TAS ~ D * F + R + (D|W)* *Precision expression: ~ D * F + R + (F|W)*			
**Random effects: mean parameter**			
Name	*Variance*	*Std.Dev.*	Corr
Intercept	0.693	0.833	
D(night)	0.121	0.348	-0.92
**Random effects: precision parameter**			
Intercept	0.751	0.866	
F(true)	0.892	0.945	-0.92

	**Fixed effects: mean model**				**Fixed effects: precision model**			
	Estimate	*Std. Error*	*z value*	*p-value*	*Estimate*	*Std. Error*	*z value*	*p-value*
(Intercept)	0.238	0.301	0.79	0.430	0.876	0.587	1.493	0.136
D(night)	0.187	0.148	1.26	0.208	0.250	0.182	1.375	0.169
F(true)	-2.337	0.175	-13.39	**$<2e^{-16}$**	-0.671	0.380	-1.767	0.077
R(perth canyon)	0.807	0.138	5.83	**$5.62e^{-9}$**	1.490	0.544	2.742	**0.006**
R(midwest)	0.380	0.181	2.11	**0.035**	1.024	0.582	1.759	0.079
R(northwest)	0.420	0.234	1.80	0.073	0.985	0.634	1.555	0.120
R(north)	1.167	0.277	4.21	**$2.57e^{-5}$**	1.459	0.644	2.265	**0.023**
*D(night)*F(true)*	2.260	0.203	11.16	**$<2e^{-16}$**	1.502	0.470	3.199	**0.001**

Validation of the full dataset model (*M8b*) Pearson residuals showed a good fit, with no significant patterns against the levels of fixed factors or the linear predictor, and the absolute value of all residuals were less than 4. There was some heteroskedasticity of variance of residuals across individuals; whales with longer time-series tended to have higher variability. While there was minor evidence for temporal and spatial autocorrelation, they were not deemed an issue because only one individual displayed minor evidence of the former, and the latter was only observed in transit areas of the two regions with one whale (Southwest and North). These patterns in the residuals provide interesting behavioural insights that cannot be accounted for with the current dataset, so were retained in M1.

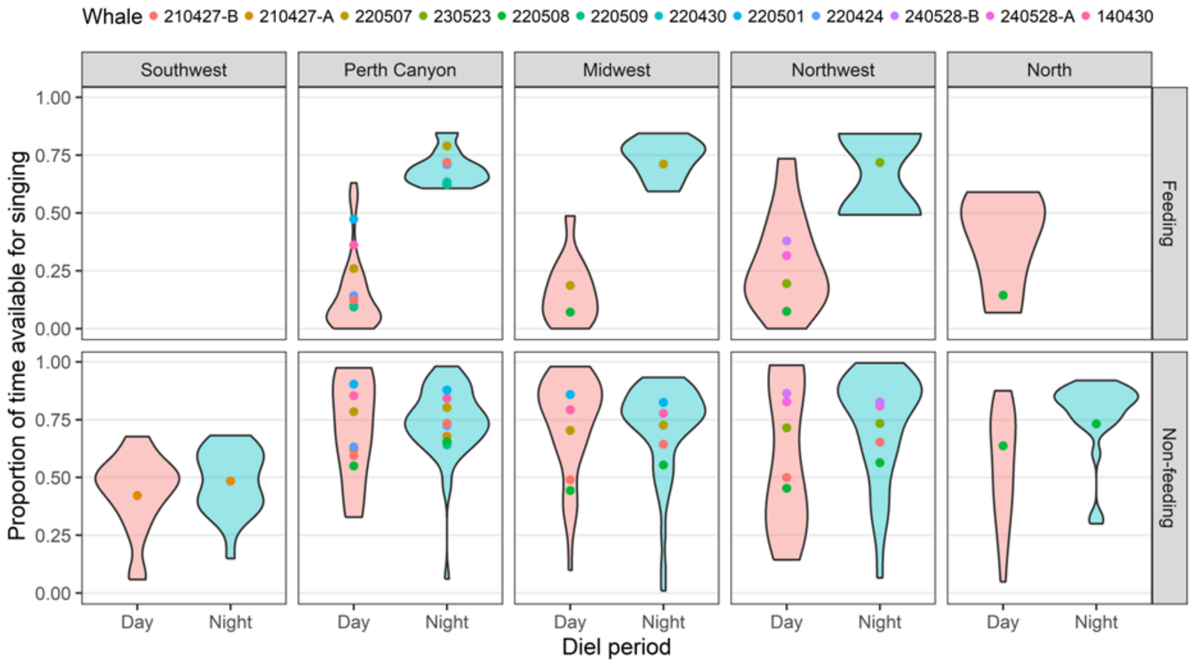

Figure S1. Full final model (M8b from Table S2 or M1 from the paper) Predicted values for each whale individual (points) and violins of raw data distribution for each factor combination.

Table S4. Model selection process for the subset excluding the Southwest region. Symbology is the same as Table S1.

ID	df	AIC	μ fixed	μ random	Φ fixed	Φ random

M9a	20	-396.1407	D*F+R	D|W	D*F+R	F|W
M9b	23	-402.4381	D*F+R*F	D|W	D*F+R	F|W
M9c	23	-394.6033	D*F+R	D|W	D*F+R*F	F|W
M9d	23	-393.8421	D*F+R*D	D|W	D*F+R	F|W
M9e	23	-396.5942	D*F+R	D|W	D*F+R*D	F|W
M9b selected						
M9f	26	-399.1396	D*F+R*F	D|W	D*F+R*F	F|W
M9g	28	-401.5144	D*F*R	D|W	D*F+R	F|W
M9b remains the best with lower AIC and fewer df						

Table S5. Statistical output from the best model of the subset excluding the Southwest region data (M9b), in the top left cell are the model specifications (TAS = time available for singing, D = diel period, F = presence/absence of feeding, R = region, W = whale). The central cell has the parameters for the fixed effects part of the mean model, the random effects are left centre. The right cell shows the parameters for the fixed effects of the precision model, the random effects are left bottom. Significant p-values are in bold.

**Model M9b - Family: beta (logit)**			
*Mean model: TAS ~ D * F + R * F + (D|W)*			
*Precision model: ~ D * F + R + (F|W)*			
**Random effects: mean parameter**			
Name	*Variance*	*Std.Dev.*	*Corr*
Intercept	0.704	0.839	
D(night)	0.135	0.367	-0.94
**Random effects: precision parameter**			
Intercept	0.752	0.867	
F(true)	0.932	0.965	-0.91

**Fixed effects: mean parameter**	*Estimate*	*Std. Error*	*z value*	*p-value*
(Intercept)	1.095	0.284	3.85	**$1.12e^{-4}$**
D(night)	0.165	0.163	1.01	0.312
F(true)	-2.552	0.187	-13.67	**$<2e^{-16}$**
R(midwest)	-0.484	0.128	-3.79	**$1.52e^{-4}$**
R(northwest)	-0.431	0.192	-2.24	**0.025**
R(north)	0.222	0.245	0.91	0.363
D(night) F(true)*	2.386	0.203	11.77	**$<2e^{-16}$**
F(true):R(midwest)	0.199	0.230	0.87	0.386264
F(true):R(northwest)	0.2873	0.3848	0.747	0.455166
F(true):R(north)	1.5839	0.4048	3.913	**$9.12e^{-5}$**

**Fixed effects: precision parameter**	*Estimate*	*Std. Error*	*z value*	*p-value*
(Intercept)	0.876	0.587	1.49	0.136
D(night)	0.250	0.182	1.38	0.169
F(true)	-0.671	0.380	-1.77	0.077
R(perth canyon)	1.490	0.544	2.74	**0.006**
R(midwest)	1.024	0.582	1.76	0.079
R(northwest)	0.985	0.634	1.56	0.120
R(north)	1.459	0.644	2.27	**0.023**
*D(night)*F(true)*	1.502	0.470	3.20	**0.001**

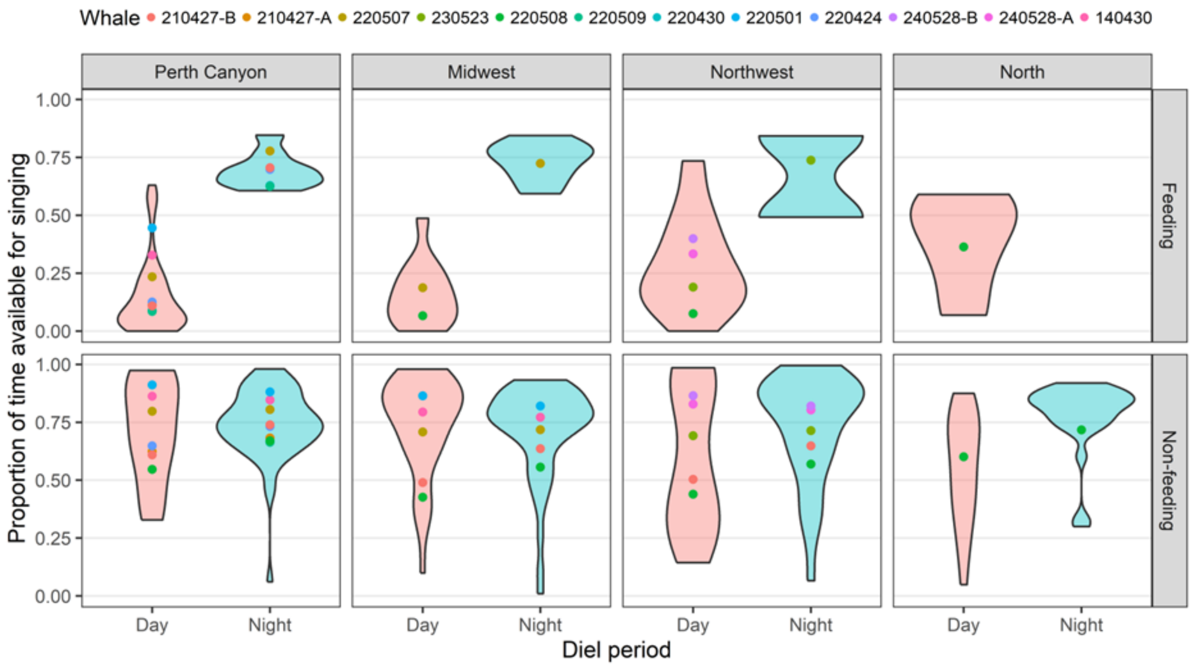

Figure S2. Final subset model (M9b from Table S4 or M2 from the paper) predicted values for each whale individual (points) and violins of raw data distribution for each factor combination.

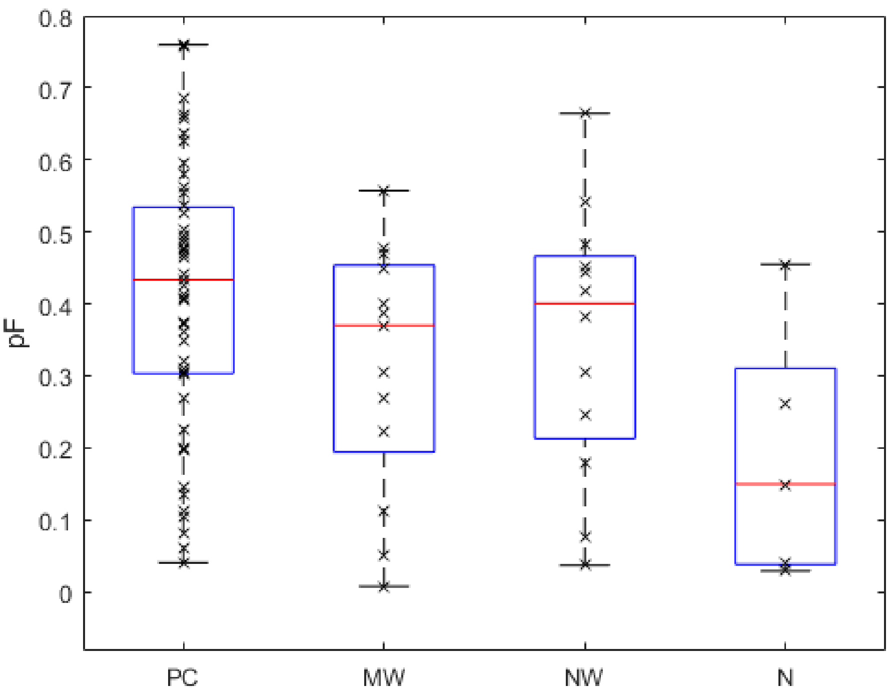

Figure S3. Distributions of proportion of time in feeding dives in each diel period (pF) by region, showing region N with less pF per feeding present diel period, with three out of five feeding periods having a pF value less than 0.2.

Table S6. Model selection process for the subset excluding the Southwest and North regions. Symbology is the same as in Table S1.

ID	df	AIC	μ fixed	μ random	Φ fixed	Φ random
M10a	18	-382.687	D*F+R	D|W	D*F+R	F|W

M10b	20	-379.932	D*F+R*F	D|W	D*F+R	F|W
M10c	20	-380.049	D*F+R	D|W	D*F+R*F	F|W
M10d	20	-380.174	D*F+R*D	D|W	D*F+R	F|W
M10e	20	-379.635	D*F+R	D|W	D*F+R*D	F|W
The full final model remains the best						

Table S7. Statistical output from the best model of the subset excluding the Southwest and North regions data (M10a), in the top left cell are the model specifications (TAS = time available for singing, D = diel period, F = presence/absence of feeding, R = region, W = whale). The central cell has the parameters for the fixed effects part of the mean model, the random effects are left centre. The right cell shows the parameters for the fixed effects of the precision model, the random effects are left bottom. Significant p-values are in bold.

**Model M10a - Family: beta (logit)**			
*Mean model: TAS ~ D * F + R + (D|W)*			
*Precision model: ~ D * F + R + (F|W)*			
**Random effects: mean model**			
Name	*Variance*	*Std.Dev.*	Corr
Intercept	0.737	0.858	
D(night)	0.141	0.376	-0.89
**Random effects: precision model**			
Intercept	0.756	0.869	
F(true)	1.200	1.100	-0.92

	**Fixed effects: mean model**				**Fixed effects: precision model**			
	Estimate	*Std. Error*	*z value*	*p-value*	*Estimate*	*Std. Error*	*z value*	*p-value*
(Intercept)	1.182	0.287	4.13	**3.67e^{-5}**	2.580	0.360	7.23	**4.96e^{-13}**
D(night)	0.053	0.166	0.32	0.749	-0.019	0.219	-0.09	0.930
F(true)	-2.542	0.175	-14.54	**<2e^{-16}**	-0.709	0.429	-1.66	0.098
R(midwest)	-0.442	0.116	-3.83	**1.28e^{-4}**	-0.488	0.222	-2.20	**0.028**
R(northwest)	-0.440	0.195	-2.25	**0.024**	-0.586	0.312	-1.88	0.061
*D(night)*F(true)*	2.476	0.207	11.96	**<2e^{-16}**	1.628	0.487	3.34	**8.37e^{-4}**

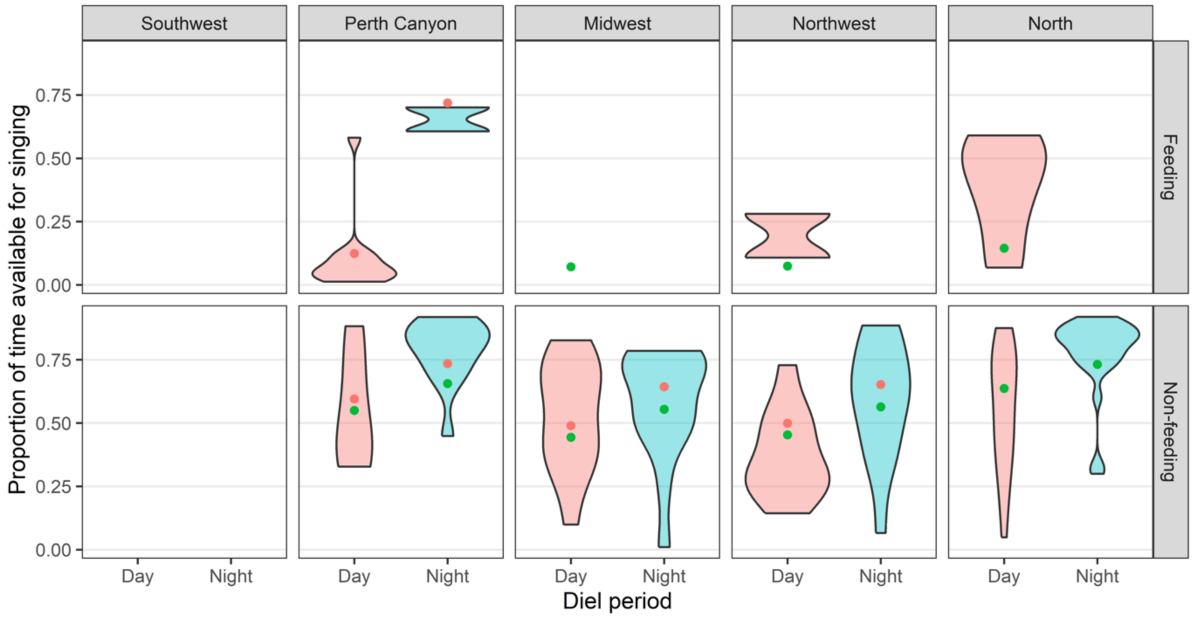

Figure S4. Predicted values for each whale individual (points) and violins of raw data distribution for each factor combination for whales 220508 (green) and 210427-B (red).

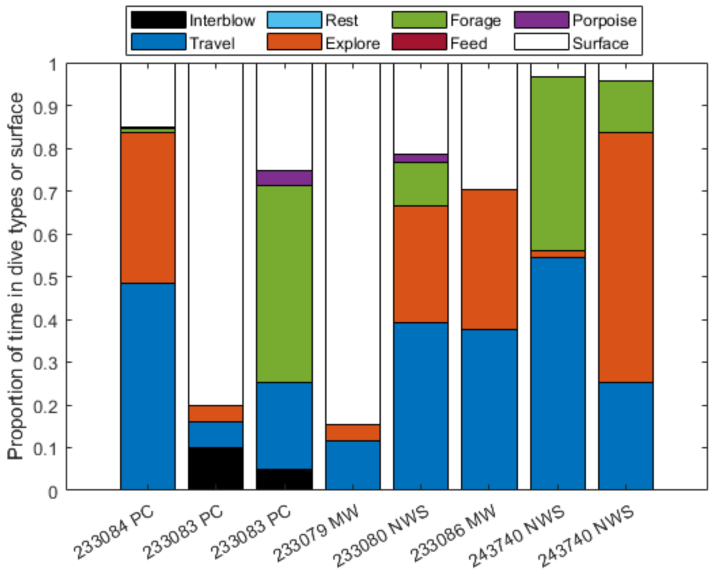

Figure S5.For the 8 non-feeding diel periods where TAS was less than 0.6 and were not included in Figure 4 of the manuscript, behaviour as the proportion of time in dive types and at the surface for the remainder. X-axis labels are the tag serial number followed by the region (PC – Perth Canyon, MW – Midwest, NW – Northwest).

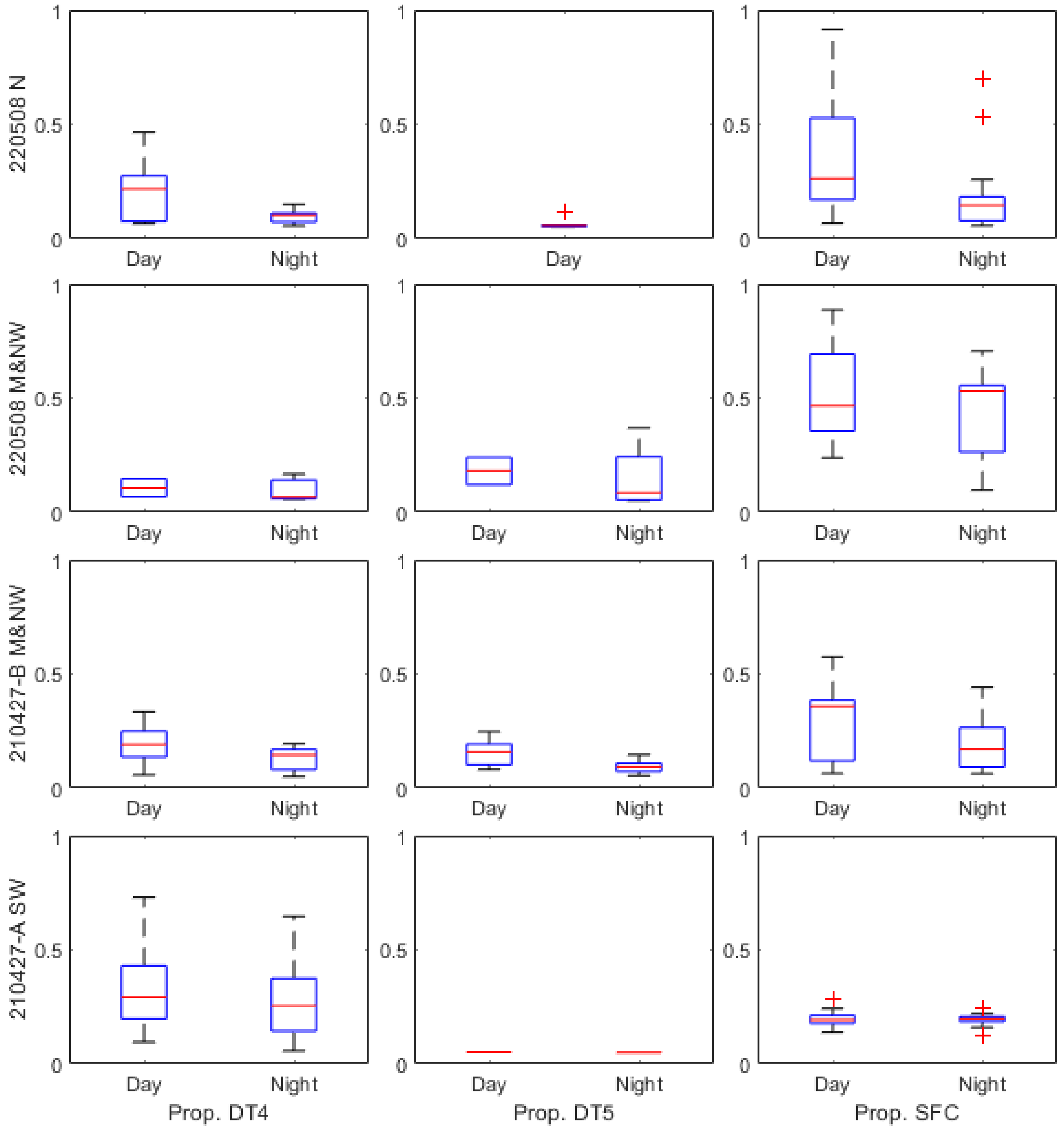

Figure S6. Diel patterns in non-feeding behaviours during the time bounds for each row in from Figure 5 of the Manuscript. Left column is exploratory dives (DT4), middle column is foraging dives (DT5), and right column is surface time (SFC). Y axis are proportions from 0 to 1.

Table S8. Summary statistics of singing behaviour in PAM datasets. # singing days was the count of days where call rate exceeded 5 call h^{-1}, which was calculated daily. The # foraging days was the count of singing days where the average of night divided by day singing rates (D:N ratio) was greater than one. The # bouts was the count of periods when days that the D:N ratio consecutively exceeded one and the # days in bouts is the count of all days in these periods. The maximum duration of a bout (in days) is presented, along with the median and inter-quartile range (IQR: 25th and 75th percentiles).

Set #	Region	Year	MigD	# singing days	# days D:N ratio >1	# bouts	#days in bouts	max bout duration	median bout duration (IQR)
**1**	Perth Canyon	2000	N	33	29	3	29	21	4.0 (4.0–16.8)
**2**	Perth Canyon	2003	N	100	71	10	61	26	3.0 (3.0–7.0)
**3**	Perth Canyon	2004	N	89	79	10	74	14	7.5 (5.0–9.0)
**4**	Perth Canyon	2005	N	120	98	11	86	21	3.0 (2.0–14.5)
**5**	Perth Canyon	2007	N	86	74	10	71	16	5.5 (3.0–12.0)
**6**	Perth Canyon	2008	N	52	34	8	29	6	4.0 (2.5–4.0)
**7**	Perth Canyon	2009	N	87	59	11	47	11	3.0 (2.3–4.0)
**8**	Perth Canyon	2010	N	127	85	18	70	8	3.0 (2.0–6.0)
**9**	Perth Canyon	2011	N	73	54	11	48	10	3.0 (2.0–6.5)
**10**	Perth Canyon	2014	N	164	117	19	105	23	3.0 (2.0–6.8)
**11**	Northwest	2011	S	31	11	3	7	3	2.0 (2.0–2.8)
**12**	Northwest	2012	N	125	75	17	61	17	2.0 (2.0–3.3)
**13**	Northwest	2012	N	71	44	8	19	3	2.0 (2.0–3.0)
**14**	Northwest	2012	S	12	7	1	4	4	4.0 (4.0–4.0)
**15**	Northwest	2013	N	66	36	6	21	9	2.5 (2.0–3.0)
**16**	Northwest	2014	S	47	22	6	13	3	2.0 (2.0–2.0)
**17**	Northwest	2015	N	50	33	9	29	6	3.0 (2.0–4.3)
**18**	Northwest	2018	N	58	23	6	14	4	2.0 (2.0–2.0)
**19**	Northwest	2018	S	54	19	3	6	2	2.0 (2.0–2.0)
**20**	Northwest	2019	N	99	42	9	27	6	3.0 (2.0–3.0)
**21**	Northwest	2019	S	58	21	4	8	2	2.0 (2.0–2.0)
**22**	North	2017	S	0	0	n/a	n/a	n/a	n/a
**23**	North	2017	S	1	1	0	0	n/a	n/a
**24**	North	2018	N	2	1	0	0	n/a	n/a
**25**	North	2017	S	1	0	n/a	n/a	n/a	n/a

**26**	North	2018	N	24	12	1	6	6	6.0 (6.0–6.0)
**27**	North	2017	S	2	1	0	0	n/a	n/a
**28**	North	2018	N	5	3	1	2	2	2.0 (2.0–2.0)
**29**	North	2017	S	4	1	0	0	n/a	n/a
**30**	North	2018	N	9	6	0	0	n/a	n/a
**31**	North	2017	S	11	2	0	0	n/a	n/a
**32**	North	2018	N	15	8	2	4	2	2.0 (2.0–2.0)
**33**	North	2017	S	21	5	1	2	2	2.0 (2.0–2.0)
**34**	North	2018	N	27	14	3	8	3	3.0 (2.3–3.0)
**35**	North	2017	S	34	12	3	8	4	2.0 (2.0–3.5)
**36**	North	2018	N	34	14	3	7	3	2.0 (2.0–2.8)

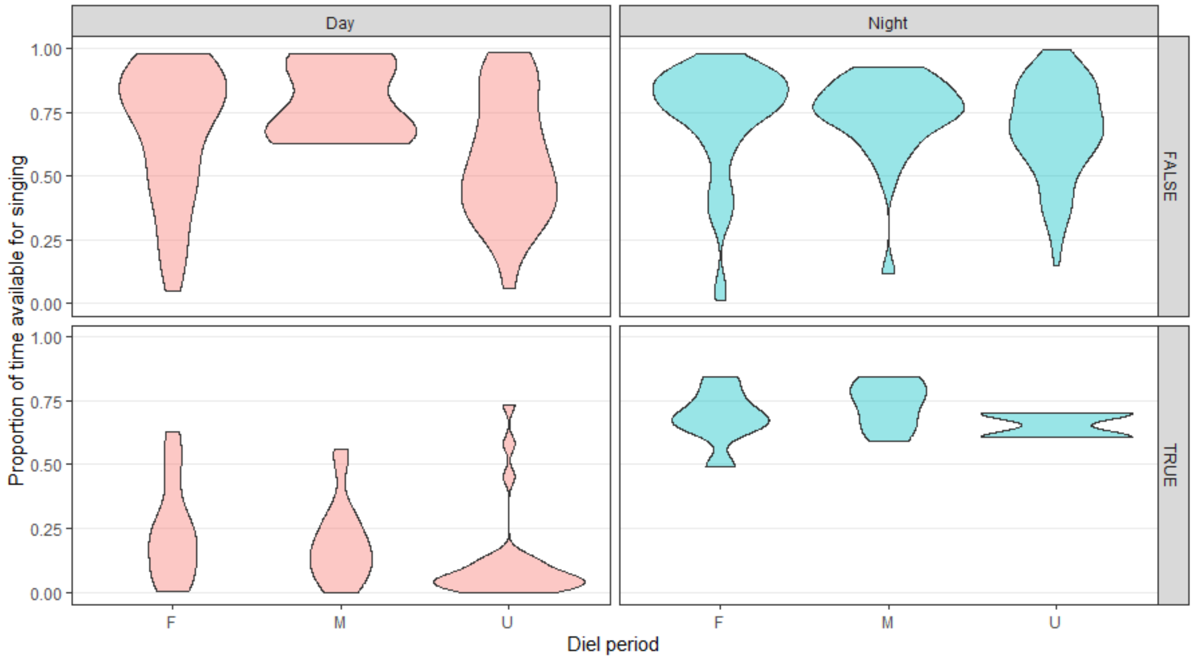

Figure S7. Violin plot of the raw proportion of time available for singing by day or night period, presence (TRUE) or absence (FALSE) of feeding in the period, and the sex of the whale: female (F), male (M), or unidentified (U).
