# Supplementary material for: Pygmy blue whale feeding drives greater nocturnal than diurnal song production
Source: R Soc Open Sci. 2026 Oct 28;13(10):260954. doi: 10.1098/rsos.260954 (PMC13714375; doi:10.1098/rsos.260954)
Supplement: Supplementary file 1 [file rsos.260954.zip › rsos.260954/rsos260954_si_003.pdf]

Pygmy blue whale feeding drives greater nocturnal than diurnal song production

AUTHOR
Andrew M. Davenport, Michele Thums, K. Curt S. Jenner, Micheline-Nicole M. Jenner, Luciana C. Ferreira, Christine Erbe, Ryan Leadbetter, Robert D. McCauley

PUBLISHED
August 18, 2025

Royal Society Open Science (DOI: 10.1098/rsos.260954)

Supplementary materials: statistical analysis code with markup

Load packages and data exploration

```
library(dplyr)
library(ggplot2)
library(readxl)
library(cowplot)
library(lme4)
library(betareg)
library(lmtest)
library(glmmTMB)
library(lattice)
```

Load data and look at the headers.

```
data <- read.csv("F:/M/data_ch2/pt23_07Apr2025_133101.csv"
              )%>%
select(
    pt23,    # proportion of time available for singing
    diel,  # day/night
    reg,   # region
    feed,  # feeding
    id,     # individual
    lat,
    lon,
    day
  ) %>%
mutate(
    diel = factor(diel,ordered = FALSE),
    reg = factor(reg, ordered = FALSE),
    feed = as.logical(feed),
    id = factor(as.character(id), ordered = FALSE)
)

head(data)
```

```
         pt23 diel reg  feed     id      lat      lon day
1 0.006754059    1   2  TRUE 214432 -32.1000 115.1250 0.5
2 0.631912073    2   2 FALSE 214432 -32.1000 115.0750 1.0
3 0.072644462    1   2  TRUE 214432 -32.1500 114.9500 1.5
```

```
4 0.683820092    2   2 FALSE 214432 -32.1375 115.0500 2.0
5 0.041595384    1   2  TRUE 214432 -32.1250 115.0500 2.5
6 0.730997864    2   2 FALSE 214432 -32.1000 115.0417 3.0
```

Assign the levels of the factors, summary stats of columns.

```
levels(data$reg) <- c("Southwest","Perth Canyon","Midwest","Northwest","North")
levels(data$diel) <- c("Day","Night")
data %>% summary()
```

```
      pt23            diel                 reg          feed              id    
 Min.   :0.0000   Day  :199   Southwest   : 42   Mode :logical   233082 :78  
 1st Qu.:0.3666   Night:206   Perth Canyon:129   FALSE:306       214430 :62  
 Median :0.6574               Midwest     :117   TRUE :99        214432 :62  
 Mean   :0.5811               Northwest   : 69                   233079 :53  
 3rd Qu.:0.8210               North       : 48                   233086 :53  
 Max.   :0.9948                                                  243740 :25  
                                                                 (Other):72  
      lat                 lon             day       
 Min.   :-35.792   Min.   :111.9   Min.   : 0.50  
 1st Qu.:-31.785   1st Qu.:113.6   1st Qu.: 4.50  
 Median :-28.802   Median :114.7   Median :11.50  
 Mean   :-26.736   Mean   :115.3   Mean   :13.46  
 3rd Qu.:-22.195   3rd Qu.:115.1   3rd Qu.:21.00  
 Max.   : -6.886   Max.   :126.2   Max.   :41.00  
```

Get sample size across the fixed factors.

```
data %>%     group_by(reg, feed,diel) %>%     summarise(n(),.groups="rowwise")
```

```
# A tibble: 17 × 4
# Rowwise:  reg, feed, diel
   reg          feed  diel  `n()`
   <fct>        <lgl> <fct> <int>
 1 Southwest    FALSE Day      21
 2 Southwest    FALSE Night    21
 3 Perth Canyon FALSE Day      14
 4 Perth Canyon FALSE Night    55
 5 Perth Canyon TRUE  Day      51
 6 Perth Canyon TRUE  Night     9
 7 Midwest      FALSE Day      44
 8 Midwest      FALSE Night    53
 9 Midwest      TRUE  Day      13
10 Midwest      TRUE  Night     7
11 Northwest    FALSE Day      21
12 Northwest    FALSE Night    34
13 Northwest    TRUE  Day      12
14 Northwest    TRUE  Night     2
15 North        FALSE Day      18
16 North        FALSE Night    25
17 North        TRUE  Day       5
```

Get sample sizes for Figure 1 (map).

```
z <- which(data$feed == TRUE)
a <-  data[z,] %>%     group_by(reg) %>%     summarise(n())
b <- data %>%     group_by(reg) %>%     summarise(n())
c <- data[z,] %>%     group_by(reg) %>%     summarise(n_distinct(id))
d <- data %>%     group_by(reg) %>%     summarise(n_distinct(id))
tab <- b %>% left_join(d,by="reg") %>% left_join(a,by="reg") %>% left_join(c,by="reg")
tab %>% setNames(c("reg","nTotal",'nWTotal',"nFeed","nWFeed"))
```

```
# A tibble: 5 × 5
  reg          nTotal nWTotal nFeed nWFeed
  <fct>         <int>   <int> <int>  <int>
1 Southwest        42       1    NA     NA
2 Perth Canyon    129       9    60      8
3 Midwest         117       5    20      2
4 Northwest        69       5    14      4
5 North            48       1     5      1
```

A simple visualization to comparing the time available for singing (TAS = proportion of time in dive types 2 & 3) by day (1) and night (2) and presence/ absence of feeding.

```
data %>%     ggplot() +
  geom_violin(aes(x = feed, y = pt23, fill = feed), alpha = 0.4) +
  geom_jitter(aes(x = feed, y = pt23, col = diel), width = 0.05)
```

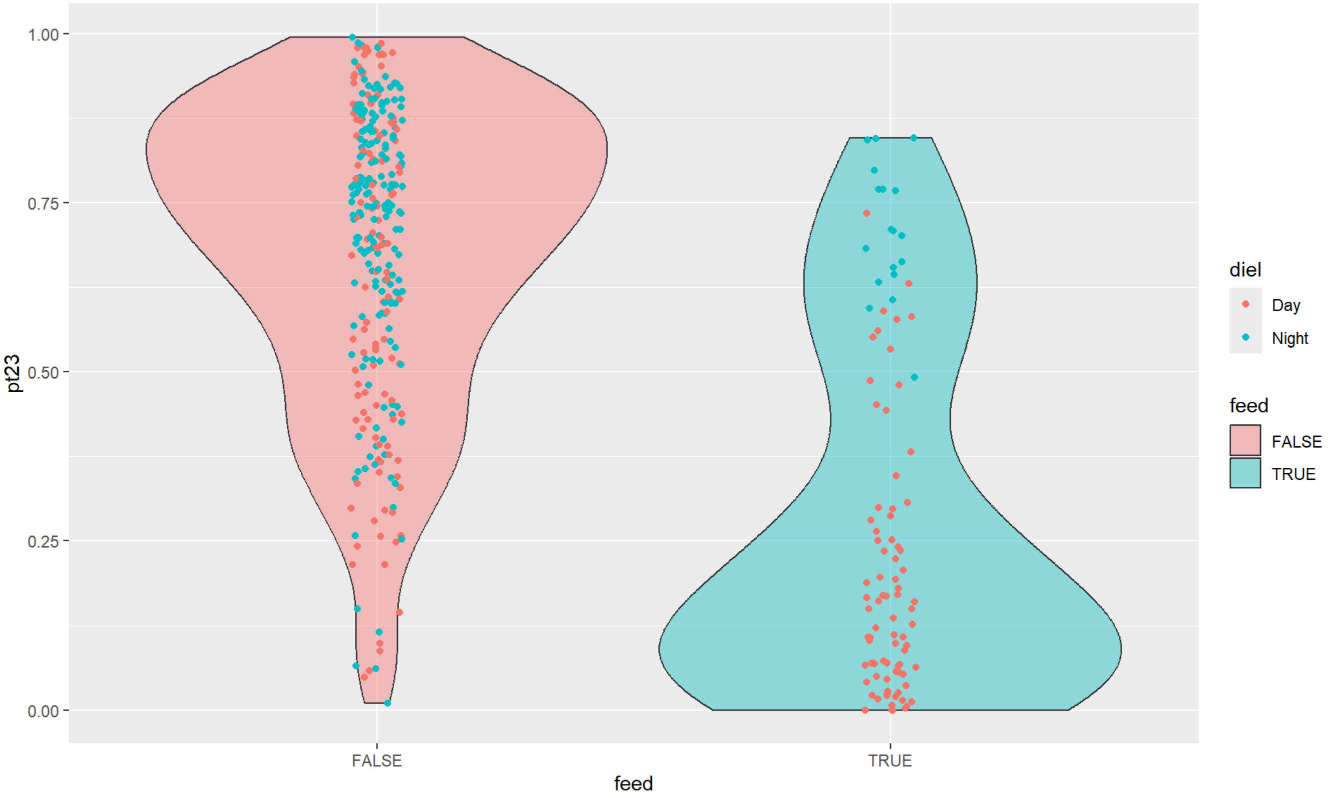

It shows a difference but there are deeper patterns in the data than this simple visualization.

Lets look at the data by each factor combination.

```
data %>%     ggplot() +
  geom_jitter(aes(x = diel, y = pt23, col = id), width = 0.1) +
  facet_grid(rows = vars(feed),cols = vars(reg))
```

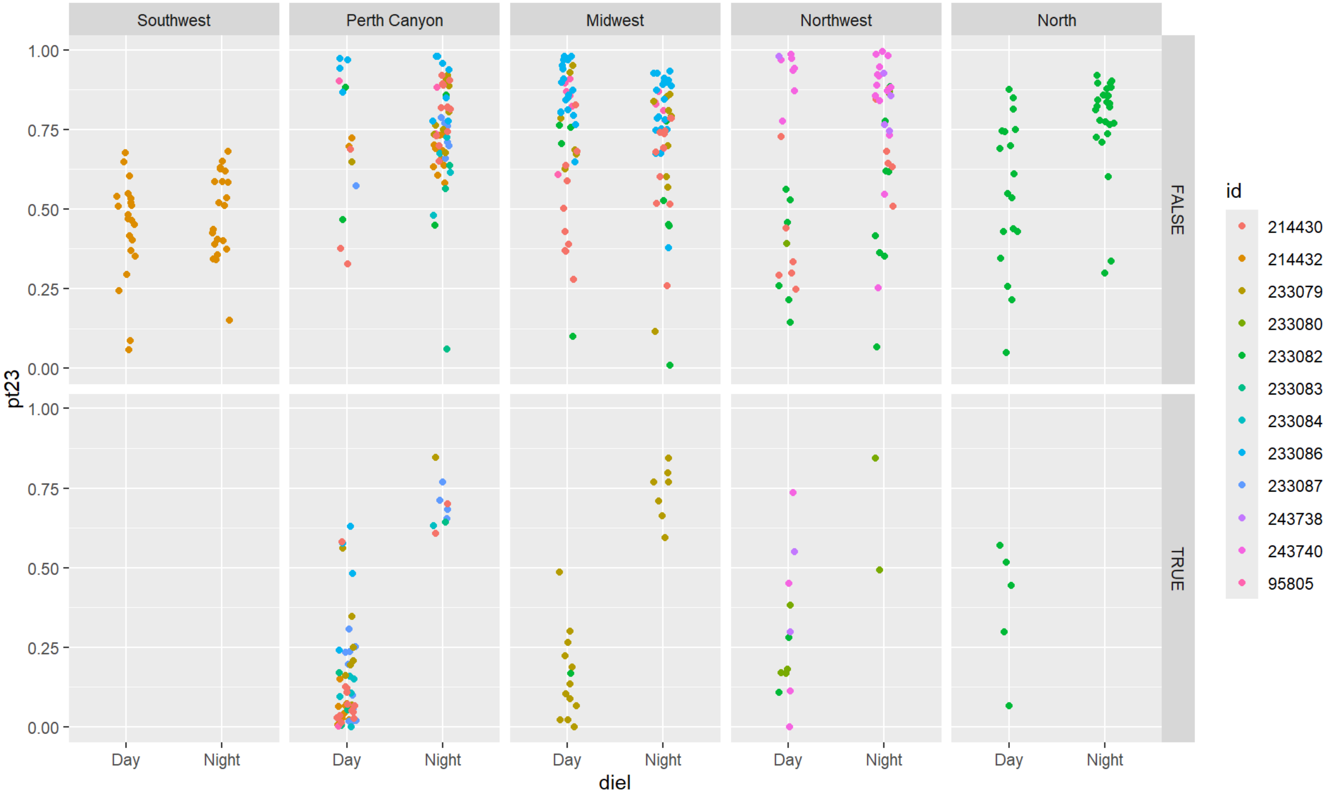

There was some regional variation and non-feeding days with low TAS. Generally TAS is only really low for feeding days, but not feeding nights.

Modelling: finding the best conditional expression

To start, add a very small number to 0 values so that it can numerically compute on a standard beta distribution. This is justified because the probability of time available for singing is unlikely to be zero.

```
cat(length(which(data$pt23 == 0)),"zeros adjusted")
```

```
3 zeros adjusted
```

```
data$pt23_jitter <- ifelse(data$pt23 == 0, data$pt23 + 0.001, data$pt23)
```

Below, all combinations of the fixed factors and their interactions were modeled on the conditional expression.

```
M0 <- betareg(pt23_jitter ~ diel * reg + feed,
                 data = data)
#M0a <- betareg(pt23_jitter ~ diel * reg * feed,
#                data = data) #did not converge - so had to remove
M0b <- betareg(pt23_jitter ~ diel + reg + feed,
                 data = data)
#M0c <- betareg(pt23_jitter ~ diel + reg * feed,
```

```
#                  data = data) #did not converge - so had to remove
M0d <- betareg(pt23_jitter ~ diel * feed + reg,
                 data = data)
M0e <- betareg(pt23_jitter ~ diel * reg,
                 data = data)
M0f <- betareg(pt23_jitter ~ diel * feed,
                 data = data)
#M0g <- betareg(pt23_jitter ~ feed * reg,
#                  data = data) #did not converge - so had to remove
M0h <- betareg(pt23_jitter ~ diel + reg,
                 data = data)
M0i <- betareg(pt23_jitter ~ diel + feed,
                 data = data)
M0j <- betareg(pt23_jitter ~ feed + reg,
                 data = data)
M0k <- betareg(pt23_jitter ~ diel,
                 data = data)
M0l <- betareg(pt23_jitter ~ reg,
                 data = data)
M0m <- betareg(pt23_jitter ~ feed,
                 data = data)
M0n <- betareg(pt23_jitter ~ diel * feed + reg*diel,
                 data = data)
A <- AIC(M0,M0b,M0d,M0e,M0f,M0h,M0i,M0j,M0k,M0l,M0m,M0n)
A <- A[order(A$AIC),]
print.data.frame(A)
```

```
    df        AIC
M0d  9 -292.81507
M0n 13 -291.17011
M0f  5 -255.97932
M0  12 -238.68934
M0b  8 -236.96415
M0j  7 -217.57367
M0i  4 -207.82353
M0m  3 -188.04453
M0e 11 -133.56753
M0h  7 -102.04974
M0k  3  -77.41980
M0l  6  -30.00473
```

The three way interaction and region:feed interactions did not converge due to a lack of samples for some combinations of the latter two factors.

The region:diel interaction was not as important as the diel:feed interaction that significantly reduced the AIC.

So we move on with the M0d model which had the lowest AIC.

Finding the best precision expression

We now demonstrate the full forward stepwise approach to find the best precision expression (lowest AIC). Changing a single term in the precision part of the expression (after the ' | ') at a time, and proceeding with the expression that had the lowest AIC.

```
M1a <- betareg(pt23_jitter ~ diel * feed + reg | feed,
                 data = data)
M1b <- betareg(pt23_jitter ~ diel * feed + reg | diel,
                 data = data)
M1c <- betareg(pt23_jitter ~ diel * feed + reg | reg,
                 data = data)

AIC(M0d,M1a,M1b,M1c)
```

```
    df       AIC
M0d  9 -292.8151
M1a 10 -291.5988
M1b 10 -301.6509
M1c 13 -308.2772
```

M1c had lowest AIC

```
M2a <- betareg(pt23_jitter ~ diel * feed + reg | reg+feed,
                 data = data)
M2b <- betareg(pt23_jitter ~ diel * feed + reg | reg+diel,
                 data = data)
AIC(M1c,M2a,M2b)
```

```
    df       AIC
M1c 13 -308.2772
M2a 14 -306.8623
M2b 14 -318.6276
```

M2b had lowest AIC

```
M3a <- betareg(pt23_jitter ~ diel * feed + reg | reg+feed+diel,
                 data = data)
M3b <- betareg(pt23_jitter ~ diel * feed + reg | reg*diel,
                 data = data)
AIC(M2b,M3a,M3b)
```

```
    df       AIC
M2b 14 -318.6276
M3a 15 -323.1883
M3b 18 -313.1840
```

M3a had lowest AIC

```
#did not converge M4a <- betareg(pt23_jitter ~ diel * feed + reg | reg*feed+diel,
#                  data = data) #did not converge - so had to remove
M4b <- betareg(pt23_jitter ~ diel * feed + reg | reg*diel+feed,
                 data = data)
M4c <- betareg(pt23_jitter ~ diel * feed + reg | reg+diel*feed,
```

```
                 data = data)

AIC(M3a,M4b,M4c)
```

```
    df       AIC
M3a 15 -323.1883
M4b 19 -319.9106
M4c 16 -330.6736
```

M4c had lowest AIC

```
M5a <- betareg(pt23_jitter ~ diel * feed + reg | reg*diel+diel*feed,
                 data = data) #did not converge - so had to remove

A <- AIC(M0d,M1a,M1b,M1c,M2a,M2b,M3a,M3b,M4b,M4c,M5a)
A <- A[order(A$AIC),]
print.data.frame(A)
```

```
    df       AIC
M4c 16 -330.6736
M5a 20 -326.1898
M3a 15 -323.1883
M4b 19 -319.9106
M2b 14 -318.6276
M3b 18 -313.1840
M1c 13 -308.2772
M2a 14 -306.8623
M1b 10 -301.6509
M0d  9 -292.8151
M1a 10 -291.5988
```

All models including a precision expression rather than fixed phi were significantly better. M4c was the best, which was the precision expression identical to the conditional expression.

Lets have a look at the output of our best model without random effects

```
summary(M4c)
```

```

Call:
betareg(formula = pt23_jitter ~ diel * feed + reg | reg + diel * feed,
    data = data)

Quantile residuals:
    Min      1Q  Median      3Q     Max
-5.1233 -0.5558  0.1149  0.6622  2.2559

Coefficients (mean model with logit link):
                   Estimate Std. Error z value Pr(>|z|)
(Intercept)         -0.3195     0.1135  -2.814  0.00489 **
dielNight            0.2584     0.1057   2.446  0.01446 *
feedTRUE            -2.1742     0.1627 -13.361  < 2e-16 ***
regPerth Canyon      0.9714     0.1293   7.512 5.84e-14 ***
```

```
regMidwest           1.0346     0.1271   8.141 3.91e-16 ***
regNorthwest         1.0254     0.1566   6.548 5.82e-11 ***
regNorth             1.0278     0.1574   6.529 6.61e-11 ***
dielNight:feedTRUE   2.1207     0.1943  10.916  < 2e-16 ***

Phi coefficients (precision model with log link):
                   Estimate Std. Error z value Pr(>|z|)
(Intercept)          2.0231     0.2203   9.181  < 2e-16 ***
regPerth Canyon     -0.7790     0.2507  -3.107 0.001888 **
regMidwest          -0.9244     0.2412  -3.833 0.000127 ***
regNorthwest        -1.3318     0.2580  -5.162 2.44e-07 ***
regNorth            -0.7100     0.2804  -2.532 0.011343 *
dielNight            0.4902     0.1532   3.200 0.001375 **
feedTRUE             0.2494     0.2114   1.180 0.238135
dielNight:feedTRUE   1.3748     0.3976   3.458 0.000544 ***
---
Signif. codes:  0 '***' 0.001 '**' 0.01 '*' 0.05 '.' 0.1 ' ' 1

Type of estimator: ML (maximum likelihood)
Log-likelihood: 181.3 on 16 Df
Pseudo R-squared: 0.492
Number of iterations: 27 (BFGS) + 1 (Fisher scoring)
```

All terms have at least one level highly significant.

Lets look at a validation plot of Pearson residuals.

```
data$E <- resid(M4c,type="pearson")
z <- 1:nrow(data)
p_index <- data %>%
  ggplot() +
  geom_point(aes(x = z, y = E, color = id)) +
  ylim(-4, 3) +
  xlim(0, 450)
p_index
```

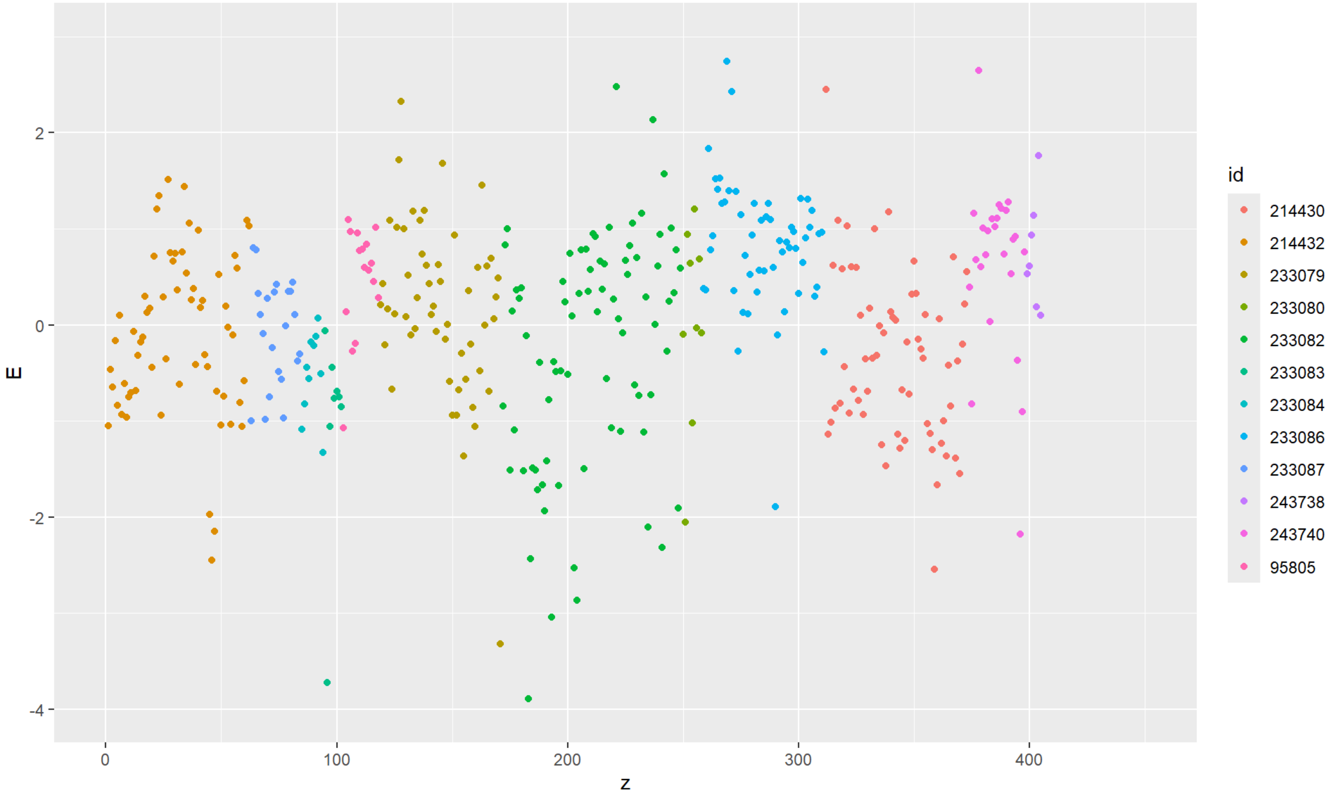

No obvious temporal autocorrelation.

Residuals between whales seem to have similar variability, but there are still a few whales that are more clustered.

Account for whale dependence as a random effect

first fit the base model (M4c as M5) in glmmTMB

```
M5 <- glmmTMB(pt23_jitter ~ diel * feed + reg,
                dispformula =~ diel * feed + reg,
                family="beta_family",
                data = data)
summary(M5)
```

```
 Family: beta  ( logit )
Formula:          pt23_jitter ~ diel * feed + reg
Dispersion:                   ~diel * feed + reg
Data: data

     AIC      BIC   logLik deviance df.resid
  -330.7   -266.6    181.3   -362.7      389


Conditional model:
                   Estimate Std. Error z value Pr(>|z|)
(Intercept)         -0.3195     0.1150  -2.778  0.00548 **
dielNight            0.2584     0.1093   2.365  0.01802 *
feedTRUE            -2.1742     0.1713 -12.694  < 2e-16 ***
regPerth Canyon      0.9714     0.1316   7.379 1.60e-13 ***
```

```
regMidwest           1.0346     0.1284    8.059 7.71e-16 ***
regNorthwest         1.0254     0.1595    6.431 1.27e-10 ***
regNorth             1.0278     0.1625    6.325 2.53e-10 ***
dielNight:feedTRUE   2.1207     0.2002   10.592  < 2e-16 ***
---
Signif. codes:  0 '***' 0.001 '**' 0.01 '*' 0.05 '.' 0.1 ' ' 1

Dispersion model:
                   Estimate Std. Error z value Pr(>|z|)
(Intercept)          2.0231     0.2204    9.180  < 2e-16 ***
dielNight            0.4902     0.1586    3.091 0.001998 **
feedTRUE             0.2494     0.2170    1.150 0.250291
regPerth Canyon     -0.7790     0.2562   -3.040 0.002363 **
regMidwest          -0.9244     0.2428   -3.807 0.000141 ***
regNorthwest        -1.3318     0.2577   -5.168 2.36e-07 ***
regNorth            -0.7100     0.2829   -2.509 0.012093 *
dielNight:feedTRUE   1.3748     0.4035    3.407 0.000658 ***
---
Signif. codes:  0 '***' 0.001 '**' 0.01 '*' 0.05 '.' 0.1 ' ' 1
```

Compared to the model in betareg, the coefficients are basically identical.

Next, forward stepwise selection of the random effects expression.

```
M5b <- glmmTMB(pt23_jitter ~ diel * feed + reg + (1|id),
                dispformula =~ diel * feed + reg,
                family="beta_family",
                data = data)
M5c <- glmmTMB(pt23_jitter ~ diel * feed + reg ,
                dispformula =~ diel * feed + reg + (1|id),
                family="beta_family",
                data = data)
AIC(M5,M5b,M5c)
```

```
    df       AIC
M5  16 -330.6736
M5b 17 -409.7161
M5c 17 -342.6585
```

A random effect on the intercept of the conditional model had lowest AIC (M5b)

```
M6a <- glmmTMB(pt23_jitter ~ diel * feed + reg + (1|id),
                dispformula =~ diel * feed + reg + (1|id),
                family="beta_family",
                data = data)
#M6b <- glmmTMB(pt23_jitter ~ diel * feed + reg+ (reg|id),
#                dispformula =~ diel * feed + reg,
#                family="beta_family",
#                data = data)
#M6b had false convergence
M6c <- glmmTMB(pt23_jitter ~ diel * feed + reg+ (diel|id),
                dispformula =~ diel * feed + reg,
                family="beta_family",
```

```
                data = data)
M6d <- glmmTMB(pt23_jitter ~ diel * feed + reg + (feed|id),
                dispformula =~ diel * feed + reg ,
                family="beta_family",
                data = data)

AIC(M5b,M6a,M6c,M6d)
```

```
    df       AIC
M5b 17 -409.7161
M6a 18 -415.8312
M6c 19 -411.5561
M6d 19 -414.3320
```

A random effect on the intercept for both models was best.

```
#cat(paste(" ","M7a"," ",sep="\n"))
M7a <- glmmTMB(pt23_jitter ~ diel * feed + reg+ (diel|id),
                dispformula =~ diel * feed + reg + (1|id),
                family="beta_family",
                data = data)
#cat(paste(" ","M7b"," ",sep="\n"))
M7b <- glmmTMB(pt23_jitter ~ diel * feed + reg + (feed|id),
                dispformula =~ diel * feed + reg + (1|id),
                family="beta_family",
                data = data)
#cat(paste(" ","M7c"," ",sep="\n"))
#M7c <- glmmTMB(pt23_jitter ~ diel * feed + reg + (reg|id),
#                dispformula =~ diel * feed + reg + (1|id),
#                family="beta_family",
#                data = data)
# M7c did not converge
#cat(paste(" ","M7d"," ",sep="\n"))
#M7d <- glmmTMB(pt23_jitter ~ diel * feed + reg + (1|id),
#                dispformula =~ diel * feed + reg + (diel|id),
#                family="beta_family",
#                data = data)
#M7d had singular convergence
#cat(paste(" ","M7e"," ",sep="\n"))
#M7e <- glmmTMB(pt23_jitter ~ diel * feed + reg + (1|id),
#                dispformula =~ diel * feed + reg + (feed|id),
#                family="beta_family",
#                data = data)
#M7e had singular convergence
#cat(paste(" ","M7f"," ",sep="\n"))
#M7f <- glmmTMB(pt23_jitter ~ diel * feed + reg + (1|id),
#                dispformula =~ diel * feed + reg + (reg|id),
#                family="beta_family",
#                data = data)
# M7f had false convergence

AIC(M6a,M7a,M7b)#,M7c,M7d,M7e,M7f)
```

```
    df       AIC
M6a 18 -415.8312
M7a 20 -423.3715
M7b 20 -420.0301
```

id as a random effect on diel and the intercept for the conditional model was an improvement.

```
#cat(paste(" ","M8a"," ",sep="\n"))
M8a <- glmmTMB(pt23_jitter ~ diel * feed + reg+ (diel|id),
                dispformula =~ diel * feed + reg + (diel|id),
                family="beta_family",
                data = data)
#cat(paste(" ","M8b"," ",sep="\n"))
M8b <- glmmTMB(pt23_jitter ~ diel * feed + reg + (diel|id),
                dispformula =~ diel * feed + reg + (feed|id),
                family="beta_family",
                data = data)
#cat(paste(" ","M8c"," ",sep="\n"))
#M8c <- glmmTMB(pt23_jitter ~ diel * feed + reg + (diel|id),
#                dispformula =~ diel * feed + reg + (reg|id),
#                family="beta_family",
#                data = data)
#M8c singular convergence
AIC(M7a,M8a,M8b)#,M8c)
```

```
    df       AIC
M7a 20 -423.3715
M8a 22 -423.7207
M8b 22 -428.5196
```

```
D <- AIC(M5,M5b,M5c,M6a,M6c,M6d,M7a,M7b,M8a,M8b)
D <- D[order(D$AIC),]
print.data.frame(D)
```

```
    df       AIC
M8b 22 -428.5196
M8a 22 -423.7207
M7a 20 -423.3715
M7b 20 -420.0301
M6a 18 -415.8312
M6d 19 -414.3320
M6c 19 -411.5561
M5b 17 -409.7161
M5c 17 -342.6585
M5  16 -330.6736
```

After testing all numerically possible combinations of random effects expressions, the best AIC was with id as a random effect on diel in the conditional model, and id as a random effect on feed in the dispersion model.

Validation of the best model

```r
M_best <- M8b
summary(M_best)
```

```
 Family: beta  ( logit )
Formula:          pt23_jitter ~ diel * feed + reg + (diel | id)
Dispersion:                   ~diel * feed + reg + (feed | id)
Data: data

     AIC      BIC   logLik deviance df.resid
  -428.5   -340.4    236.3   -472.5      383

Random effects:

Conditional model:
 Groups Name        Variance Std.Dev. Corr
 id     (Intercept) 0.6931   0.8325
        dielNight   0.1213   0.3483   -0.92
Number of obs: 405, groups:  id, 12

Dispersion model:
 Groups Name        Variance Std.Dev. Corr
 id     (Intercept) 0.7507   0.8664
        feedTRUE    0.8921   0.9445   -0.92
Number of obs: 405, groups:  id, 12

Conditional model:
                   Estimate Std. Error z value Pr(>|z|)
(Intercept)          0.2376     0.3007   0.790   0.4295
dielNight            0.1867     0.1483   1.260   0.2078
feedTRUE            -2.3373     0.1745 -13.391  < 2e-16 ***
regPerth Canyon      0.8065     0.1384   5.828 5.62e-09 ***
regMidwest           0.3801     0.1805   2.105   0.0353 *
regNorthwest         0.4195     0.2336   1.796   0.0725 .
regNorth             1.1667     0.2772   4.208 2.57e-05 ***
dielNight:feedTRUE   2.2604     0.2025  11.160  < 2e-16 ***
---
Signif. codes:  0 '***' 0.001 '**' 0.01 '*' 0.05 '.' 0.1 ' ' 1

Dispersion model:
                   Estimate Std. Error z value Pr(>|z|)
(Intercept)          0.8758     0.5868   1.493  0.13557
dielNight            0.2501     0.1819   1.375  0.16912
feedTRUE            -0.6709     0.3798  -1.767  0.07728 .
regPerth Canyon      1.4904     0.5435   2.742  0.00610 **
regMidwest           1.0238     0.5822   1.759  0.07866 .
regNorthwest         0.9852     0.6336   1.555  0.11998
regNorth             1.4588     0.6439   2.265  0.02349 *
dielNight:feedTRUE   1.5018     0.4695   3.199  0.00138 **
---
Signif. codes:  0 '***' 0.001 '**' 0.01 '*' 0.05 '.' 0.1 ' ' 1
```

All terms were important.

```
mu <- predict(M_best,type="response")
res <- data$pt23_jitter-mu
phi <- predict(M_best,type="disp")
v <- mu*(1-mu)/(1+phi)
data$E <- res/sqrt(v)
p_index <- data %>%
  ggplot() +
  geom_point(aes(x = z, y = E, color = id)) +
  ylim(-4.1, 4) +
  xlim(0, 450)
p_index
```

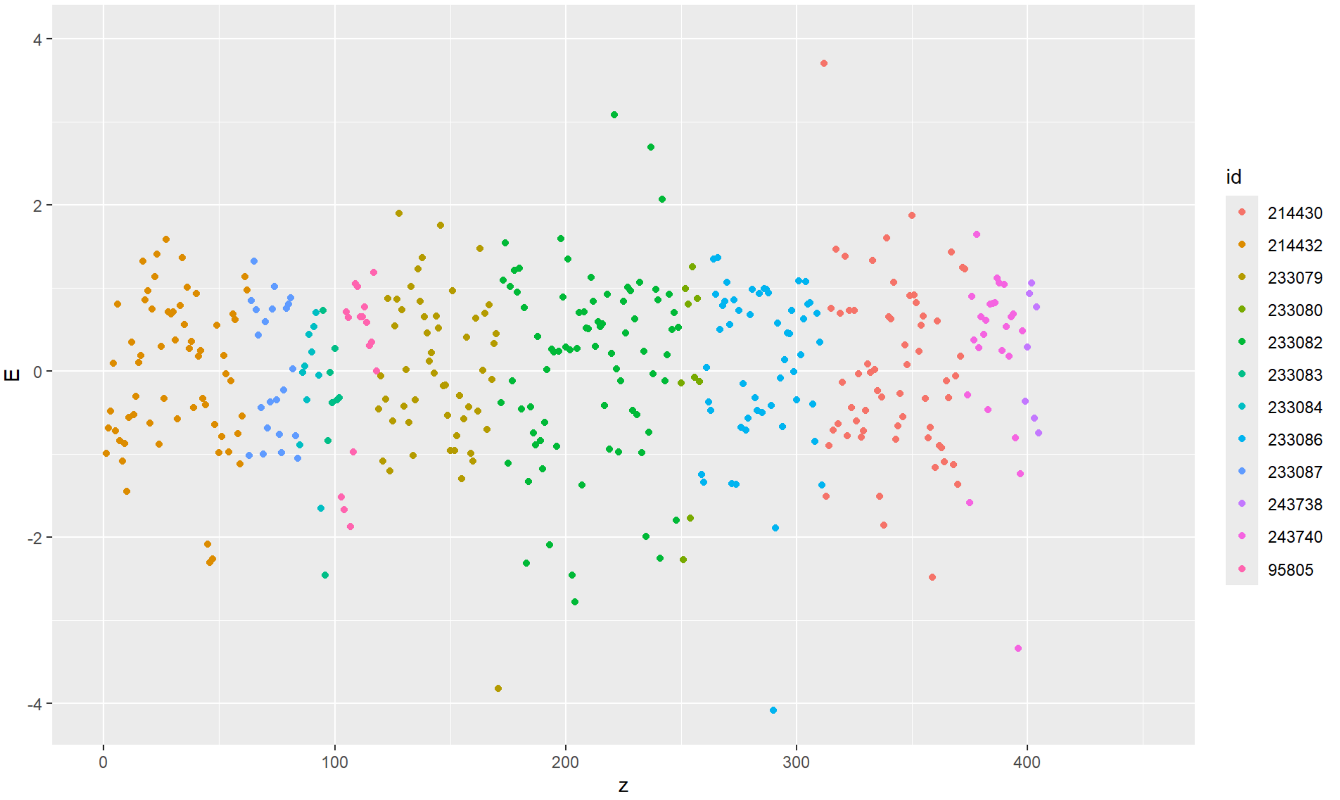

There doesn't seem to be individual clustering or temporal autocorrelation in the residuals anymore.

glmmTMB was not calculating Pearson residuals correctly for the beta regression with random effects (so we calculated it ourselves) - specifically they are using the equation for the variance function:

*var(X) = (1-mu)*mu*phi^2*

The documentation states that they use the same function as the Betareg package for beta distributions, specifically:

var(X) = mu(1-mu)/(1+phi)*

Next, residuals vs linear predictor

```
horiz <- function(x) 0
data$lp <- predict(M_best,type="link") #linear predictor ?
p_basic <- data %>%
    ggplot() +
    geom_point(aes(x = lp, y = E, color = interaction(diel,feed))) +
```

```
    geom_function(fun = horiz) +
    ylim(-4.1, 4) +
    xlim(-3,2.5)

p_basic
```

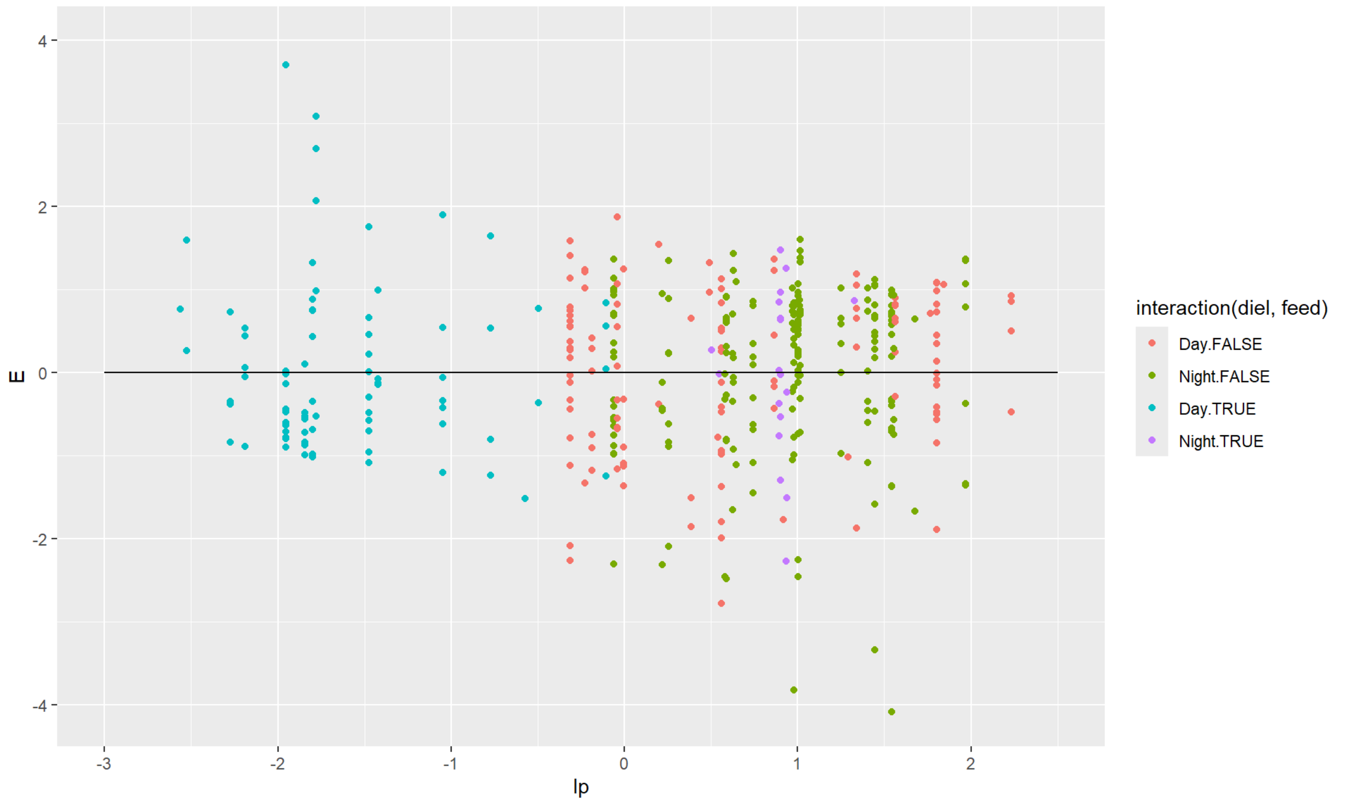

Looks good, but there are still some periods that have residual patterns:

Day feeding periods with high residuals and non feeding periods with low residuals. We will explore the reason why later.

Next, residuals against each of the fixed factors individually.

```
par(mfrow=c(2,2),mar=c(2,2,2,2))
plot(data$E~data$reg)
boxplot(data$E~data$feed)
plot(data$E~data$diel)
plot(data$E~data$id)
```

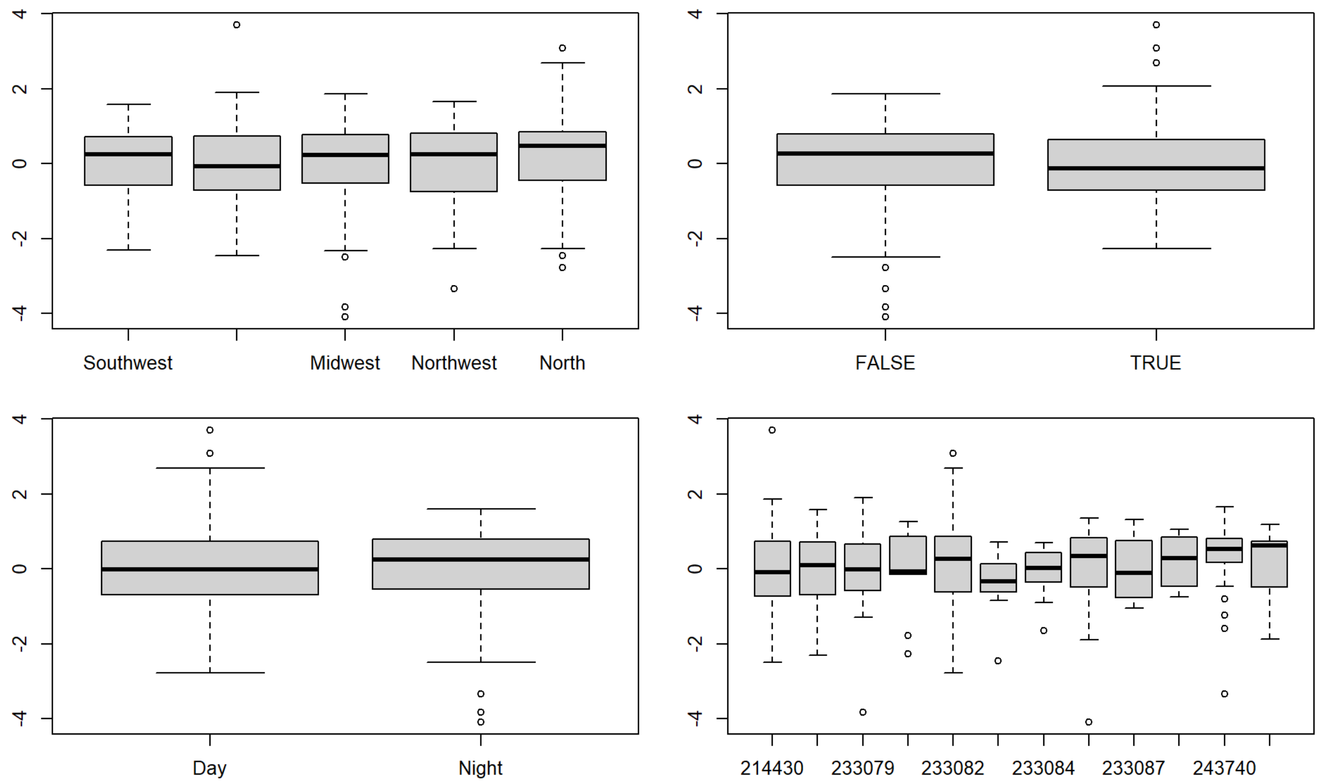


```
par(mfrow=c(1,1),mar=c(5,4,4,2))
```

They look good, some minor heteroskedasticity across the regions and whale ID's. North region is a bit more variable, while one whale had greater variation, and a couple others had less variation.

Next, the temporal and spatial autocorrelation.

```
mydata<-data.frame(data$E,data$lon,data$lat)
sp::coordinates(mydata) <- c("data.lon","data.lat")
sp::bubble(mydata,"data.E",col=c("black","grey"),main="Normalized residuals",
           xlab="X-coordinates",ylab="Y-coordinates")
```

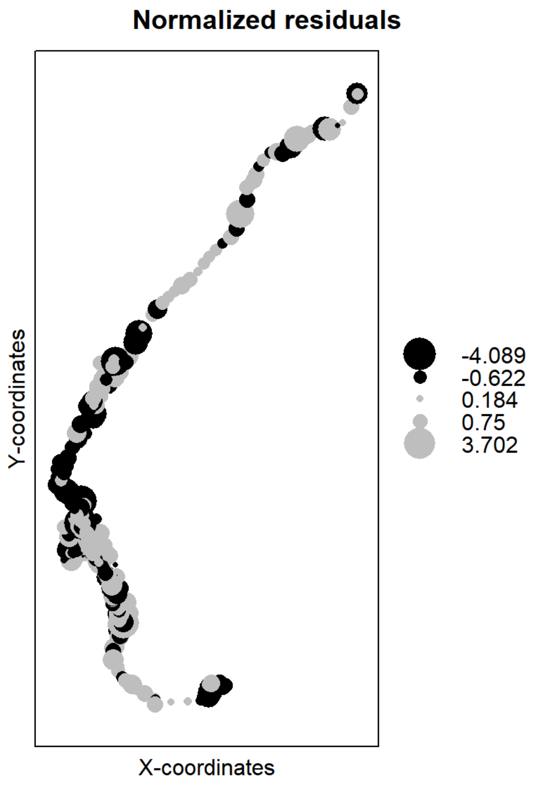

Looks good. Minor positive clusters in NW and the SW corners, and negative in the westernmost point. The positive clusters are in locations with just one whale.

These may indicate finer scale geographic or individual patterns that we are not accounting for. We will explore why later.

```
W <- unique(data$id)
par(mfrow=c(2,3),mar=c(2,2,4,1))
for (i in 1:length(W)){
  acf(data$E[data$id==W[i]],main=W[i],xlab="",ylab="",lag.max=4)
}
```

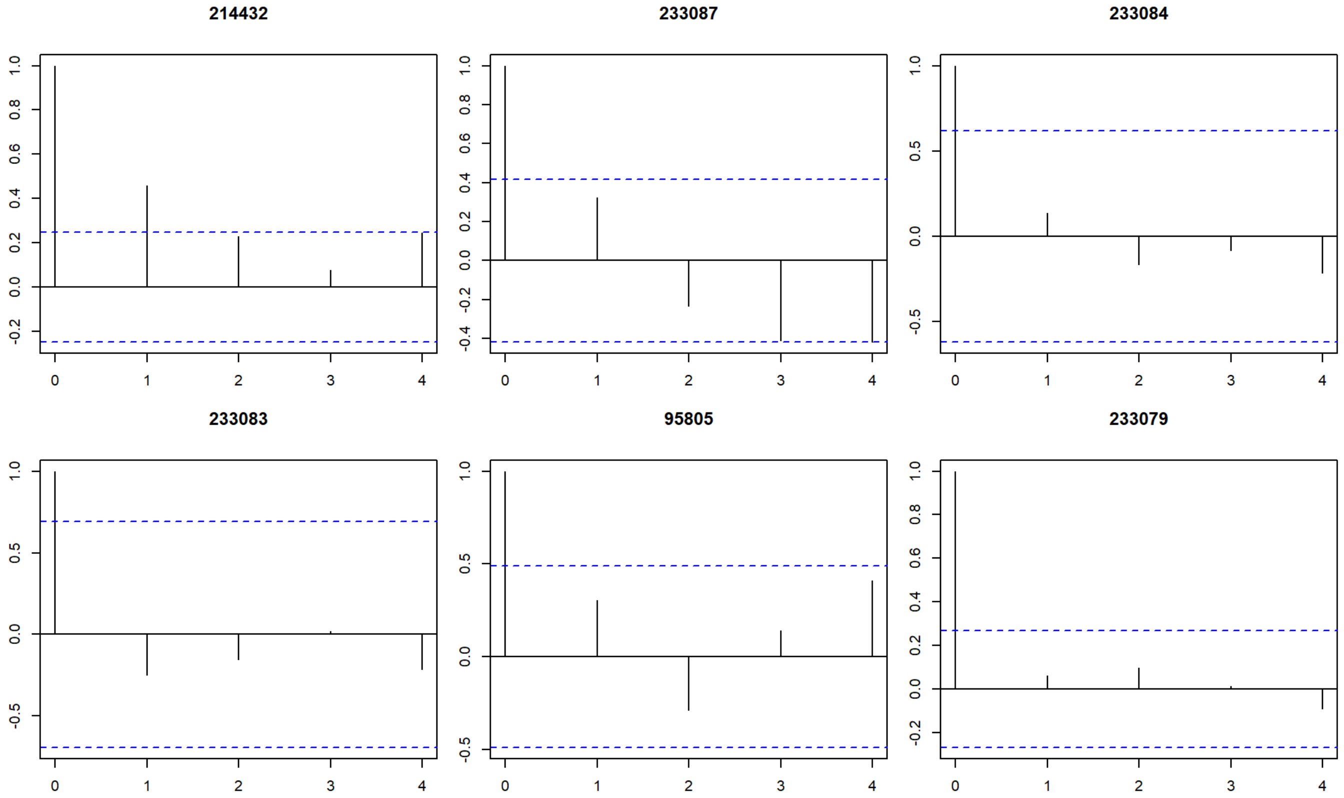

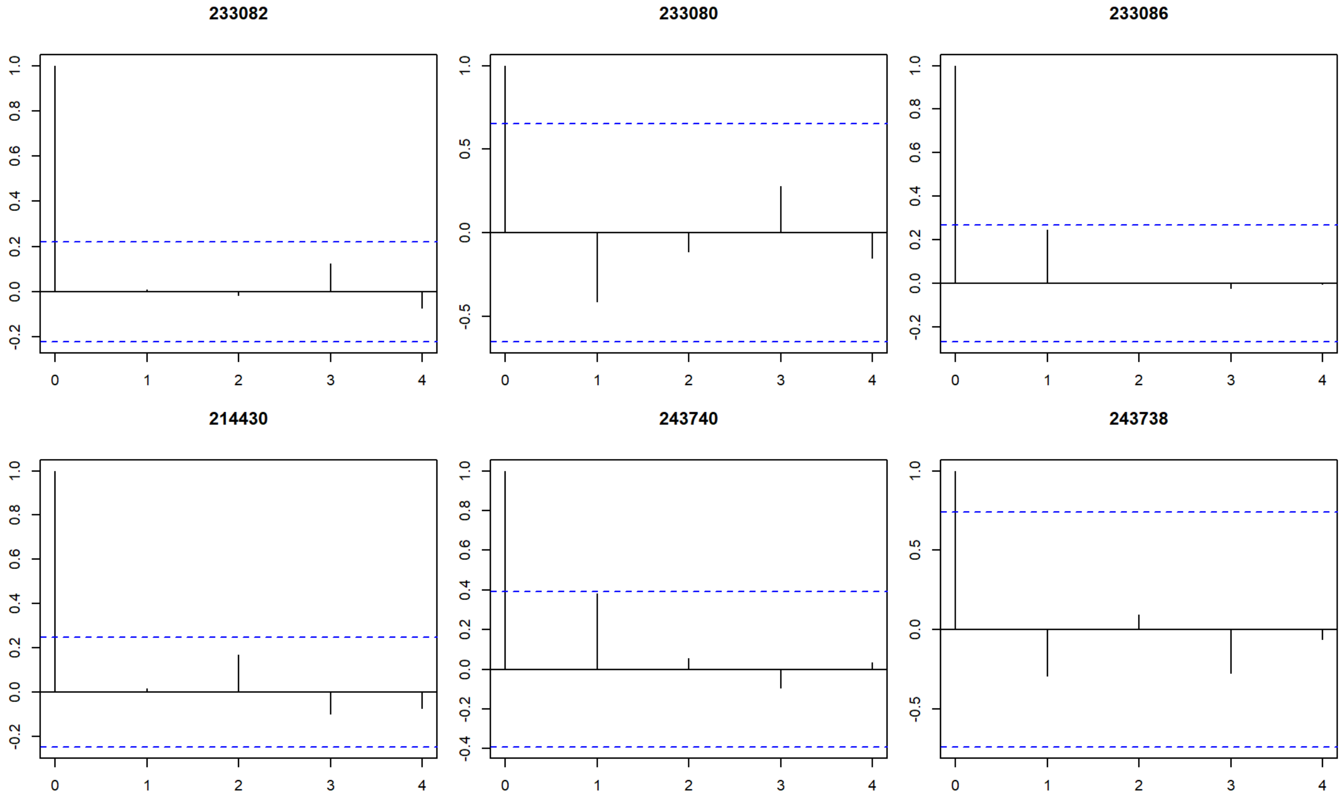


```
par(mfrow=c(1,1),mar=c(5,4,4,2))
```

214432, 233086, and 243740 have borderline AR1 correlation structures. All other animals do not have temporal autocorrelation, so we don’t consider this to be an issue.

glmmtmb allows an AR1 correlation structure, which was tested, but it didn’t converge.

```
data$fday <- factor(data$day * 2)

#M_ar1 <- glmmTMB(pt23_jitter ~ diel * feed + reg + ar1(fday+0|id),
#                   dispformula = ~ diel * feed + reg + (feed|id),
#                   family="beta_family",
#                   data = data)
```

Unknown variable data exploration

We suspect that any remaining residual patterns were related to whale 233082, given its contribution to the deviance from homoskedasticity.

First lets plot predicted values for each factor and whale combination on violins of raw values across the fixed factors.

```
data$re <- predict(M_best,type="response") #raw vs fitted
data$Whale <- data$id
data$Period <- data$diel
data$paF <- as.factor(ifelse(data$feed==TRUE,"Feeding","Non-feeding"))

data %>%     ggplot() +
  geom_violin(aes(x = Period, y = pt23, fill = Period), alpha = 0.4,show.legend = FALSE) +
  geom_point(aes(x = Period, y = re, col = Whale)) +
  facet_grid(rows = vars(paF),cols = vars(reg)) +
  labs(x="Diel period",y="Proportion of time available for singing") +
  guides(col = guide_legend(position="top",ncol=12)) +
  theme_bw() +
  theme(legend.key.width=unit(1,"null"),
        legend.key.height=unit(0.5,"null"),
        panel.grid.major.x = element_blank(),
        panel.grid.minor.y = element_blank())
```

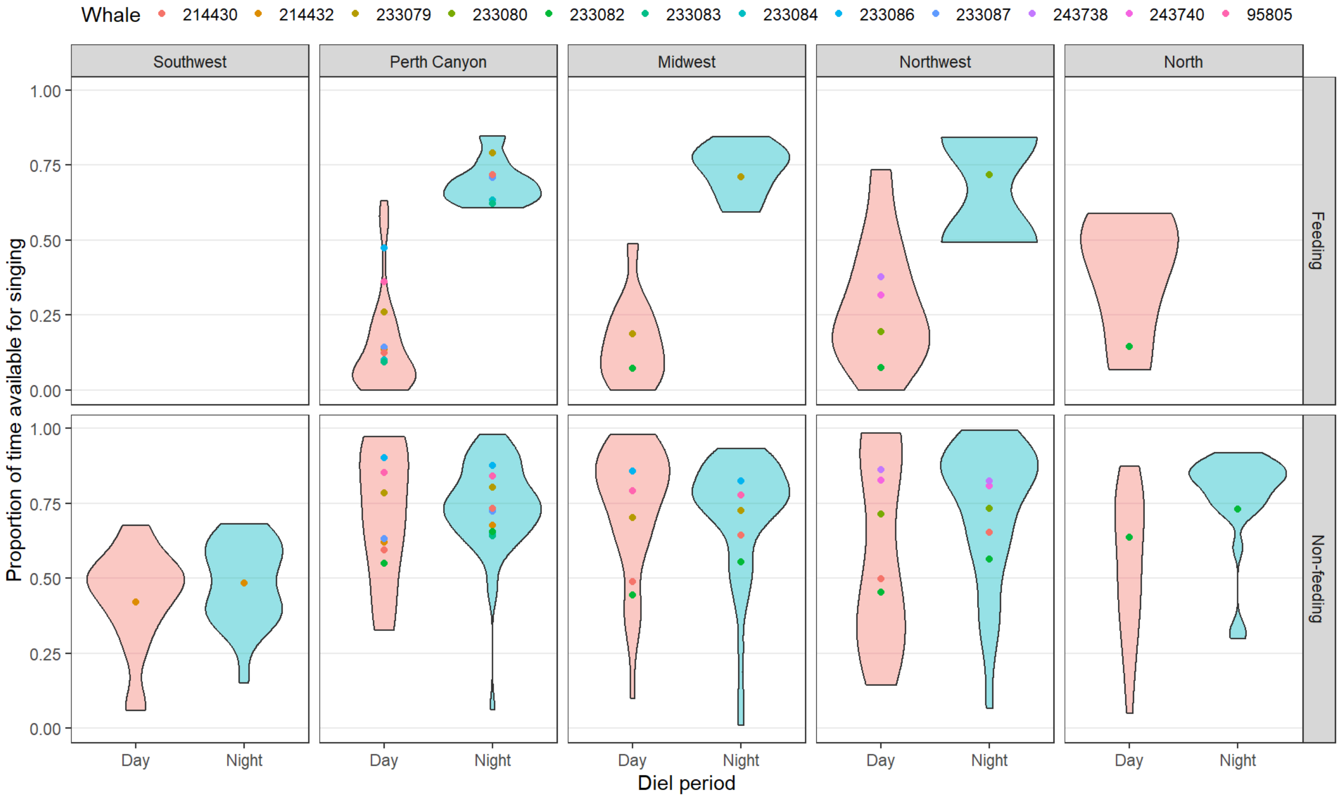

The predicted responses fit pretty well to raw values everywhere except the North region. Whale 233082 seems to be a culprit for the lower values of TAS in non-feeding periods, so if we remove that animal, and perhaps 214430 too as it has low predicted TAS as well. What does that look like?

```
delse <- subset(data,id != 233082 & id != 214430)

#this bit is to keep point colours consistent with previous plots
gg_color_hue <- function(n) {
  hues = seq(15, 375, length = n + 1)
  hcl(h = hues, l = 65, c = 100)[1:n]
}
Cl <- gg_color_hue(12)
names(Cl) <- levels(data$Whale)
colScale <- scale_colour_manual(name="Whale",values=Cl)

delse %>%     ggplot() +
  geom_violin(aes(x = Period, y = pt23, fill = Period), alpha = 0.4,show.legend = FALSE) +
  geom_point(aes(x = Period, y = re, col = Whale)) +
  facet_grid(rows = vars(paF),cols = vars(reg),drop=FALSE) +
  labs(x="Diel period",y="Proportion of time available for singing") +
  guides(col = guide_legend(position="top",ncol=12)) +
  theme_bw() +
  theme(legend.key.width=unit(1,"null"),
        legend.key.height=unit(0.5,"null"),
        panel.grid.major.x = element_blank(),
        panel.grid.minor.y = element_blank()) +
  colScale
```

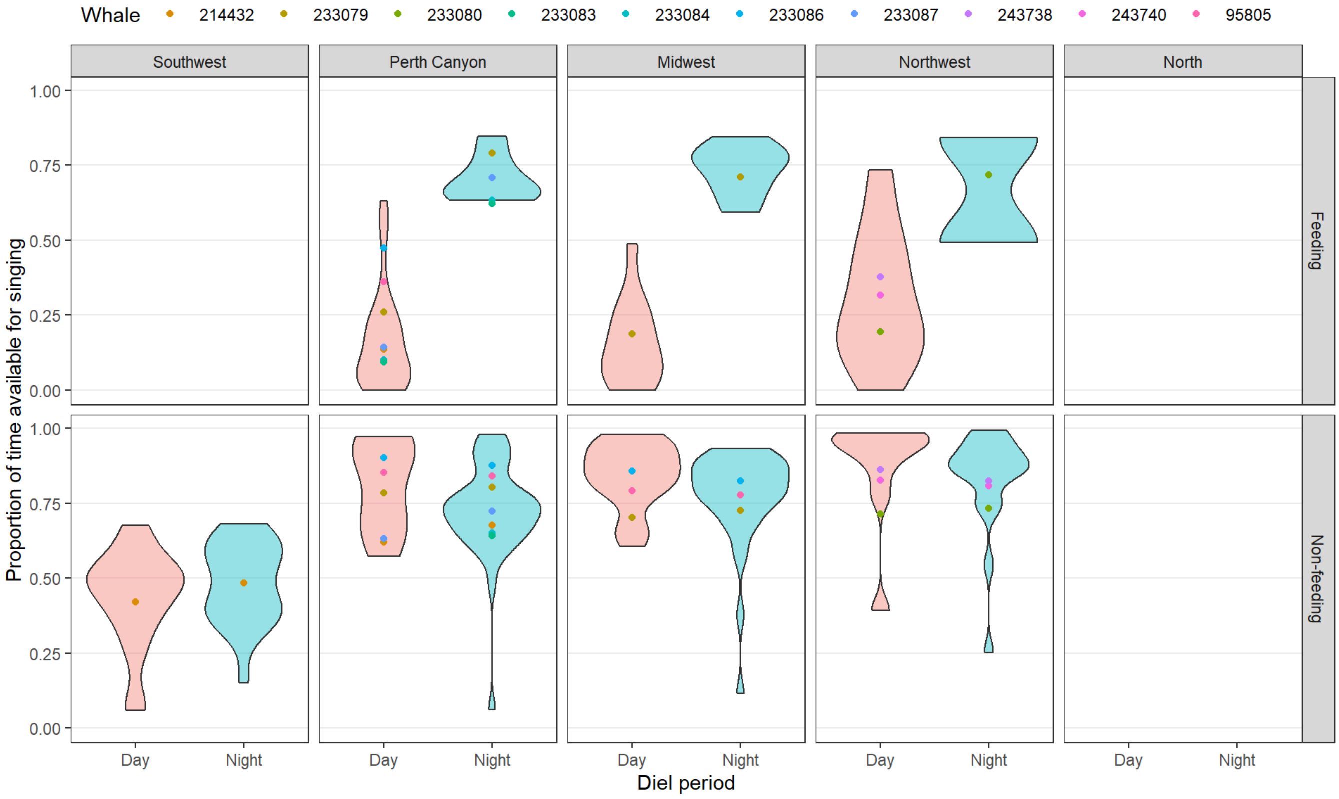

Most of the raw data from those low tails in non feeding are gone, but there are a still a small handful of periods with low TAS. The raw TAS distributed for the Southwest non-feed region is similar to where we have lost that lower tail.

```
dexp <- subset(data,id == 233082 | id == 214430)




#then plot
dexp %>%     ggplot() +
  geom_violin(aes(x = Period, y = pt23, fill = Period), alpha = 0.4,show.legend = FALSE) +
  geom_point(aes(x = Period, y = re, col = Whale)) +
  facet_grid(rows = vars(paF),cols = vars(reg),drop=FALSE) +
  labs(x="Diel period",y="Proportion of time available for singing") +
  guides(col = guide_legend(position="top",ncol=12)) +
  theme_bw() +
  theme(legend.key.width=unit(1,"null"),
        legend.key.height=unit(0.5,"null"),
        panel.grid.major.x = element_blank(),
        panel.grid.minor.y = element_blank()) +
  colScale
```

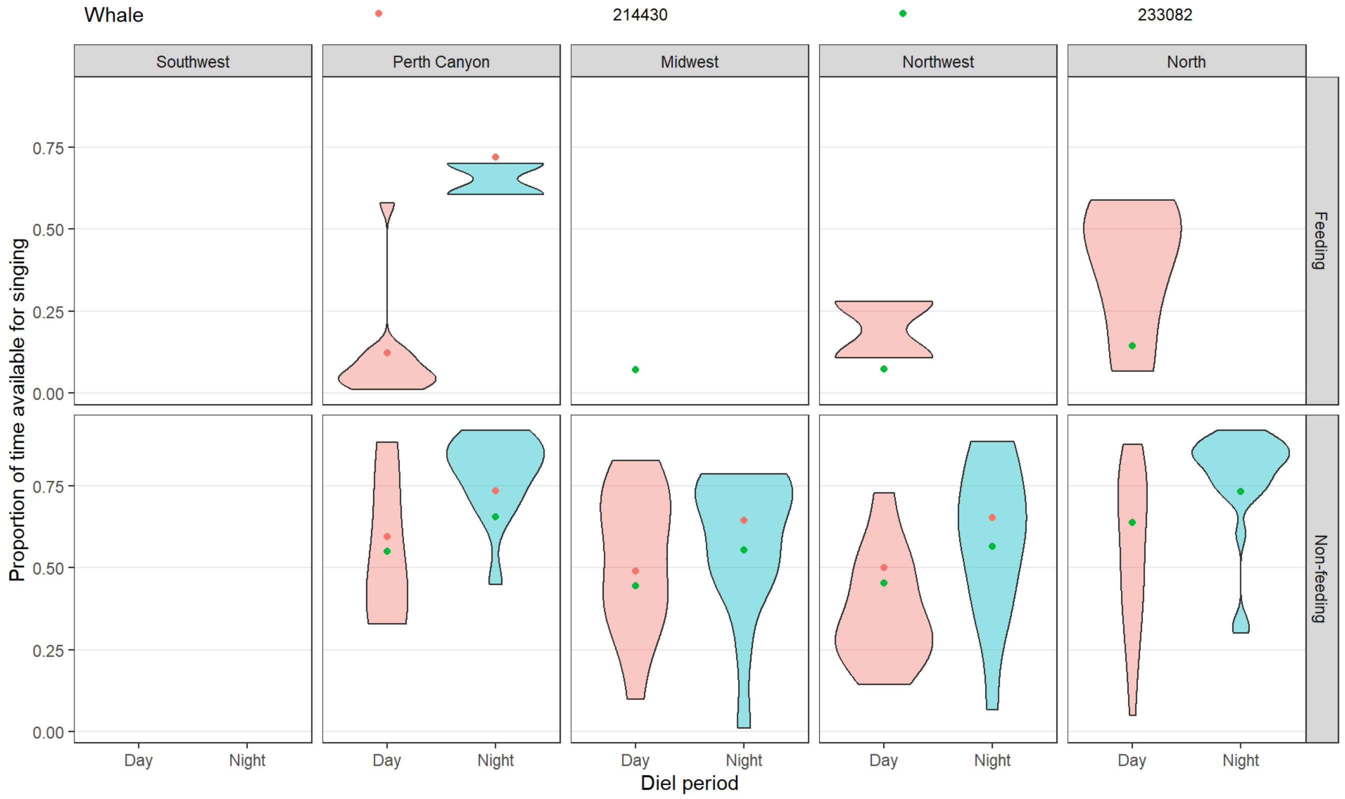

For only the removed whales, the predicted TAS fits their raw distributions well (except feeding in the North).

To explore what this behaviour was that affected TAS for these whales, will need to review the behavioural time series for these individuals.

Exploring a region interaction effect with feeding

By removing the SC region, we can explore region:diel and region:feed interaction effects, especially to account for the poor feeding fit in the N region. Starting with the best model expression for the full dataset, we then proceed on a forward stepwise selection process

```
D2 <- subset(data,reg != "Southwest")

M9a <- glmmTMB(pt23_jitter ~ diel * feed + reg + (diel|id),
                  dispformula = ~ diel * feed + reg + (feed|id),
                  family="beta_family",
                  data = D2)
M9b <- glmmTMB(pt23_jitter ~ diel * feed + reg *feed + (diel|id),
                dispformula = ~ diel * feed + reg + (feed|id),
                family="beta_family",
                data = D2)
M9c <- glmmTMB(pt23_jitter ~ diel * feed + reg + (diel|id),
                dispformula = ~ diel * feed + reg *feed + (feed|id),
                family="beta_family",
                data = D2)
M9d <- glmmTMB(pt23_jitter ~ diel * feed + reg * diel + (diel|id),
               dispformula = ~ diel * feed + reg + (feed|id),
               family="beta_family",
               data = D2)
```

```
M9e <- glmmTMB(pt23_jitter ~ diel * feed + reg + (diel|id),
               dispformula = ~ diel * feed + reg * diel + (feed|id),
               family="beta_family",
               data = D2)
A <- AIC(M9a,M9b,M9c,M9d,M9e)
print.data.frame(A[,])
```

```
    df       AIC
M9a 20 -396.1407
M9b 23 -402.4381
M9c 23 -394.6033
M9d 23 -393.8421
M9e 23 -396.5942
```

select m9b, which includes a region*feed interaction on the conditional model.

```
M9f <- glmmTMB(pt23_jitter ~ diel * feed + reg *feed + (diel|id),
                dispformula = ~ diel * feed + reg *feed + (feed|id),
                family="beta_family",
                data = D2)
M9g <- glmmTMB(pt23_jitter ~ diel * feed * reg + (diel|id),
               dispformula = ~ diel * feed + reg + (feed|id),
               family="beta_family",
               data = D2)
A <- AIC(M9b,M9f,M9g)
print.data.frame(A[,])
```

```
    df       AIC
M9b 23 -402.4381
M9f 26 -399.1396
M9g 28 -401.5144
```

Compared to the reference model (M9A), M9B and M9G are (similar) improvements. M9G is a 3-way interaction on the conditional model, and M9B just adds a reg*feed interaction. M9B was selected for the lower AIC.

```
summary(M9b)
```

```
 Family: beta  ( logit )
Formula:          pt23_jitter ~ diel * feed + reg * feed + (diel | id)
Dispersion:                   ~diel * feed + reg + (feed | id)
Data: D2

     AIC      BIC   logLik deviance df.resid
  -402.4   -312.9    224.2   -448.4      340

Random effects:

Conditional model:
 Groups Name        Variance Std.Dev. Corr
 id     (Intercept) 0.7044   0.8393
        dielNight   0.1350   0.3674   -0.94
Number of obs: 363, groups:  id, 12
```

```
Dispersion model:
 Groups Name        Variance Std.Dev. Corr
 id     (Intercept) 0.7520   0.8672
        feedTRUE    0.9317   0.9653   -0.91
Number of obs: 363, groups:  id, 12

Conditional model:
                      Estimate Std. Error z value Pr(>|z|)
(Intercept)             1.0948     0.2841   3.853 0.000116 ***
dielNight               0.1645     0.1628   1.010 0.312368
feedTRUE               -2.5522     0.1867 -13.673  < 2e-16 ***
regMidwest             -0.4842     0.1278  -3.789 0.000152 ***
regNorthwest           -0.4310     0.1924  -2.240 0.025087 *
regNorth                0.2222     0.2445   0.909 0.363464
dielNight:feedTRUE      2.3860     0.2027  11.768  < 2e-16 ***
feedTRUE:regMidwest     0.1988     0.2295   0.866 0.386264
feedTRUE:regNorthwest   0.2873     0.3848   0.747 0.455166
feedTRUE:regNorth       1.5839     0.4048   3.913 9.12e-05 ***
---
Signif. codes:  0 '***' 0.001 '**' 0.01 '*' 0.05 '.' 0.1 ' ' 1

Dispersion model:
                   Estimate Std. Error z value Pr(>|z|)
(Intercept)         2.41956    0.35460   6.823 8.89e-12 ***
dielNight           0.16357    0.20148   0.812  0.41690
feedTRUE           -0.59417    0.39391  -1.508  0.13146
regMidwest         -0.49441    0.22500  -2.197  0.02799 *
regNorthwest       -0.55020    0.32242  -1.706  0.08792 .
regNorth            0.01007    0.35097   0.029  0.97711
dielNight:feedTRUE  1.53750    0.46777   3.287  0.00101 **
---
Signif. codes:  0 '***' 0.001 '**' 0.01 '*' 0.05 '.' 0.1 ' ' 1
```

Of the levels of the feed:diel interaction, only the feedTRUE:regNORTH term was significant. So there is a missing interaction effect here in our best model for the full dataset.

If we remove the North region as well and run all the selection process again, does the region:feed or region:diel interaction remain important (given only a single whale contributed data for the North region)?

```
D3 <- subset(data,reg != "Southwest" & reg != "North")

M10a <- glmmTMB(pt23_jitter ~ diel * feed + reg + (diel|id),
               dispformula = ~ diel * feed + reg + (feed|id),
               family="beta_family",
               data = D3)
M10b <- glmmTMB(pt23_jitter ~ diel * feed + reg *feed + (diel|id),
               dispformula = ~ diel * feed + reg + (feed|id),
               family="beta_family",
               data = D3)
M10c <- glmmTMB(pt23_jitter ~ diel * feed + reg + (diel|id),
               dispformula = ~ diel * feed + reg *feed + (feed|id),
```

```
               family="beta_family",
               data = D3)
M10d <- glmmTMB(pt23_jitter ~ diel * feed + reg * diel + (diel|id),
               dispformula = ~ diel * feed + reg + (feed|id),
               family="beta_family",
               data = D3)
M10e <- glmmTMB(pt23_jitter ~ diel * feed + reg + (diel|id),
               dispformula = ~ diel * feed + reg * diel + (feed|id),
               family="beta_family",
               data = D3)
A <- AIC(M10a,M10b,M10c,M10d,M10e)
print.data.frame(A[,])
```

```
     df       AIC
M10a 18 -382.6867
M10b 20 -379.9315
M10c 20 -380.0488
M10d 20 -380.1737
M10e 20 -379.6352
```

The full dataset model expression remains the best for this subset, i.e., the feed:region interaction was not important for Perth Canyon, Midwest and Northwest.

```
summary(M10a)
```

```
 Family: beta  ( logit )
Formula:          pt23_jitter ~ diel * feed + reg + (diel | id)
Dispersion:                   ~diel * feed + reg + (feed | id)
Data: D3

     AIC      BIC   logLik deviance df.resid
  -382.7   -315.1    209.3   -418.7      297

Random effects:

Conditional model:
 Groups Name        Variance Std.Dev. Corr
 id     (Intercept) 0.7367   0.8583
        dielNight   0.1413   0.3759   -0.89
Number of obs: 315, groups:  id, 12

Dispersion model:
 Groups Name        Variance Std.Dev. Corr
 id     (Intercept) 0.7558   0.8694
        feedTRUE    1.2008   1.0958   -0.92
Number of obs: 315, groups:  id, 12

Conditional model:
                   Estimate Std. Error z value Pr(>|z|)
(Intercept)         1.18292    0.28659   4.128 3.67e-05 ***
dielNight           0.05311    0.16603   0.320 0.749084
feedTRUE           -2.54238    0.17487 -14.539  < 2e-16 ***
regMidwest         -0.44239    0.11550  -3.830 0.000128 ***
```

```
regNorthwest       -0.43977    0.19513  -2.254 0.024215 *
dielNight:feedTRUE  2.47604    0.20700  11.962  < 2e-16 ***
---
Signif. codes:  0 '***' 0.001 '**' 0.01 '*' 0.05 '.' 0.1 ' ' 1

Dispersion model:
                   Estimate Std. Error z value Pr(>|z|)
(Intercept)         2.57960    0.35697   7.226 4.96e-13 ***
dielNight          -0.01915    0.21912  -0.087 0.930369
feedTRUE           -0.70925    0.42864  -1.655 0.097993 .
regMidwest         -0.48783    0.22168  -2.201 0.027761 *
regNorthwest       -0.58596    0.31223  -1.877 0.060563 .
dielNight:feedTRUE  1.62801    0.48739   3.340 0.000837 ***
---
Signif. codes:  0 '***' 0.001 '**' 0.01 '*' 0.05 '.' 0.1 ' ' 1
```

Plot predictions with standard error from the full optimal model

```
vars <- data[,c(2:4)]
vars2 <- vars[!duplicated(vars),]
p <- predict(M_best,newdata = vars2,type="response",re.form=NA,se.fit=TRUE)
vars2$p <- p$fit
vars2$se <- p$se.fit
head(vars2)
```

```
    diel          reg  feed         p         se
1    Day Perth Canyon  TRUE 0.2152948 0.04799817
2  Night Perth Canyon FALSE 0.7739540 0.03140216
17   Day Perth Canyon FALSE 0.7396305 0.05323648
21   Day    Southwest FALSE 0.5591159 0.07413136
22 Night    Southwest FALSE 0.6045117 0.05354995
64 Night Perth Canyon  TRUE 0.7602030 0.03664188
```

```
vars2 %>%     ggplot() +
  geom_jitter(aes(x = reg, y = p, col = interaction(diel,feed)), width = 0.05) +
  geom_errorbar(aes(x = reg, y = p,
                  ymin = p-se*1.96,ymax = p+se*1.96,
                  col = interaction(diel,feed)))
```

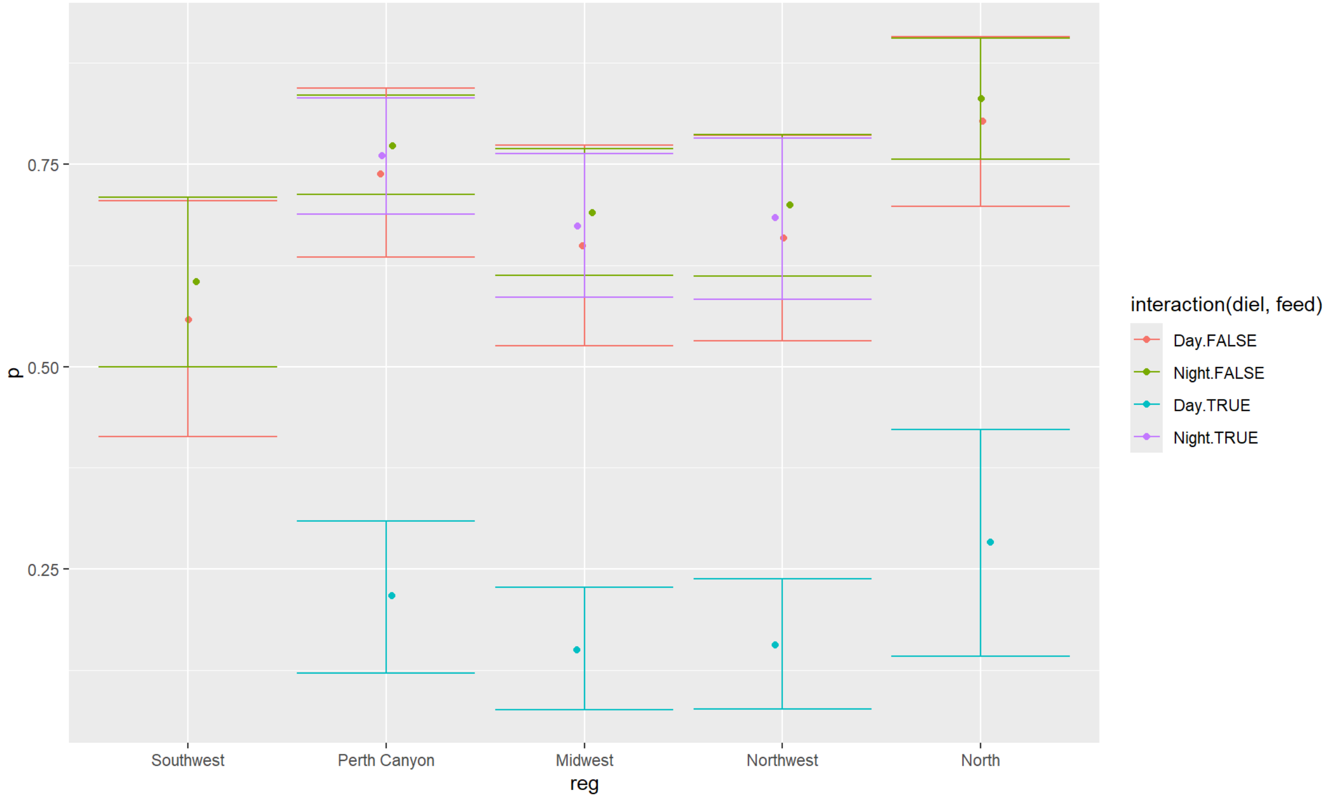
0.75
0.50
0.25
p
Southwest
Perth Canyon
Midwest
Northwest
North
reg
interaction(diel, feed)
Day.FALSE
Night.FALSE
Day.TRUE
Night.TRUE
