# Supplementary material for: Pygmy blue whale feeding drives greater nocturnal than diurnal song production
Source: R Soc Open Sci. 2026 Oct 28;13(10):260954. doi: 10.1098/rsos.260954 (PMC13714375; doi:10.1098/rsos.260954)
Supplement: Supplementary file 1 [file rsos.260954.zip › rsos.260954/rsos260954_si_004.pdf]

# Pygmy blue whale feeding drives greater nocturnal than diurnal song production

Andrew M. Davenport, Michele Thums, K. Curt S. Jenner, Micheline-Nicole M. Jenner, Luciana C. Ferreira, Christine Erbe, Ryan Leadbetter, Robert D. McCauley

Royal Society Open Science (DOI: 10.1098/rsos.260954)

## Supplementary figures of acoustic datasets

Periodicity of pygmy blue whale calling rate (Unit 2 song calls per hour) by hour of day and date for three regions: Perth Canyon, Northwest, and North. Black + symbols are the circular mean calling hour for each 24 h period and the horizontal black lines indicate the time of sunset and sunrise for each day. Note that the colour scales are different between the axes to best distinguish within dataset periodicities rather than compare intensity between the sites. The metadata table for the datasets is in the supplementary methods and results. The location of all sets can be seen in the map below.

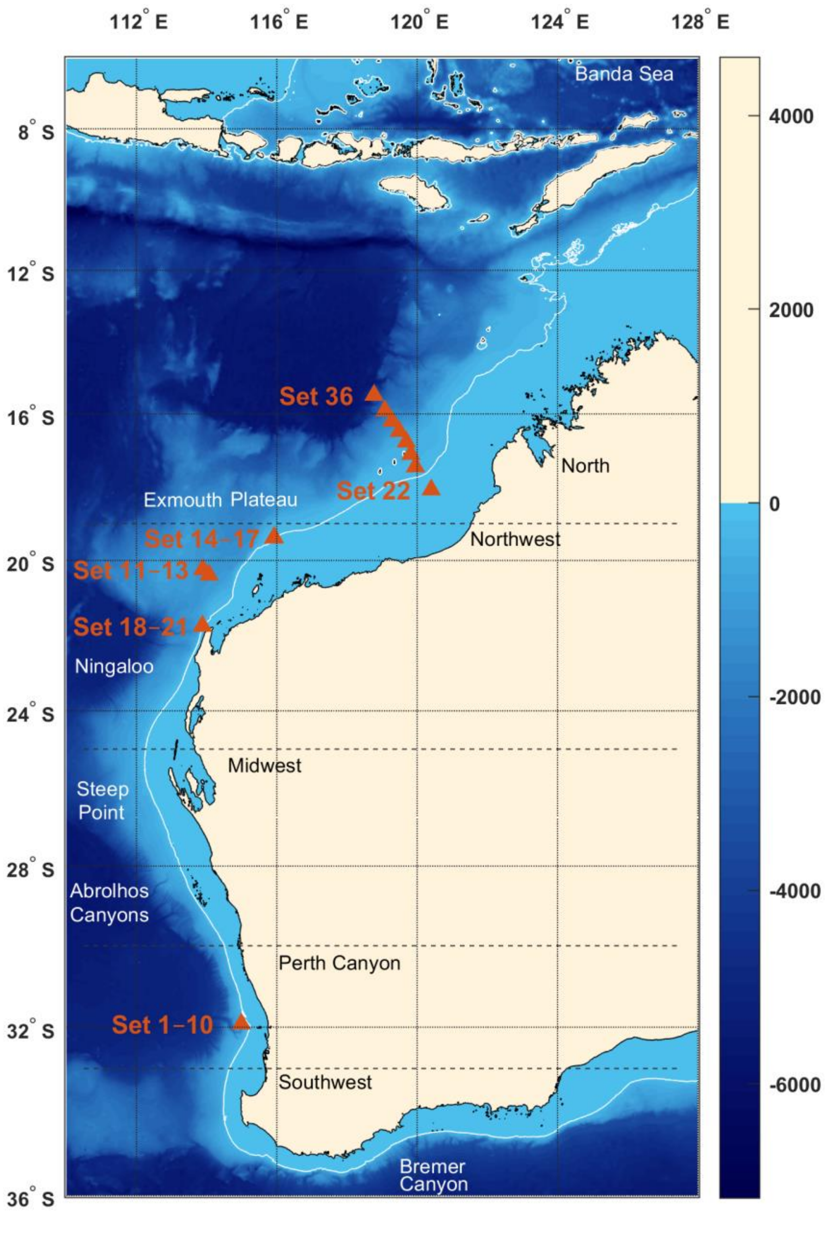

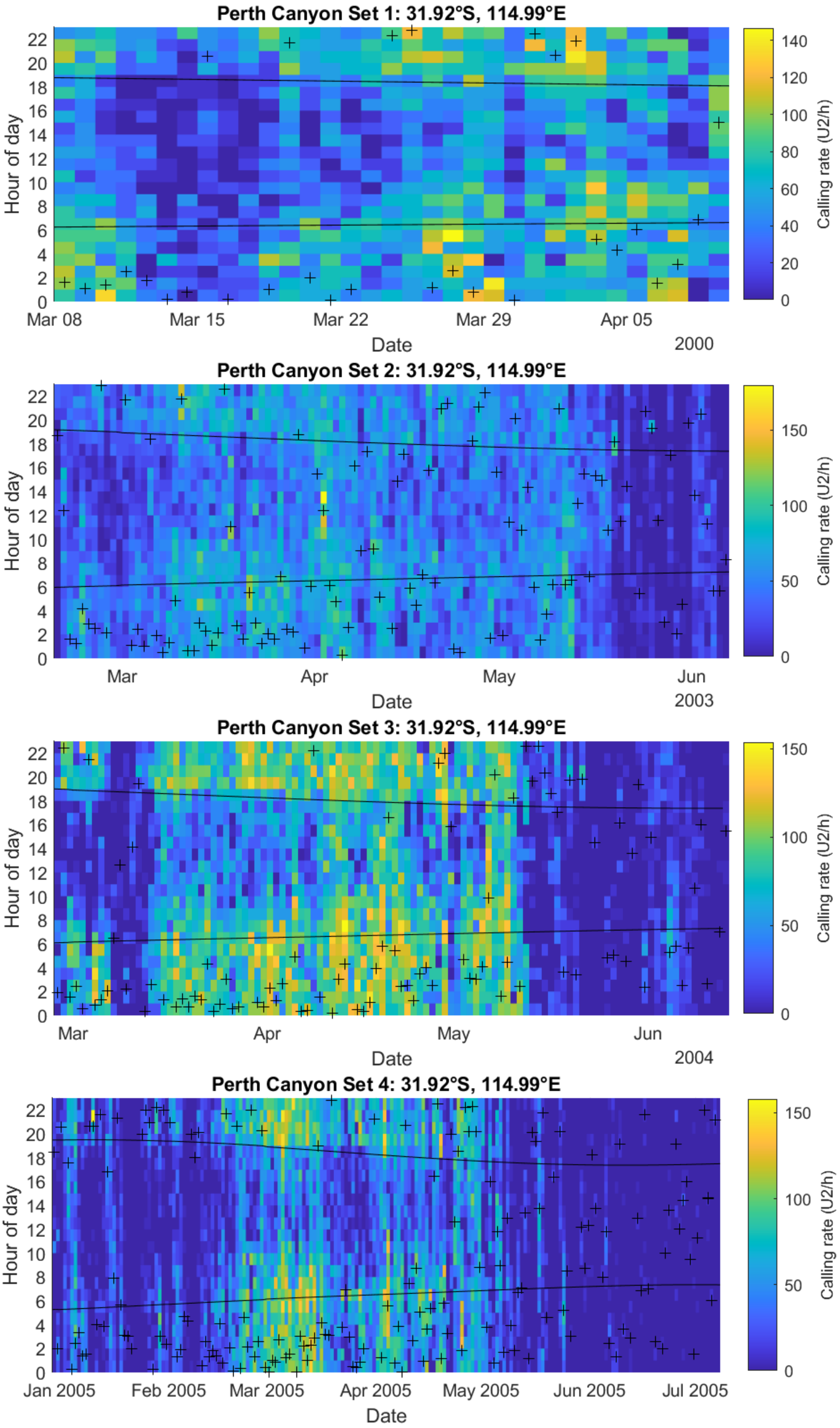

Perth Canyon Set 1: 31.92°S, 114.99°E
Hour of day
Date
Mar 08
Mar 15
Mar 22
Mar 29
Apr 05
2000
Calling rate (U2/h)
Perth Canyon Set 2: 31.92°S, 114.99°E
Hour of day
Date
Mar
Apr
May
Jun
2003
Calling rate (U2/h)
Perth Canyon Set 3: 31.92°S, 114.99°E
Hour of day
Date
Mar
Apr
May
Jun
2004
Calling rate (U2/h)
Perth Canyon Set 4: 31.92°S, 114.99°E
Hour of day
Date
Jan 2005
Feb 2005
Mar 2005
Apr 2005
May 2005
Jun 2005
Jul 2005
Calling rate (U2/h)

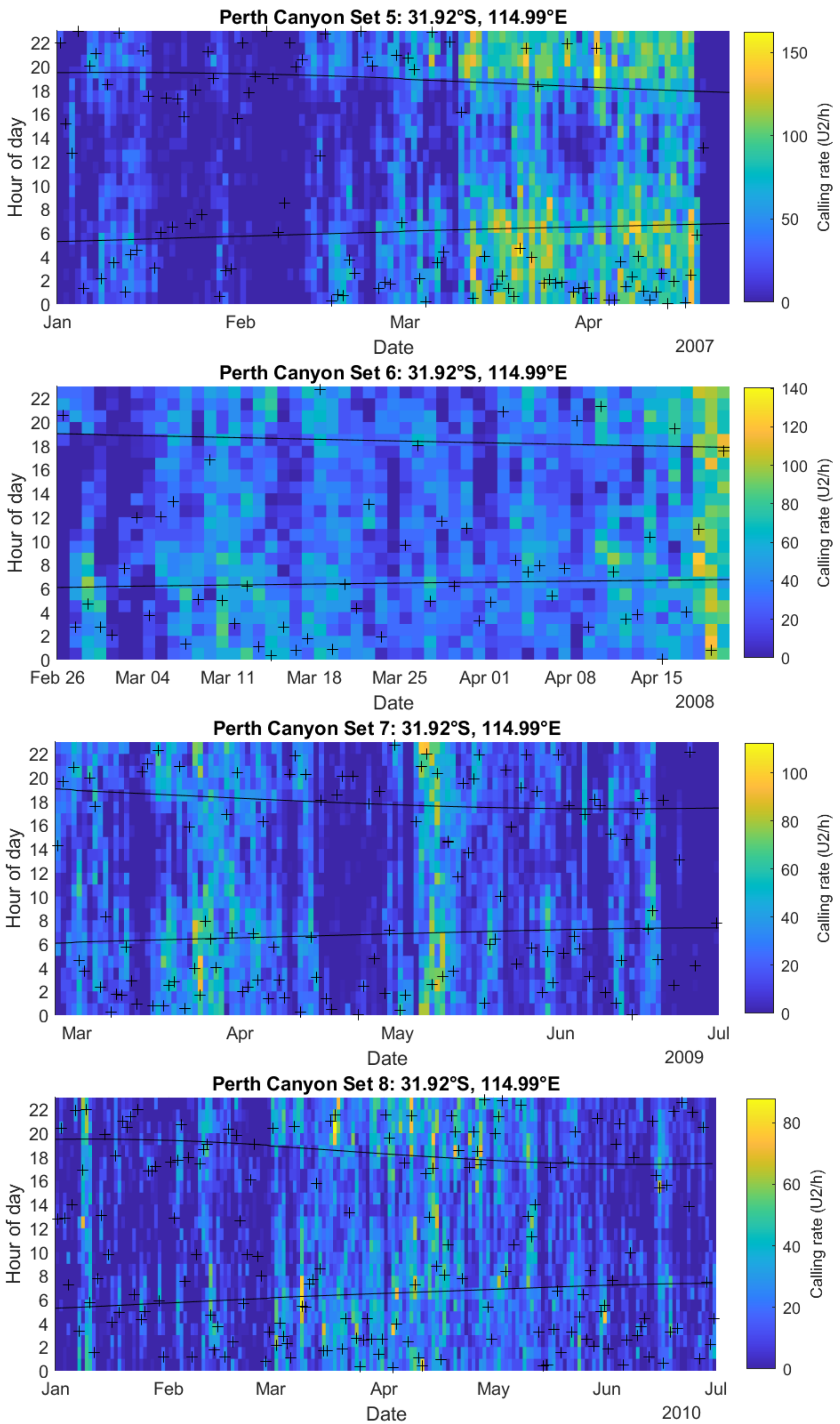

Perth Canyon Set 5: 31.92°S, 114.99°E
Hour of day
Date
Jan
Feb
Mar
Apr
2007
Calling rate (U2/h)
Perth Canyon Set 6: 31.92°S, 114.99°E
Hour of day
Date
Feb 26
Mar 04
Mar 11
Mar 18
Mar 25
Apr 01
Apr 08
Apr 15
2008
Calling rate (U2/h)
Perth Canyon Set 7: 31.92°S, 114.99°E
Hour of day
Date
Mar
Apr
May
Jun
Jul
2009
Calling rate (U2/h)
Perth Canyon Set 8: 31.92°S, 114.99°E
Hour of day
Date
Jan
Feb
Mar
Apr
May
Jun
Jul
2010
Calling rate (U2/h)

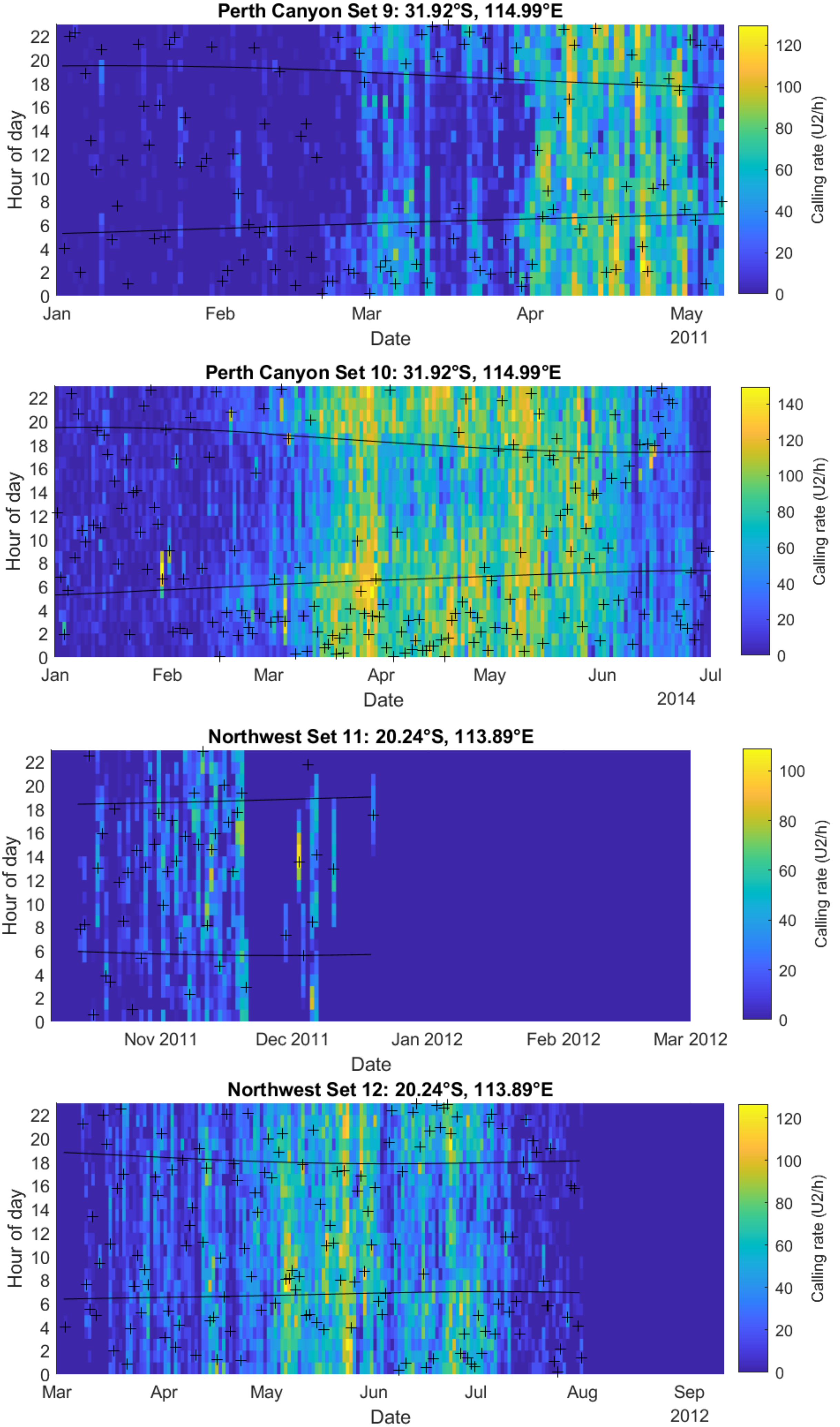
Perth Canyon Set 9: 31.92°S, 114.99°E
Hour of day
Date
Jan
Feb
Mar
Apr
May
2011
Calling rate (U2/h)
Perth Canyon Set 10: 31.92°S, 114.99°E
Hour of day
Date
Jan
Feb
Mar
Apr
May
Jun
Jul
2014
Calling rate (U2/h)
Northwest Set 11: 20.24°S, 113.89°E
Hour of day
Date
Nov 2011
Dec 2011
Jan 2012
Feb 2012
Mar 2012
Calling rate (U2/h)
Northwest Set 12: 20.24°S, 113.89°E
Hour of day
Date
Mar
Apr
May
Jun
Jul
Aug
Sep
2012
Calling rate (U2/h)

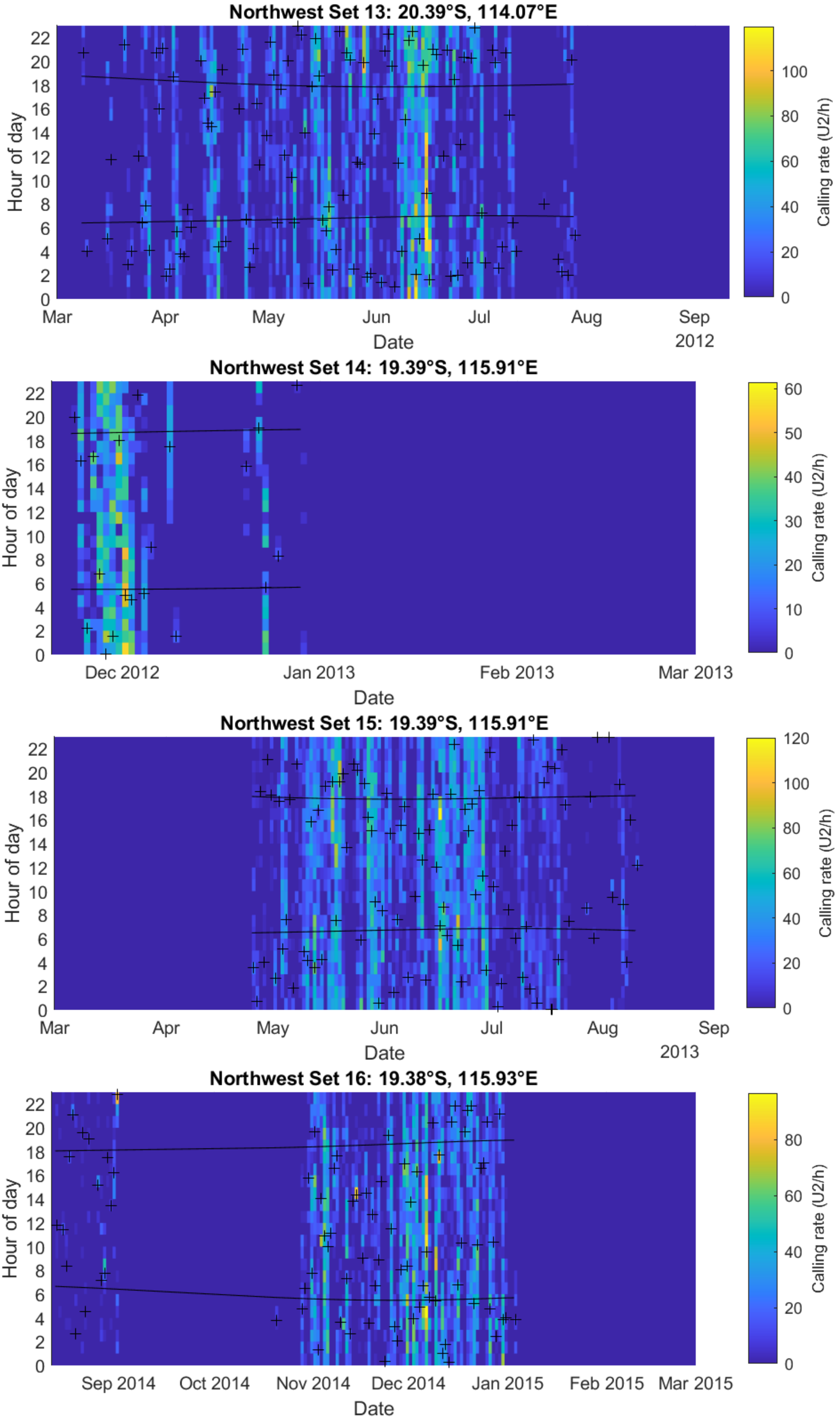
Northwest Set 13: 20.39°S, 114.07°E
Hour of day
Date
Mar
Apr
May
Jun
Jul
Aug
Sep
2012
Calling rate (U2/h)
Northwest Set 14: 19.39°S, 115.91°E
Dec 2012
Jan 2013
Feb 2013
Mar 2013
Northwest Set 15: 19.39°S, 115.91°E
2013
Northwest Set 16: 19.38°S, 115.93°E
Sep 2014
Oct 2014
Nov 2014
Dec 2014
Jan 2015
Feb 2015
Mar 2015

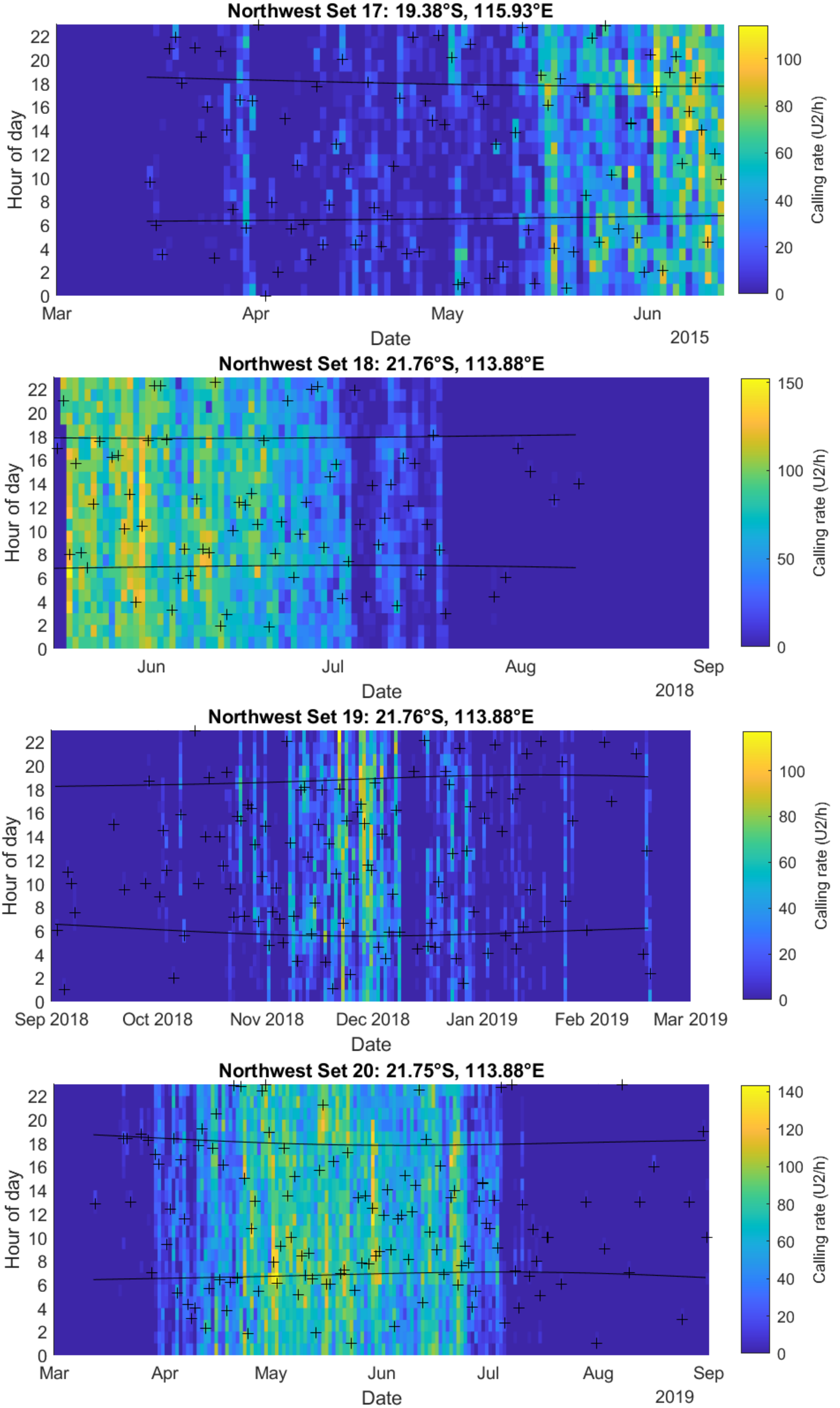
Northwest Set 17: 19.38°S, 115.93°E
Hour of day
Date
Mar
Apr
May
Jun
2015
Calling rate (U2/h)
Northwest Set 18: 21.76°S, 113.88°E
Hour of day
Date
Jun
Jul
Aug
Sep
2018
Calling rate (U2/h)
Northwest Set 19: 21.76°S, 113.88°E
Hour of day
Date
Sep 2018
Oct 2018
Nov 2018
Dec 2018
Jan 2019
Feb 2019
Mar 2019
Calling rate (U2/h)
Northwest Set 20: 21.75°S, 113.88°E
Hour of day
Date
Mar
Apr
May
Jun
Jul
Aug
Sep
2019
Calling rate (U2/h)

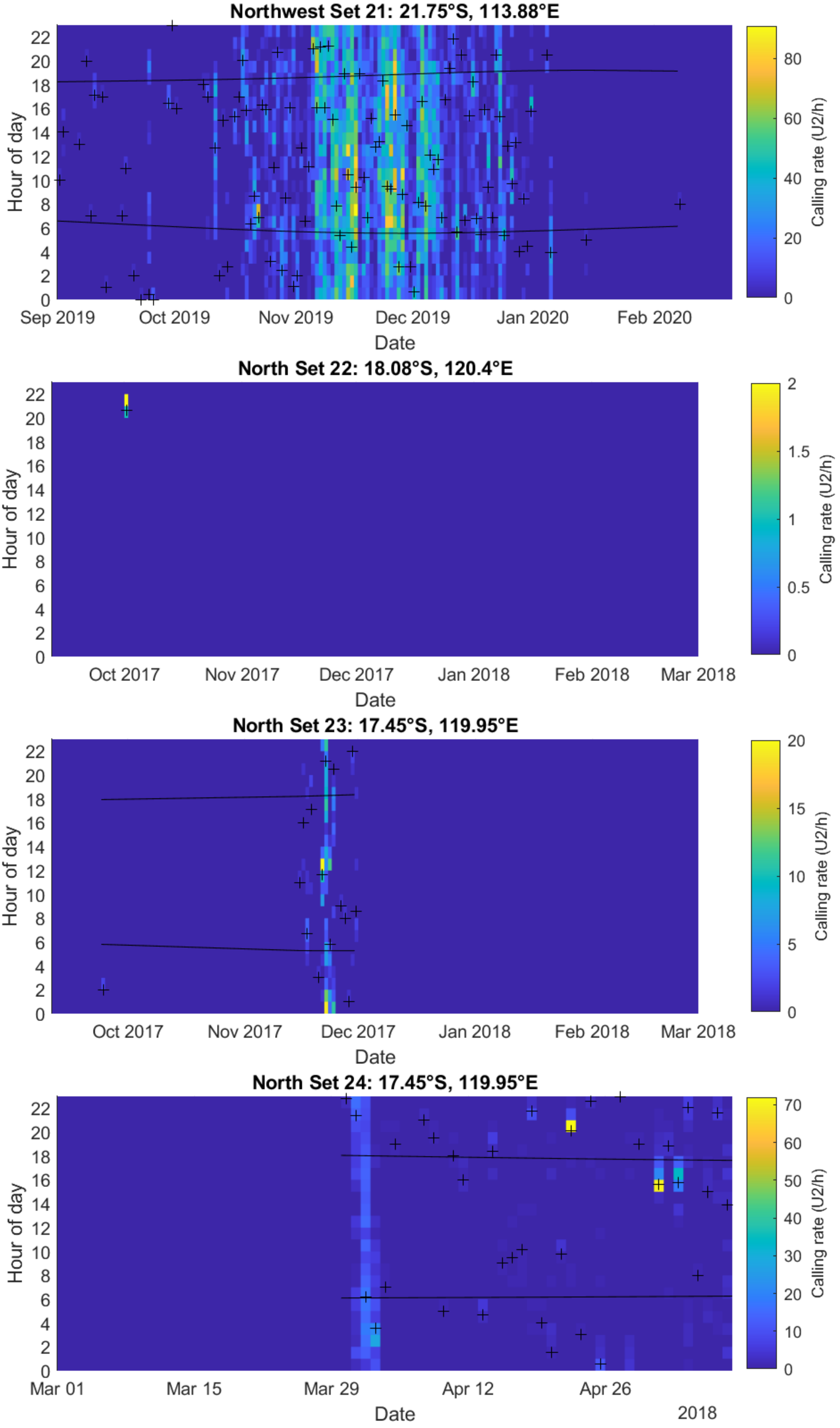
Northwest Set 21: 21.75°S, 113.88°E
Hour of day
Sep 2019
Oct 2019
Nov 2019
Dec 2019
Jan 2020
Feb 2020
Date
Calling rate (U2/h)
North Set 22: 18.08°S, 120.4°E
Hour of day
Oct 2017
Nov 2017
Dec 2017
Jan 2018
Feb 2018
Mar 2018
Date
Calling rate (U2/h)
North Set 23: 17.45°S, 119.95°E
Hour of day
Oct 2017
Nov 2017
Dec 2017
Jan 2018
Feb 2018
Mar 2018
Date
Calling rate (U2/h)
North Set 24: 17.45°S, 119.95°E
Hour of day
Mar 01
Mar 15
Mar 29
Apr 12
Apr 26
2018
Date
Calling rate (U2/h)

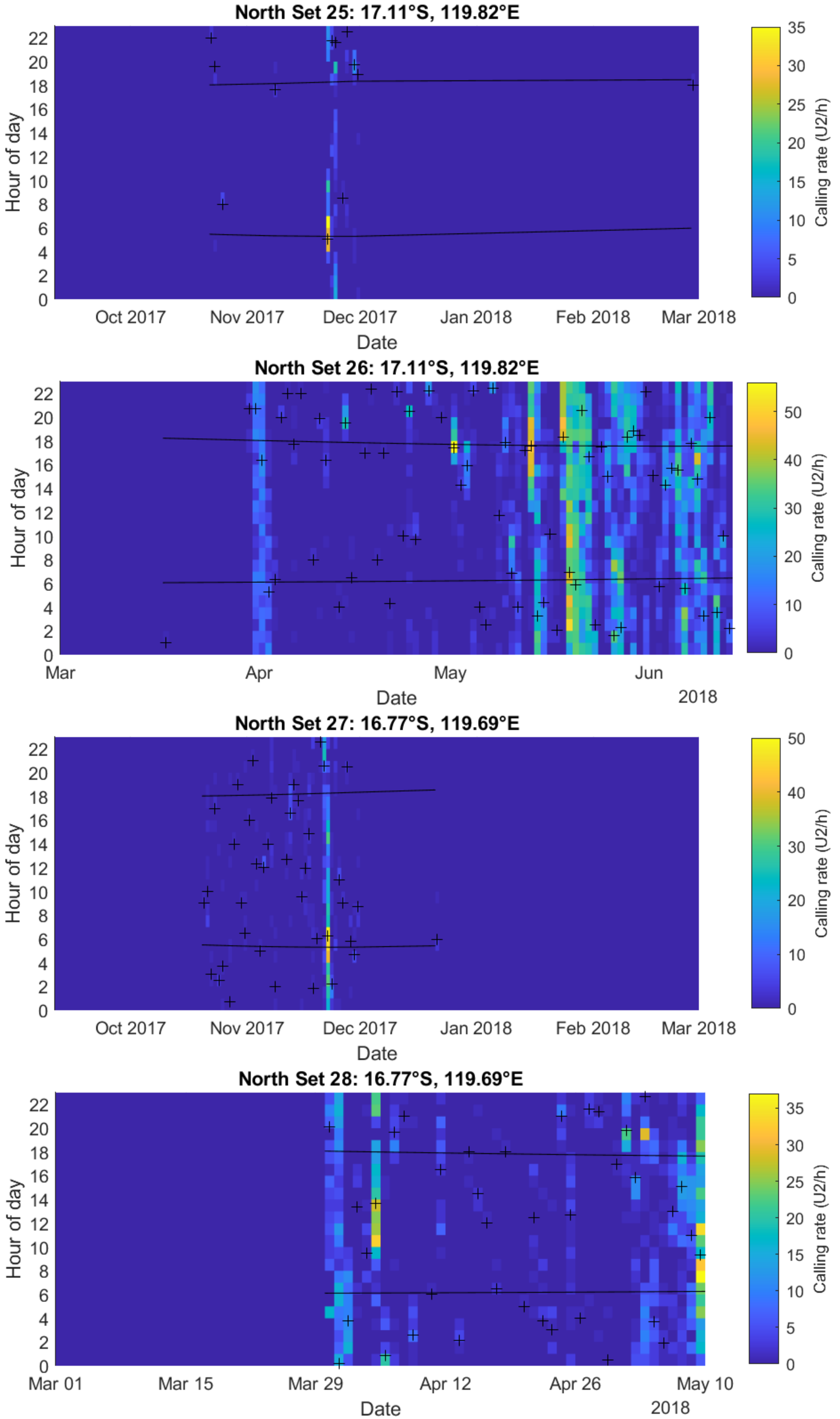
North Set 25: 17.11°S, 119.82°E
Hour of day
Date
Oct 2017
Nov 2017
Dec 2017
Jan 2018
Feb 2018
Mar 2018
Calling rate (U2/h)
North Set 26: 17.11°S, 119.82°E
Hour of day
Date
Mar
Apr
May
Jun
2018
Calling rate (U2/h)
North Set 27: 16.77°S, 119.69°E
Hour of day
Date
Oct 2017
Nov 2017
Dec 2017
Jan 2018
Feb 2018
Mar 2018
Calling rate (U2/h)
North Set 28: 16.77°S, 119.69°E
Hour of day
Date
Mar 01
Mar 15
Mar 29
Apr 12
Apr 26
May 10
2018
Calling rate (U2/h)

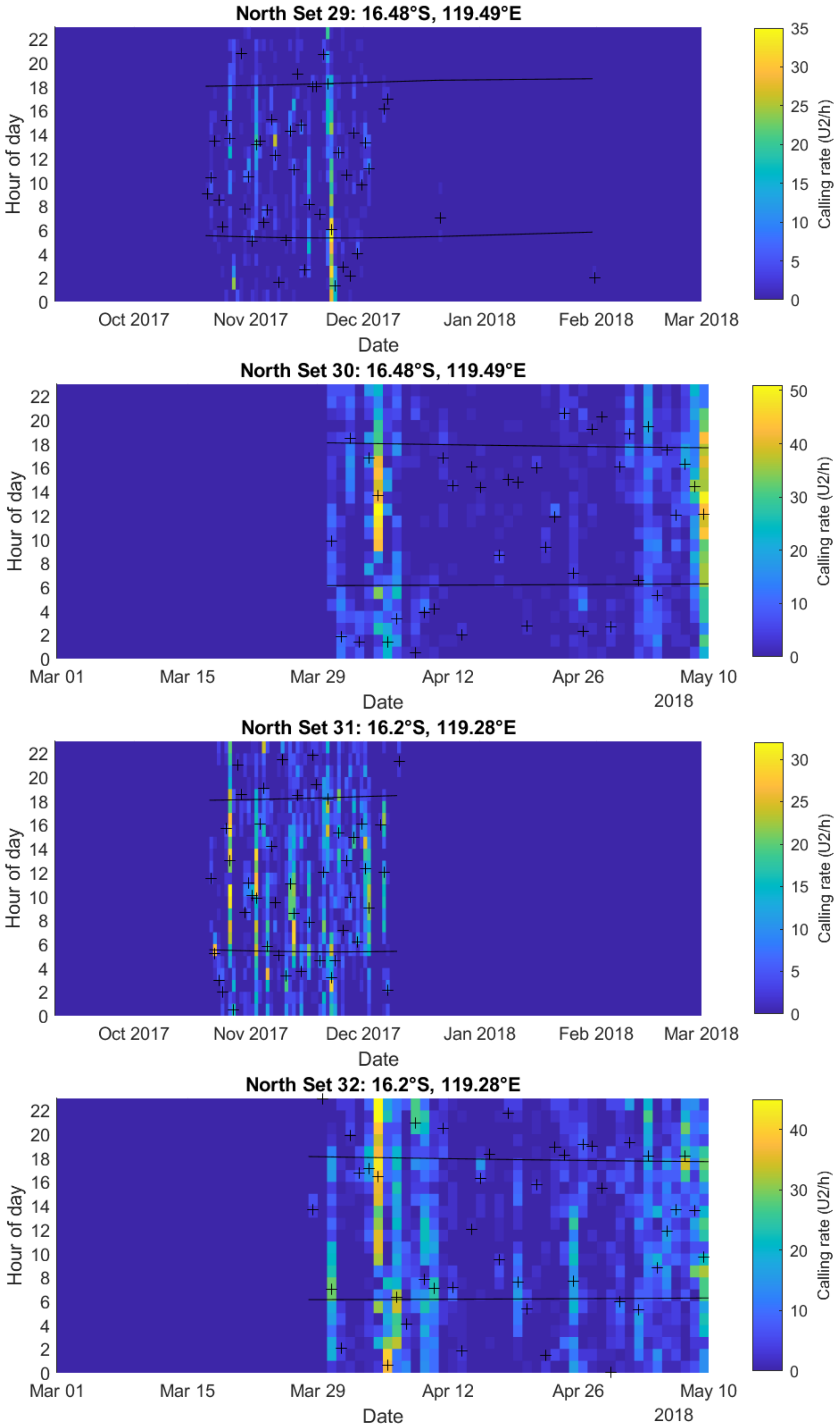
North Set 29: 16.48°S, 119.49°E
North Set 30: 16.48°S, 119.49°E
North Set 31: 16.2°S, 119.28°E
North Set 32: 16.2°S, 119.28°E
Hour of day
Date
Calling rate (U2/h)
Oct 2017
Nov 2017
Dec 2017
Jan 2018
Feb 2018
Mar 2018
Mar 01
Mar 15
Mar 29
Apr 12
Apr 26
May 10
2018

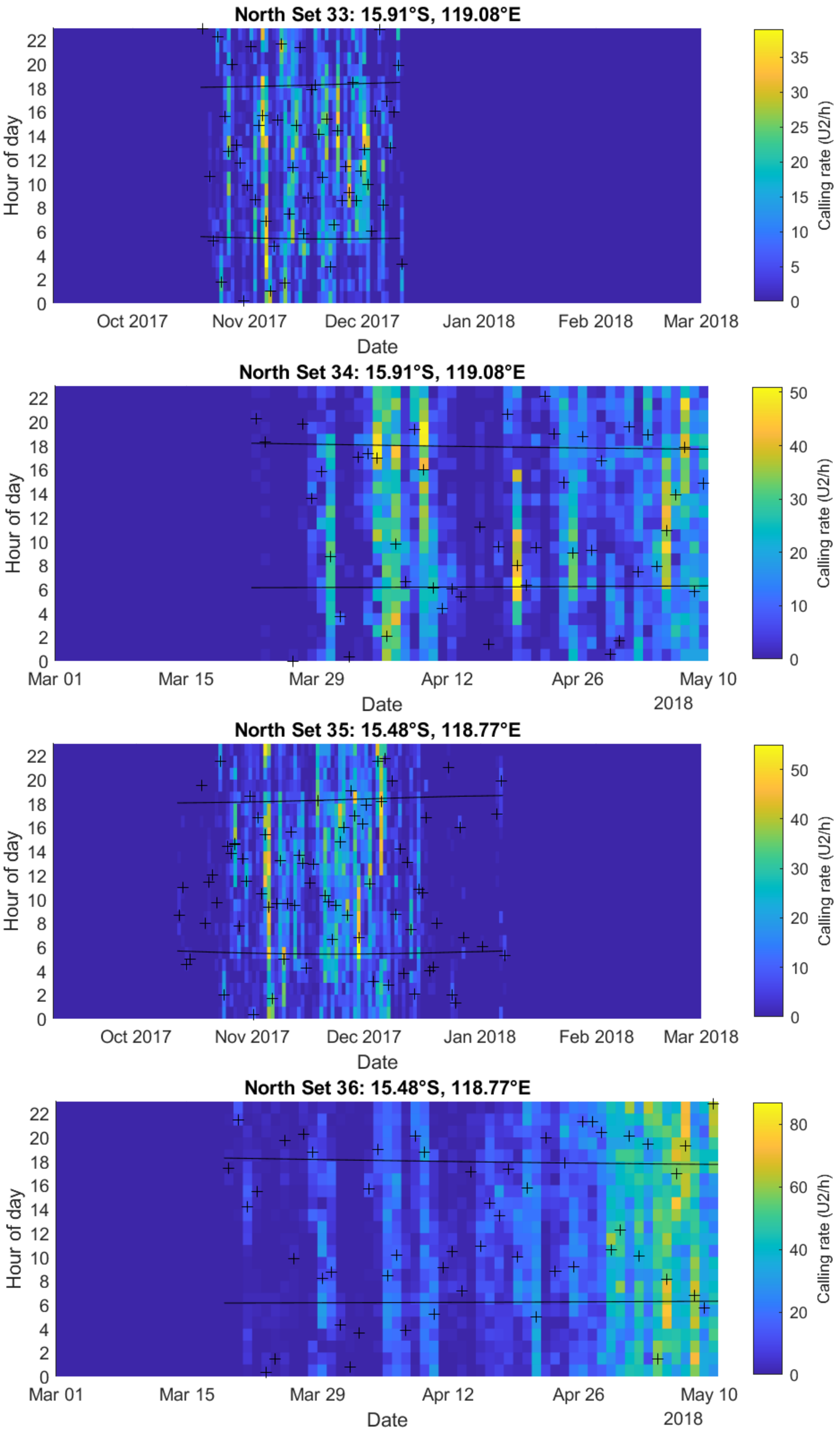
North Set 33: 15.91°S, 119.08°E
Hour of day
Date
Oct 2017
Nov 2017
Dec 2017
Jan 2018
Feb 2018
Mar 2018
Calling rate (U2/h)
North Set 34: 15.91°S, 119.08°E
Hour of day
Date
Mar 01
Mar 15
Mar 29
Apr 12
Apr 26
May 10
2018
Calling rate (U2/h)
North Set 35: 15.48°S, 118.77°E
Hour of day
Date
Oct 2017
Nov 2017
Dec 2017
Jan 2018
Feb 2018
Mar 2018
Calling rate (U2/h)
North Set 36: 15.48°S, 118.77°E
Hour of day
Date
Mar 01
Mar 15
Mar 29
Apr 12
Apr 26
May 10
2018
Calling rate (U2/h)
